# Photoinduced Synthesis of Sulfonyl-Containing Phosphorothioates via a Three-Component Reaction

**DOI:** 10.3390/molecules28237869

**Published:** 2023-11-30

**Authors:** Xianda Wu, Minghong Chen, Shuiyun Zheng, Jie Wu, Gang Liu, Fu-Sheng He

**Affiliations:** 1 Jiangxi Key Laboratory of Organic Chemistry, Jiangxi Science & Technology Normal University, Nanchang 330013, China; 15605068979@163.com; 2 School of Pharmaceutical and Chemical Engineering & Institute for Advanced Studies, Taizhou University, Taizhou 318000, China; 18268652167@163.com (M.C.); shuiyun1109@163.com (S.Z.); 3 State Key Laboratory of Organometallic Chemistry, Shanghai Institute of Organic Chemistry, Chinese Academy of Sciences, 345 Lingling Road, Shanghai 200032, China; 4 School of Chemistry and Chemical Engineering, Henan Normal University, Xinxiang 453007, China

**Keywords:** photoredox catalysis, sulfonyl, phosphorothioate, alkene difunctionalization, multicomponent reaction, copper

## Abstract

Both sulfonyl and phosphorothioate are important privileged structural motifs which are widely presented in pharmaceuticals and agrochemicals. Herein, we describe an efficient approach to synthesizing sulfonyl-containing phosphorothioates by merging photoredox and copper catalysis at room temperature. This protocol is compatible with a wide range of substrates and can be applied to the late-stage modification of complex molecules. Control experiments are conducted to demonstrate the generation of the sulfonyl radical in the transformation.

## 1. Introduction

In recent years, phosphorothioates have received continued attention owing to their remarkable biological activities and broad applications in the pharmaceutical and agrochemical industries [1,2,3,4]. These compounds not only can be used as potential therapeutic molecules, anticancer agents, antivirals and pesticides but also serve as valuable building blocks in organic synthesis [5,6,7,8,9]. In this context, numerous methodologies have been reported for their synthesis [10]. On the other hand, as a prominent class of organosulfur compounds, sulfones play a pivotal role in various drugs, pesticides and natural products [11,12,13,14,15,16,17]. To date, methods for the construction of sulfonyl compounds from sulfonyl chlorides, sodium sulfinates or sulfur dioxide surrogates have attracted considerable attention, and significant progress has been made in this area [18,19,20,21,22,23,24]. However, despite these achievements, the preparation of sulfonyl-containing phosphorothioates has been rarely explored.

**Scheme 1:**
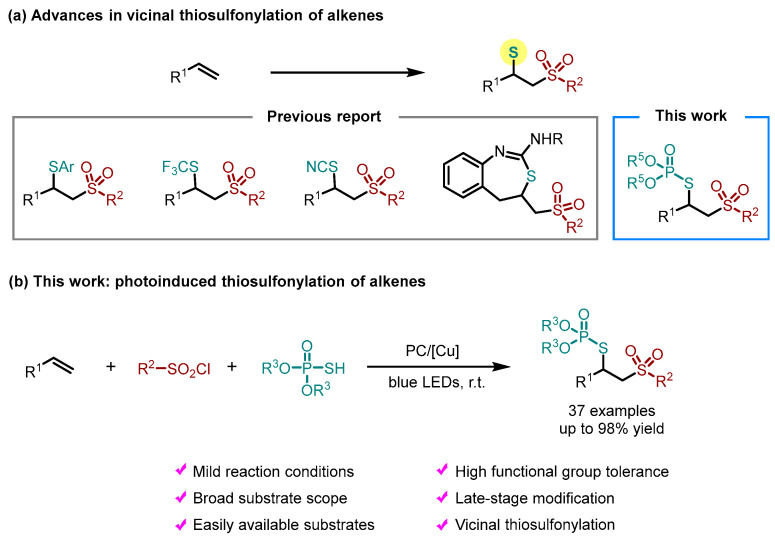
The development of alkene thiosulfonylation.

In the last few decades, the direct vicinal difunctionalization of alkenes has emerged as a powerful strategy to access complex molecules, which could introduce two functional groups into the double bond simultaneously [25,26,27,28,29,30,31,32]. Accordingly, substantial efforts have been devoted to the construction of sulfonyl-containing compounds via the difunctionalization of alkenes, such as hydrosulfonation, oxysulfonation, aminosulfonylation and selenosulfonation [33,34,35]. However, only limited examples have been developed for the thiosulfonylation of alkenes involving the simultaneous installation of two C–S bonds (Scheme 1a). For example, Xu et al. reported a thiosulfonylation reaction of alkenes in the presence of a dual gold/photoredox catalysis system [36,37]. Lian and co-workers established a thiocyanatosulfonation of α,β-unsaturated amides/esters using TMSNCS, aryldiazonium tetrafluoroborates and sulfur dioxide to access β-thiocyanated sulfone compounds [38]. Our group also disclosed an intramolecular thiosulfonylation of alkenes to deliver sulfonated [3,1]-benzothiazepines under metal-free conditions [39]. Given the importance of phosphorothioates and sulfones in organic and medicinal chemistry, we hypothesized that the introduction of these two functional groups into alkenes using the vicinal difunctionalization strategy would contribute to synthetic community and drug discovery. Herein, we describe a mild and efficient method to synthesize sulfonyl-containing phosphorothioates via the thiosulfonylation of alkenes using sulfonyl chlorides and phosphorothioic acids (Scheme 1b). In the presence of photoredox and copper catalysis, this practical reaction features a broad substrate scope and can be readily applied to the late-stage functionalization of bioactive molecules. 

## 2. Results and Discussion

We began our study with the use of 4-vinylbiphenyl **1a**, tosyl chloride **2a** and *O*,*O*-diethyl *S*-hydrogen phosphorothioate **3a** in the presence of Ir(ppy)_3_ and Cu(OTf)_2_ along with K_2_HPO_4_ as the base in DCE under 30 W blue LED irradiation at room temperature. Gratifyingly, we found that the desired product **4a** could be obtained with an excellent yield of 99% (93% isolated yield, Table 1, entry 1). The control experiments showed that no reaction occurred in the absence of Ir(ppy)_3_ or light irradiation, confirming that both the photocatalyst and visible light were essential for this transformation (Table 1, entries 2–3). Without Cu(OTf)_2_ in the reaction system, the yield of **4a** was diminished significantly (Table 1, entry 4). When changing the photocatatlyst to Ir(ppy)_2_(dtbbpy)PF_6_, the corresponding product was achieved with a comparable yield (Table 1, entry 5). Similarly, the use of other copper catalysts such as CuCl_2_ and Cu(CH_3_CN)_4_PF_6_ also underwent the reaction efficiently (Table 1, entries 6–7). Replacing K_2_HPO_4_ with other bases (KHCO_3_, K_2_CO_3_ and Na_2_CO_3_) produced inferior results (Table 1, entries 8–10). Finally, the effect of solvent was examined, and the reaction in THF and CH_3_CN resulted in moderate yields (Table 1, entries 11–12).

**Figure 1:**
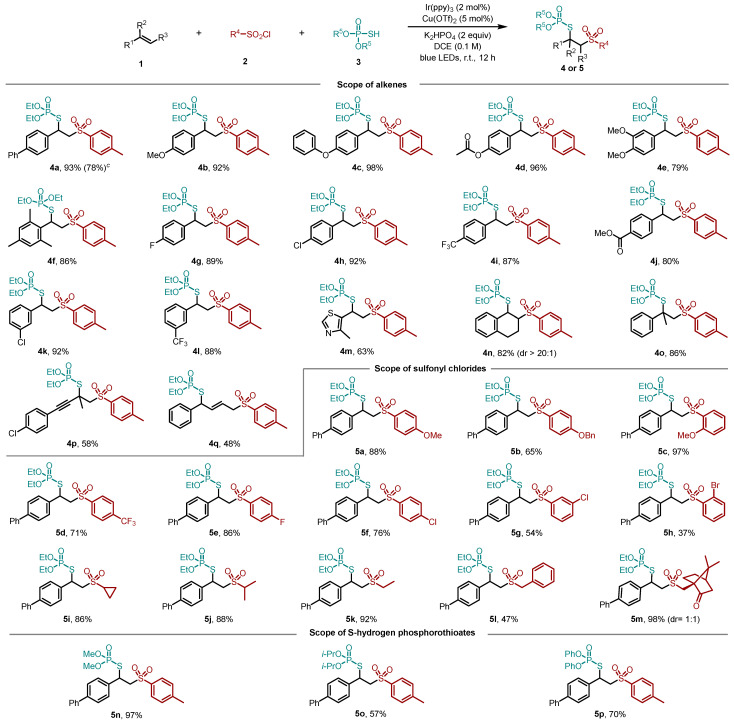
Substrate scope for the photoinduced synthesis of sulfonyl-containing phosphorothioates *^a,b^. ^a^* Reactions conditions: alkene **1** (0.2 mmol), sulfonyl chloride **2** (0.4 mmol), phosphorothioate **3** (0.4 mmol), Ir(ppy)_3_ (2 mol%), Cu(OTf)_2_ (5 mol%), K_2_HPO_4_ (0.4 mmol), DCE (2 mL), irradiation with 30 W blue LEDs at room temperature for 12 h. *^b^* Isolated yield based on alkene **1**.

With the optimized reaction conditions established, we turned our attention to investigating the scope and generality of the three-component vicinal difunctionalization of alkenes (Figure 1 and Supplementary Materials). To verify the scalability of this protocol, the model reaction was carried out on a 6 mmol scale to obtain the product **4a** with a 78% yield. In general, a range of alkenes bearing both electron-donating and electron-withdrawing substituents on the aromatic ring reacted smoothly to provide the products **4b**–**4l** with moderate to good yields. Functional groups such as methyl, methoxy, fluoro, chloro, trifluoromethyl and ester were all well tolerated in this transformation. Notably, a heteroaromatic alkene containing a thiazole group could also be used to provide the corresponding product **4m** at a 63% yield. It is worth mentioning that different types of alkenes including 1,2-dihydronaphthalene, α-methyl styrene, 1,3-enyne and 1,3-diene could furnish the desired compounds **4n**–**4q** in 48–86% yields.

Then, we explored the substrate scope of commercially available aromatic and aliphatic sulfonyl chlorides. As expected, various sulfonyl chlorides with substituents on the aromatic ring underwent the reaction efficiently, giving the corresponding sulfonyl-containing phosphorothioates **5a**–**5h** with moderate to good yields. Remarkably, the aliphatic sulfonyl chlorides were also suitable substrates to deliver the desired products **5i**–**5m** in reasonable yields. In addition, the scope of other alkoxy- or aryloxy-substituted phosphorothioic acids was evaluated and it was found that *O*,*O*-dimethoxy, diisopropoxy and diphenoxy phosphorothioic acid reacted well to afford products **5n**–**5p** in 57–97% yields.

**Scheme 2:**
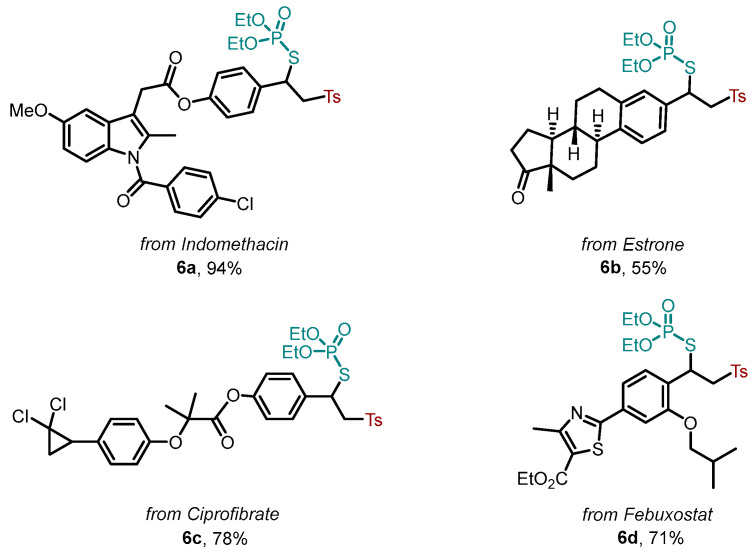
Late-stage functionalization of bioactive molecules.

Furthermore, the robustness of this three-component protocol was evaluated via the late-stage functionalization of bioactive molecules. As shown in Scheme 2, the alkenes derived from indomethacin, estrone, ciprofibrate and febuxostat were found to be compatible under the standard conditions to deliver the desired products **6a**–**6d** in 55–94% yields.

**Scheme 3:**
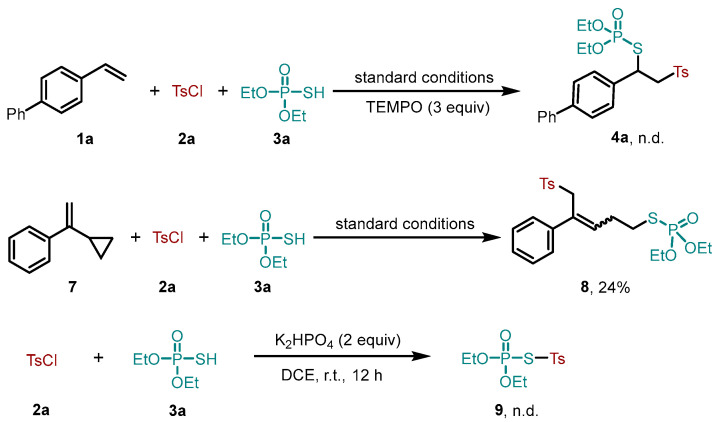
Control experiments.

To illustrate the mechanism for this visible-light-mediated reaction, some additional control experiments were performed (Scheme 3). First, when three equivalents of radical scavenger (2,2,6,6-tetramethylpiperidin-1-yl)oxyl (TEMPO) were added to the model reaction under the standard conditions, the formation of product **4a** was completely suppressed, suggesting that a free radical pathway might be involved in this reaction. Equally, a radical clock experiment was performed using vinyl cyclopropane **7**, and the ring-opening product **8** was obtained with a 24% yield. Taken together, these findings support the generation of sulfonyl radicals under light irradiation. We also conducted a reaction between tosyl chloride **2a** and *O*,*O*-diethyl *S*-hydrogen phosphorothioate **3a** in the presence of K_2_HPO_4_, but the formation of benzenethiosulfonate **9** was not detected. This result indicated that both sulfonyl radicals and sulfenyl radicals were not likely generated by the homolytic cleavage of benzenethiosulfonates.

**Scheme 4:**
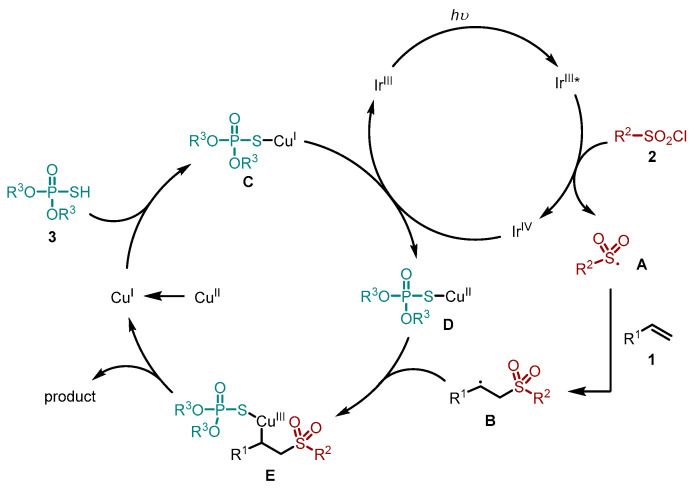
Proposed reaction mechanism.

On the basis of the above results and the literature reports, a plausible mechanism for this three-component protocol is depicted in Scheme 4. Initially, the visible-light-excited Ir(III)* undergoes single-electron transfer with sulfonyl chloride **2** to generate the sulfonyl radical **A**, which then adds to the double bond of alkene **1** to form the alkyl radical intermediate **B**. Meanwhile, the reaction of the copper catalyst with phosphorothioic acid **3** affords the Cu^I^ complex **C**, followed by single-electron oxidation with Ir (IV) to give the Cu^II^ intermediate **D** and regenerate the photocatalyst. Subsequently, the alkyl radical intermediate **B** is captured by the Cu^II^ intermediate **D** to form the Cu^III^ species **E**. Finally, the desired product is delivered via the reductive elimination of **E**, along with releasing the Cu^I^ catalyst. 

## 3. Materials and Methods

### 3.1. General Information

Unless otherwise stated, all commercial reagents were used as received. All solvents were dried and distilled according to the standard procedures. Flash column chromatography was performed using silica gel (60 Å pore size, 32–63 μm, standard-grade). Analytical thin-layer chromatography was performed using glass plates pre-coated with 0.25 mm 230–400 mesh silica gel impregnated with a fluorescent indicator (254 nm). Thin-layer chromatography plates were visualized using exposure to ultraviolet light. The nuclear magnetic resonance (NMR) spectra were recorded in parts per million from internal tetramethylsilane on the δ scale. The ^1^H, ^13^C and ^19^F NMR spectra were recorded in CDCl_3_ using a Bruker DRX 400 spectrometer (Bruker, Fallanden, Switzerland) operating at 400 MHz, 100 MHz and 376 MHz, respectively. All chemical shift values are quoted in ppm and coupling constants quoted in Hz. High-resolution mass spectrometry (HRMS) spectra were obtained using a micrOTOF II instrument.

### 3.2. General Procedure for the Synthesis of Sulfonyl-Containing Phosphorothioates

An oven-dried flask was charged with alkene **1** (0.2 mmol, 1.0 equiv), sulfonyl chloride **2** (0.4 mmol, 2 equiv), K_2_HPO_4_ (0.4 mmol, 2 equiv), Cu(OTf)_2_ (5 mol%) and Ir(ppy)_3_ (2 mol%) under a nitrogen atmosphere. Then, anhydrous DCE (2 mL) and *O*,*O*-diethyl *S*-hydrogen phosphorothioate **3** (0.4 mmol, 2 equiv) were added to the flask. The mixture was placed around 30 W blue LEDs at a distance of ~5 cm and stirred under blue light irradiation for 12 h at room temperature. After completion of the reaction as monitored using TLC analysis, the mixture was filtered through a celite pad, and washed using ethyl acetate; then, the filtrate was evaporated and the residue was purified using flash column chromatography on silica gel (petroleum ether/ethyl acetate = 2:1) to give the corresponding product.

### 3.3. General Procedure for the Scale-Up Experiment

An oven-dried flask was charged with 4-vinyl-1,1′-biphenyl **1a** (6 mmol, 1.0 equiv), 4-methylbenzenesulfonyl chloride **2a** (12 mmol, 2 equiv), K_2_HPO_4_ (12 mmol, 2 equiv), Cu(OTf)_2_ (5 mol%) and Ir(ppy)_3_ (2 mol%) under a nitrogen atmosphere. Then, anhydrous DCE (60 mL) and *O*,*O*-diethyl *S*-hydrogen phosphorothioate **3a** (6 mmol, 2 equiv) were added to the flask. The mixture was placed around 30 W blue LEDs at a distance of ~5 cm and stirred under blue light irradiation for 12 h at room temperature. After completion of the reaction as monitored using TLC analysis, the mixture was filtered through a celite pad, and washed with ethyl acetate; then, the filtrate was evaporated and the residue was purified using flash column chromatography on silica gel (petroleum ether/ethyl acetate = 2:1) to give the corresponding product **4a** with a 78% yield (2.342 g).

## 4. Conclusions

In conclusion, we have developed a visible-light-mediated three-component reaction of alkenes, sulfonyl chlorides and phosphorothioates under blue LED irradiation at room temperature. This efficient protocol shows a broad substrate scope, high functional group tolerance and excellent chemoselectivity. Under the mild reaction conditions, a variety of sulfonyl-containing phosphorothioates were obtained with moderate to good yields. Moreover, the gram-scale synthetic capability and late-stage functionalization of the bioactive molecules demonstrated the applicability of this methodology.

## Figures and Tables

**Table 1:** Optimization of the reaction conditions *^a^*.

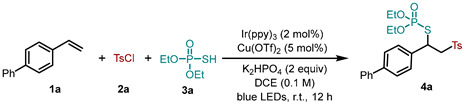		
Entry	Variation from Standard Conditions	Yield (%) *^b^*
1	None	99 (93) *^c^*
2	Without Ir(ppy)_3_	trace
3	In the dark	trace
4	Without Cu(OTf)_2_	59
5	Ir(ppy)_2_(dtbbpy)PF_6_ instead of Ir(ppy)_3_	94
6	CuCl_2_ instead of Cu(OTf)_2_	97
7	Cu(CH_3_CN)_4_PF_6_ instead of Cu(OTf)_2_	93
8	KHCO_3_ instead of K_2_HPO_4_	59
9	K_2_CO_3_ instead of K_2_HPO_4_	33
10	Na_2_CO_3_ instead of K_2_HPO_4_	66
11	THF instead of DCE	53
12	CH_3_CN instead of DCE	54

*^a^* Reactions conditions: **1a** (0.2 mmol), **2a** (0.4 mmol), **3a** (0.4 mmol), Ir(ppy)_3_ (2 mol%), Cu(OTf)_2_ (5 mol%), K_2_HPO_4_ (0.4 mmol), DCE (2 mL), irradiation with 30 W blue LEDs at room temperature for 12 h. *^b^* Yields were determined using ^1^H NMR analysis using dibromomethane as an internal standard. *^c^* Isolated yield.

## Data Availability

The data presented in this study are contained in the article tables and Supplementary Materials.

## Supplementary Materials

The following supporting information can be downloaded at https://www.mdpi.com/article/10.3390/molecules28237869/s1, including the characterization data and ^1^H, ^13^C and ^19^F NMR spectra of products **4**, **5**, **6**.
