# Supplementary material for: Photoinduced Synthesis of Sulfonyl-Containing Phosphorothioates via a Three-Component Reaction
Source: Molecules. 2023 Nov 30;28(23):7869. doi: 10.3390/molecules28237869 (PMC10708487; doi:10.3390/molecules28237869)

Supporting Information

Photoinduced synthesis of sulfonyl-containing phosphorothioates through a three-component reaction

Xianda Wu,[1] Minghong Chen,[2] Shuiyun Zheng,[2] Jie Wu,[*,2,3,4] Gang Liu,[*,1] and Fu-Sheng He[*,2]

[1] *Jiangxi Key Laboratory of Organic Chemistry, Jiangxi Science & Technology Normal University, Nanchang 330013, China*

[2] *School of Pharmaceutical and Chemical Engineering & Institute for Advanced Studies, Taizhou University, Jiaojiang 318000, Zhejiang, China*

[3] *State Key Laboratory of Organometallic Chemistry, Shanghai Institute of Organic Chemistry, Chinese Academy of Sciences, 345 Lingling Road, Shanghai 200032, China*

[4] *School of Chemistry and Chemical Engineering, Henan Normal University, Xinxiang 453007, China*

E-mail: jie_wu@fudan.edu.cn; liugang0926@163.com;hefs@tazc.edu.cn

Table of Contents

1. General Experimental Procedure and Characterization Data……………………S2
2. Scale-Up Experiment………………………………………………………… S21
3. Copies of ^{1}H, ^{13}C and ^{19}F NMR Spectra………………………………………S22

1. General Experimental Procedure and Characterization Data

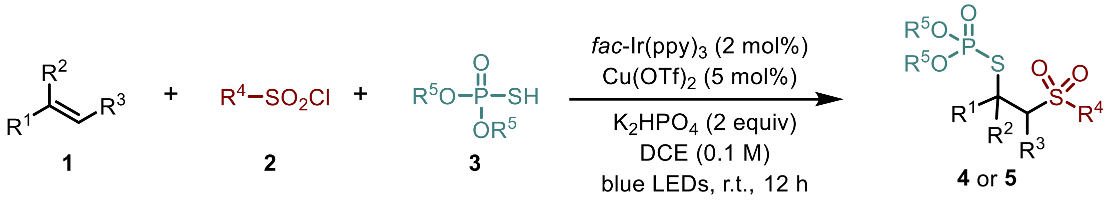

An oven-dried flask was charged with alkene **1** (0.2 mmol, 1.0 equiv), sulfonyl chloride **2** (0.4 mmol, 2 equiv), K_2HPO_4 (0.4 mmol, 2 equiv), $Cu(OTf)_2$ (5 mol%) and *fac*-Ir(ppy)$_3$ (2 mol%) under nitrogen atmosphere. Then anhydrous DCE (2 mL) and *S*-hydrogen phosphorothioate **3** (0.4 mmol, 2 equiv) were added to the flask. The mixture was placed around a 30 W blue LEDs at a distance of ~5 cm and stirred under blue light irradiation for 12 hours at room temperature. After completion of reaction as monitored by TLC analysis, the mixture was filtered through a celite pad, and washed with ethyl acetate, then the filtrate was evaporated and the residue was purified directly by flash column chromatography on silica gel (petroleum ether/ethyl acetate = 2:1) to give the corresponding product **4** or **5**.

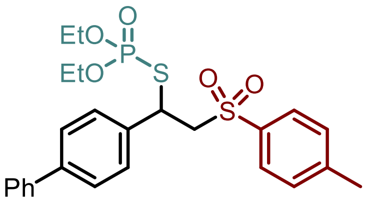

***S*-(1-([1,1'-Biphenyl]-4-yl)-2-tosylethyl) *O,O*-diethyl phosphorothioate (4a):** purified by column chromatography (petroleum ether/ethyl acetate = 2:1), white solid, 93% yield. ^{1}H NMR (400 MHz, $CDCl_3$) δ 7.54 – 7.47 (m, 4H), 7.44 (t, *J* = 7.5 Hz, 2H), 7.41 – 7.34 (m, 3H), 7.25 (m, 2H), 7.13 (d, *J* = 8.1 Hz, 2H), 4.78 (ddd, *J* = 12.5, 10.3, 4.7 Hz, 1H), 4.15 – 3.99 (m, 4H), 3.99 – 3.82 (m, 2H), 2.31 (s, 3H), 1.30 (t, *J* = 7.1 Hz, 3H), 1.20 (t, *J* = 7.1 Hz, 3H); ^{13}C NMR (100 MHz, $CDCl_3$) δ 144.47, 141.21, 140.24, 137.30 (d, *J* = 5.1 Hz), 136.38, 129.61, 128.87, 128.23, 128.00, 127.65, 127.29, 126.95, 63.97 (dd, *J* = 16.4, 6.1 Hz), 61.96 (d, *J* = 5.9 Hz), 44.04 (d, *J* = 3.5 Hz), 21.55, 15.93 (dd, *J* = 15.5, 7.4 Hz). HRMS (ESI) calcd for $C_{25}H_{29}O_5NaPS_2^+$ (M+Na$^+$): 527.1092, found: 527.1101

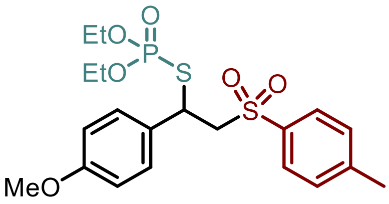

***O,O*-Diethyl *S*-(1-(4-methoxyphenyl)-2-tosylethyl) phosphorothioate (4b):** purified by column chromatography (petroleum ether/ethyl acetate = 2:1), colorless oil, 92% yield. ^{1}1H NMR (400 MHz, $CDCl_3$) δ 7.51 (d, *J* = 8.2 Hz, 2H), 7.17 (d, *J* = 8.1 Hz, 2H), 7.10 (d, *J* = 8.7 Hz, 2H), 6.70 (d, *J* = 8.6 Hz, 2H), 4.70 (ddd, *J* = 12.0, 10.6, 4.6 Hz, 1H), 4.13 – 3.97 (m, 4H), 3.92 – 3.83 (m, 2H), 3.75 (s, 3H), 2.38 (s, 3H), 1.29 (t, *J* = 7.1 Hz, 3H), 1.21 (t, *J* = 7.1 Hz, 3H); ^{13}C NMR (100 MHz, $CDCl_3$) δ 159.49, 144.42, 136.41, 130.23 (d, *J* = 5.6 Hz), 129.60, 128.92, 127.97, 114.00, 63.88 (dd, *J* = 14.1, 6.1 Hz), 62.07 (d, *J* = 5.5 Hz), 55.28, 43.92 (d, *J* = 3.5 Hz), 21.55, 15.92 (dd, *J* = 11.1, 7.3 Hz); HRMS (ESI) calcd for $C_{20}H_{27}O_6NaPS_2^+$ (M+Na^+): 481.0884, found: 481.0886.

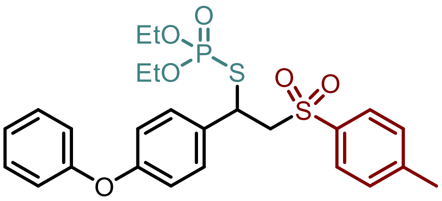

***O,O*-Diethyl *S*-(1-(4-phenoxyphenyl)-2-tosylethyl) phosphorothioate (4c) :** purified by column chromatography (petroleum ether/ethyl acetate = 2:1), white solid, 98% yield. ^{1}H NMR (400 MHz, $CDCl_3$) δ 7.55 (d, *J* = 8.2 Hz, 2H), 7.35 (t, *J* = 7.9 Hz, 2H), 7.21 (d, *J* = 8.0 Hz, 2H), 7.18 – 7.10 (m, 3H), 6.98 (d, *J* = 7.8 Hz, 2H), 6.80 (d, *J* = 8.6 Hz, 2H), 4.73 (ddd, *J* = 12.5, 10.3, 4.6 Hz, 1H), 4.14 – 3.98 (m, 4H), 3.95 – 3.83 (m, 2H), 2.40 (s, 3H), 1.30 (t, *J* = 7.1 Hz, 3H), 1.23 (t, *J* = 7.1 Hz, 3H); ^{13}C NMR (100 MHz, $CDCl_3$) δ 157.47, 156.33, 144.61, 136.45, 132.89 (d, *J* = 5.4 Hz), 129.89, 129.69, 129.20, 128.03, 123.88, 119.34, 118.31, 63.94 (dd, *J* = 13.6, 6.1 Hz), 62.03 (d, *J* = 5.6 Hz), 43.80 (d, *J* = 3.5 Hz), 21.65, 15.97 (dd, *J* = 10.2, 7.4 Hz); HRMS (ESI) calcd for $C_{25}H_{29}O_6NaPS_2^+$ (M+Na^+): 543.1041, found: 543.1033.

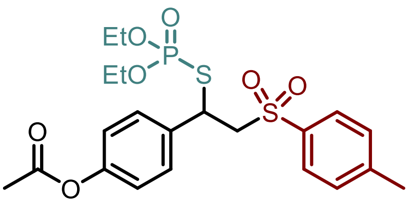

4-(1-((Diethoxyphosphoryl)thio)-2-tosylethyl)phenyl acetate (4d): purified by column chromatography (petroleum ether/ethyl acetate = 2:1), white solid, 96% yield. ^{1}H NMR (400 MHz, $CDCl_3$) δ 7.51 (d, *J* = 8.3 Hz, 2H), 7.25 – 7.16 (m, 4H), 6.95 – 6.88 (m, 2H), 4.75 (ddd, *J* = 12.8, 9.8, 5.0 Hz, 1H), 4.09 – 3.95 (m, 4H), 3.91 – 3.74 (m, 2H), 2.38 (s, 3H), 2.28 (s, 3H), 1.28 (t, *J* = 7.1 Hz, 3H), 1.20 (t, *J* = 7.1 Hz, 3H); ^{13}C NMR (100 MHz, $CDCl_3$) δ 168.97, 150.45, 144.77, 136.20 (d, *J* = 1.0 Hz), 136.14, 129.78, 128.83, 127.92, 121.82, 63.96 (dd, *J* = 12.1, 6.1 Hz), 61.93 (d, *J* = 6.3 Hz), 43.64 (d, *J* = 3.5 Hz), 21.56, 21.10, 15.89 (dd, *J* = 11.8, 7.3 Hz); HRMS (ESI) calcd for $C_{21}H_{27}O_7NaPS_2^+$ (M+Na$^+$): 509.0834, found: 509.0826.

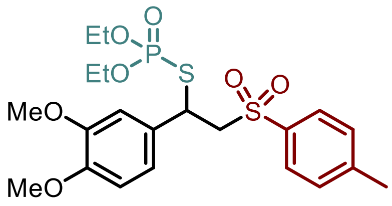

***S*-(1-(3,4-Dimethoxyphenyl)-2-tosylethyl) *O,O*-diethyl phosphorothioate (4e):** purified by column chromatography (petroleum ether/ethyl acetate = 2:1), yellow oil, 79% yield. ^{1}H NMR (400 MHz, $CDCl_3$) δ 7.49 (d, *J* = 8.2 Hz, 2H), 7.16 (d, *J* = 8.1 Hz, 2H), 6.77 (dd, *J* = 8.3, 2.0 Hz, 1H), 6.66 (d, *J* = 8.3 Hz, 1H), 6.61 (d, *J* = 2.0 Hz, 1H), 4.71 (ddd, *J* = 12.9, 10.3, 4.7 Hz, 1H), 4.14 – 3.97 (m, 4H), 3.95 – 3.86 (m, 2H), 3.84 (s, 3H), 3.77 (s, 3H), 2.38 (s, 3H), 1.30 (t, *J* = 7.1 Hz, 3H), 1.23 (t, *J* = 7.1 Hz, 3H); ^{13}C NMR (100 MHz, $CDCl_3$) δ 149.06, 148.86, 144.45, 136.47, 130.52 (d, *J* = 5.6 Hz), 129.46, 127.98, 120.37, 110.83, 110.37,63.95 (dd, *J* = 11.9, 6.2 Hz), 62.15 (d, *J* = 5.2 Hz), 55.90, 55.76, 44.39 (d, *J* = 3.5 Hz), 21.54, 15.95 (dd, *J* = 11.2, 7.3 Hz); HRMS (ESI) calcd for $C_{21}H_{29}O_7NaPS_2^+$ (M+Na$^+$): 511.0990, found: 511.1003.

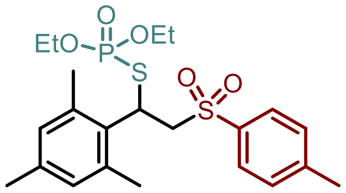

***O,O*-Diethyl *S*-(1-mesityl-2-tosylethyl) phosphorothioate (4f):** purified by column chromatography (petroleum ether/ethyl acetate = 2:1), colorless oil, 86% yield. ^{1}H NMR (400 MHz, $CDCl_3$) 7.47 (d, *J* = 8.2 Hz, 2H), 7.13 (d, *J* = 8.1 Hz, 2H), 6.78 (s, 1H), 6.51 (s, 1H), 5.33 (ddd, *J* = 13.5, 9.5, 5.6 Hz, 1H), 4.20 – 3.83 (m, 6H), 2.44 (s,

3H), 2.37 (s, 3H), 2.22 (s, 3H), 2.18 (s, 3H), 1.35 (t, *J* = 7.1 Hz, 3H), 1.24 (t, *J* = 7.0 Hz, 3H); ^{13}C NMR (100 MHz, $CDCl_3$) δ 144.33, 137.87, 136.99, 136.41, 136.08, 131.42 (d, *J* = 4.9 Hz), 130.87, 129.41, 127.72, 63.98 (dd, *J* = 16.9, 6.1 Hz), 60.69 (d, *J* = 4.4 Hz), 38.69 (d, *J* = 3.7 Hz), 21.59, 21.06 (d, *J* = 1.4 Hz), 20.67, 20.61, 15.96 (dd, *J* = 13.7, 7.4 Hz); HRMS (ESI) calcd for $C_{22}H_{31}O_5NaPS_2^+$ (M+Na$^+$): 493.1248, found: 493.1242.

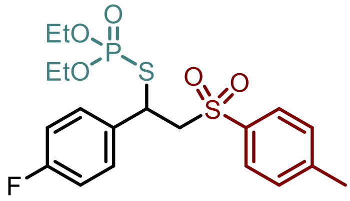

***O*,*O*-Diethyl *S*-(1-(4-fluorophenyl)-2-tosylethyl) phosphorothioate (4g):** purified by column chromatography (petroleum ether/ethyl acetate = 2:1), colorless oil, 89% yield. ^{1}H NMR (400 MHz, $CDCl_3$) δ 7.52 (d, *J* = 8.3 Hz, 2H), 7.23 – 7.15 (m, 4H), 6.89 (t, *J* = 8.6 Hz, 2H), 4.74 (ddd, *J* = 12.6, 10.3, 4.6 Hz, 1H), 4.10 – 3.94 (m, 4H), 3.91 – 3.81 (m, 2H), 2.40 (s, 3H), 1.28 (t, *J* = 7.1 Hz, 3H), 1.21 (t, *J* = 7.1 Hz, 3H); ^{13}C NMR (100 MHz, $CDCl_3$) δ 162.39 (d, *J* = 248.1 Hz), 144.76, 136.31, 134.44 (dd, *J* = 5.1, 3.3 Hz), 129.70, 129.58 (d, *J* = 8.3 Hz), 127.95, 115.58 (d, *J* = 21.8 Hz), 63.98 (dd, *J* = 11.2, 6.2 Hz), 61.97 (d, *J* = 6.0 Hz), 43.50 (d, *J* = 3.5 Hz), 21.56, 15.90 (dd, *J* = 10.3, 7.3 Hz); ^{19}F NMR (376 MHz, $CDCl_3$) δ -113.10 (dq, *J* = 8.5, 5.2 Hz); HRMS (ESI) calcd for $C_{19}H_{24}O_5FNaPS_2^+$ (M+Na$^+$): 469.0684, found: 469.0696.

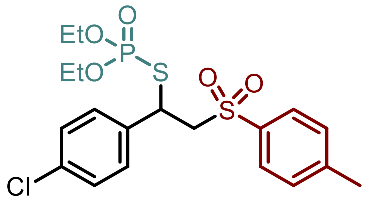

***S*-(1-(4-Chlorophenyl)-2-tosylethyl) *O*,*O*-diethyl phosphorothioate (4h):** purified by column chromatography (petroleum ether/ethyl acetate = 2:1), colorless oil, 92% yield. ^{1}H NMR (400 MHz, $CDCl_3$) δ 7.49 (d, *J* = 8.3 Hz, 2H), 7.19 (d, *J* = 8.1 Hz, 2H), 7.16 – 7.11 (m, 4H), 4.71 (ddd, *J* = 12.7, 10.4, 4.6 Hz, 1H), 4.09 – 3.95 (m, 4H), 3.91 – 3.83 (m, 2H), 2.40 (s, 3H), 1.28 (t, *J* = 7.1 Hz, 3H), 1.21 (t, *J* = 7.1 Hz, 3H); ^{13}C NMR (100 MHz, $CDCl_3$) δ 144.83, 136.99 (d, *J* = 5.1 Hz), 136.17, 134.17, 129.71, 129.17, 128.76, 127.92, 64.04 (dd, *J* = 11.0, 6.2 Hz), 61.76 (d, *J* = 6.0 Hz), 43.54 (d, *J* = 3.4

Hz), 21.59, 15.90 (dd, J = 10.6, 7.3 Hz); HRMS (ESI) calcd for $C_{19}H_{24}O_5NaPS_2Cl^+$ (M+Na$^+$): 485.0389, found: 485.0396.

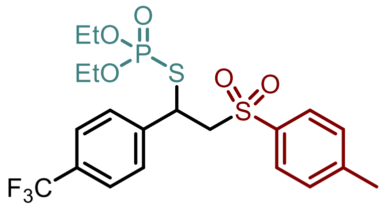

***O,O*-Diethyl *S*-(2-tosyl-1-(4-(trifluoromethyl)phenyl)ethyl) phosphorothioate (4i):** purified by column chromatography (petroleum ether/ethyl acetate = 2:1), white solid, 87% yield. ^{1}H NMR (400 MHz, $CDCl_3$) δ 7.48 – 7.37 (m, 4H), 7.30 (d, J = 8.2 Hz, 2H), 7.12 (d, J = 8.1 Hz, 2H), 4.79 (ddd, J = 13.0, 10.4, 4.6 Hz, 1H), 4.10 – 3.81 (m, 6H), 2.36 (s, 3H), 1.26 (t, J = 7.1 Hz, 3H), 1.20 (t, J = 7.1 Hz, 3H); ^{13}C NMR (100 MHz, $CDCl_3$) δ 144.84, 142.43 (dd, J = 4.7, 1.1 Hz), 136.03, 130.27 (q, J = 32.6 Hz), 129.68, 128.31, 127.86, 125.51 (q, J = 3.7 Hz), 123.74 (q, J = 272.2 Hz), 64.11 (dd, J = 11.0, 6.3 Hz), 61.51 (d, J = 6.3 Hz), 43.62 (d, J = 3.4 Hz), 21.42, 15.84 (dd, J = 12.3, 7.3 Hz); ^{19}F NMR (376 MHz, $CDCl_3$) δ -62.76 (s). HRMS (ESI) calcd for $C_{20}H_{24}O_5NaPS_2F_3^+$ (M+Na$^+$): 519.0653, found: 519.0665.

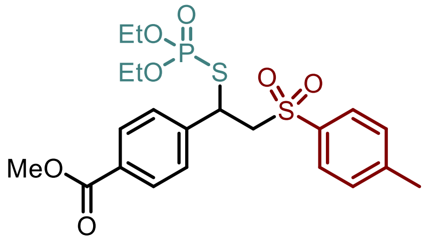

Methyl 4-(1-((diethoxyphosphoryl)thio)-2-tosylethyl)benzoate (4j): purified by column chromatography (petroleum ether/ethyl acetate = 2:1), colorless oil, 80% yield. ^{1}H NMR (400 MHz, $CDCl_3$) δ 7.86 (d, J = 8.3 Hz, 2H), 7.52 (d, J = 8.2 Hz, 2H), 7.28 (d, J = 8.3 Hz, 1H), 7.17 (d, J = 8.1 Hz, 2H), 4.76 (ddd, J = 13.0, 10.1, 4.7 Hz, 1H), 4.10 – 3.96 (m, 4H), 3.93 – 3.80 (m, 5H), 2.37 (s, 3H), 1.27 (t, J = 7.0 Hz, 3H), 1.19 (t, J = 7.1 Hz, 3H); ^{13}C NMR (100 MHz, $CDCl_3$) δ 166.38, 144.87, 143.59 (d, J = 4.7 Hz), 136.10, 129.93, 129.89, 129.74, 127.97, 127.86, 64.06 (dd, J = 12.1, 6.2 Hz), 61.61 (d, J = 6.2 Hz), 52.25 (s), 43.71 (d, J = 3.5 Hz), 21.54 (s), 15.90 (dd, J = 10.8, 7.3 Hz); HRMS (ESI) calcd for $C_{21}H_{27}O_7NaPS_2^+$ (M+Na$^+$): 509.0834, found: 509.0821.

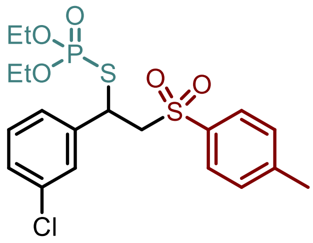

***S*-(1-(3-Chlorophenyl)-2-tosylethyl) *O,O*-diethyl phosphorothioate (4k):** purified by column chromatography (petroleum ether/ethyl acetate = 2:1), white solid, 92% yield. ^{1}H NMR (400 MHz, $CDCl_3$) δ 7.50 (d, *J* = 8.2 Hz, 2H), 7.20 – 7.11 (m, 5H), 7.07 (s, 1H), 4.68 (ddd, *J* = 12.8, 10.3, 4.6 Hz, 1H), 4.13 – 3.93 (m, 4H), 3.92 – 3.83 (m, 2H), 2.38 (s, 3H), 1.29 (t, *J* = 7.1 Hz, 3H), 1.21 (t, *J* = 7.1 Hz, 3H); ^{13}C NMR (100 MHz, $CDCl_3$) δ 144.84, 140.46 (d, *J* = 4.9 Hz), 136.03, 134.44, 129.97, 129.70, 128.31, 127.89, 127.78, 126.19, 64.06 (dd, *J* = 14.2, 6.2 Hz), 61.60 (d, *J* = 6.1 Hz), 43.63 (d, *J* = 3.5 Hz), 21.59, 15.89 (dd, *J* = 13.1, 7.3 Hz); HRMS (ESI) calcd for $C_{19}H_{24}O_5NaPS_2Cl^+$ (M+Na$^+$): 485.0389, found: 485.0388.

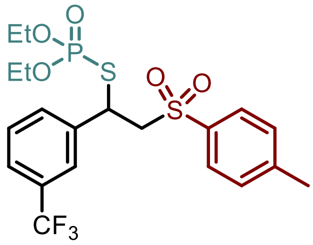

***O,O*-Diethyl *S*-(2-tosyl-1-(3-(trifluoromethyl)phenyl)ethyl) phosphorothioate (4l):** purified by column chromatography (petroleum ether/ethyl acetate = 2:1), colorless oil, 88% yield. ^{1}H NMR (400 MHz, $CDCl_3$) δ 7.48 – 7.42 (m, 4H), 7.37 – 7.33 (m, 2H), 7.14 (d, *J* = 8.1 Hz, 2H), 4.80 (ddd, *J* = 12.8, 10.3, 4.7 Hz, 1H), 4.10 – 3.97 (m, 4H), 3.97 – 3.91 (m, 1H), 3.90 – 3.83 (m, 1H), 2.35 (s, 3H), 1.26 (t, *J* = 7.1 Hz, 3H), 1.19 (t, *J* = 7.1 Hz, 3H); ^{13}C NMR (100 MHz, $CDCl_3$) δ 144.89, 139.67 (d, *J* = 4.6 Hz), 135.99, 131.56 (d, *J* = 0.8 Hz), 130.94 (q, *J* = 32.5 Hz), 129.72, 129.26, 127.84, 124.96 (q, *J* = 3.7 Hz), 124.46 (dd, *J* = 7.6, 3.8 Hz), 123.59 (q, *J* = 272.5 Hz), 64.07 (dd, *J* = 11.8, 6.2 Hz), 61.57 (d, *J* = 6.5 Hz), 43.75 (d, *J* = 3.4 Hz), 21.48, 15.82 (dd, *J* = 12.0, 7.3 Hz); ^{19}F NMR (376 MHz, $CDCl_3$) δ -62.80 (s); HRMS (ESI) calcd for $C_{20}H_{24}O_5F_3NaPS_2^+$ (M+Na$^+$): 519.0653, found: 519.0663.

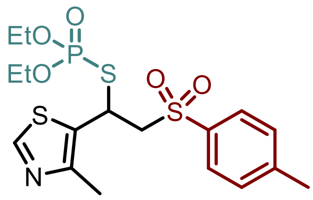

O,O-Diethyl S-(1-(4-methylthiazol-5-yl)-2-tosylethyl) phosphorothioate (4m): purified by column chromatography (petroleum ether/ethyl acetate = 2:1), colorless oil, 63% yield. ^{1}H NMR (400 MHz, $CDCl_3$) δ 8.53 (s, 1H), 7.53 (d, J = 8.2 Hz, 2H), 7.23 (d, J = 8.2 Hz, 2H), 5.16 (td, J = 11.1, 4.1 Hz, 1H), 4.15 – 3.91 (m, 5H), 3.76 (dd, J = 14.5, 10.7 Hz, 1H), 2.50 (s, 3H), 2.40 (s, 3H), 1.32 (t, J = 7.1 Hz, 3H), 1.26 (t, J = 7.1 Hz, 3H); ^{13}C NMR (100 MHz, $CDCl_3$) δ 151.82, 151.14, 144.93, 136.10, 131.16 (d, J = 5.2 Hz), 129.82, 127.77, 64.22 (t, J = 5.9 Hz), 63.39 (d, J = 6.0 Hz), 37.19 (d, J = 3.5 Hz), 21.63, 15.94 (t, J = 7.9 Hz), 15.38; HRMS (ESI) calcd for $C_{17}H_{24}NO_5NaPS_3^+$ (M+Na$^+$): 472.0452, found: 472.0464.

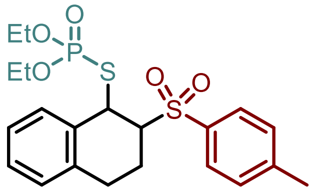

2-((2-(Benzyloxy)ethyl)sulfonyl)-1-phenylethan-1-one (4n): purified by column chromatography (petroleum ether/ethyl acetate = 2:1), white solid, 82% yield (dr > 20:1). ^{1}H NMR (400 MHz, $CDCl_3$) δ 7.76 (d, J = 8.2 Hz, 2H), 7.36 – 7.28 (m, 3H), 7.19 – 7.14 (m, 2H), 7.07 – 7.00 (m, 1H), 4.98 (d, J = 12.2 Hz, 1H), 4.17 – 3.89 (m, 1H), 3.13 (ddd, J = 16.8, 10.2, 6.5 Hz, 1H), 2.86 (ddd, J = 17.0, 6.1, 4.6 Hz, 1H), 2.51 (ddd, J = 15.5, 11.6, 6.2 Hz, 1H), 2.43 (s, 3H), 2.33 (ddd, J = 9.6, 8.1, 4.7 Hz, 1H), 1.32 (t, J = 7.1 Hz, 3H), 1.27 (t, J = 7.1 Hz, 3H); ^{13}C NMR (100 MHz, $CDCl_3$) δ 144.83, 136.29, 135.19, 132.65 (d, J = 8.2 Hz), 130.09, 129.81, 128.91, 128.87, 128.09, 126.52, 64.39 (d, J = 1.2 Hz), 63.99 (dd, J = 24.8, 6.5 Hz), 42.78 (d, J = 3.6 Hz), 24.79, 21.67, 18.96, 16.09 (dd, J = 9.2, 7.1 Hz); HRMS (ESI) calcd for $C_{21}H_{27}O_5NaPS_2^+$ (M+Na$^+$): 477.0935, found: 477.0936.

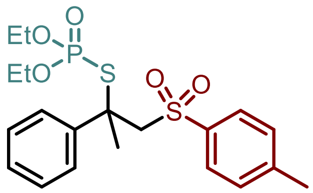

***O,O*-Diethyl *S*-(2-phenyl-1-tosylpropan-2-yl) phosphorothioate (4o):** purified by column chromatography (petroleum ether/ethyl acetate = 2:1), white solid, 86% yield. ^{1}H NMR (400 MHz, $CDCl_3$) δ 7.46 (d, *J* = 8.2 Hz, 2H), 7.43 – 7.37 (m, 2H), 7.23 – 7.17 (m, 3H), 7.14 (d, *J* = 8.1 Hz, 2H), 4.50 (d, *J* = 14.7 Hz, 1H), 4.30 (d, *J* = 14.7 Hz, 1H), 4.13 – 3.96 (m, 3H), 3.96 – 3.85 (m, 1H), 2.37 (d, *J* = 4.7 Hz, 6H), 1.28 (t, *J* = 7.1 Hz, 3H), 1.21 (t, *J* = 7.1 Hz, 3H); ^{13}C NMR (100 MHz, $CDCl_3$) δ 144.16, 140.34 (d, *J* = 8.6 Hz), 137.92, 129.66, 128.26, 127.94, 127.66, 126.76, 66.62 (d, *J* = 2.8 Hz), 64.04, 63.96, 63.89, 54.30 (d, *J* = 4.1 Hz), 26.66 (d, *J* = 3.8 Hz), 21.56, 15.97 (d, *J* = 7.3 Hz); HRMS (ESI) calcd for $C_{20}H_{27}O_5NaPS_2^+$ (M+Na$^+$): 465.0935, found: 465.0948.

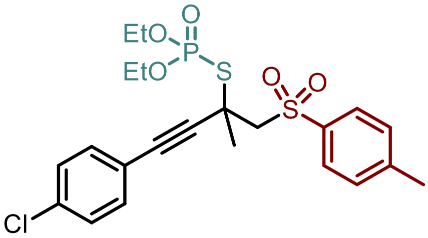

***S*-(4-(4-Chlorophenyl)-1-tosylbut-3-yn-2-yl) *O,O*-diethyl phosphorothioate (4p):** purified by column chromatography (petroleum ether/ethyl acetate = 2:1), colorless oil, 58% yield. ^{1}H NMR (400 MHz, $CDCl_3$) δ 7.82 (d, *J* = 8.3 Hz, 2H), 7.26 – 7.21 (m, 2H), 7.18 (d, *J* = 8.1 Hz, 2H), 7.16 – 7.10 (m, 2H), 4.60 (d, *J* = 14.5 Hz, 1H), 4.28 – 3.97 (m, 4H), 3.83 (d, *J* = 14.5 Hz, 1H), 2.24 (s, 3H), 2.10 (d, *J* = 3.1 Hz, 3H), 1.33 (t, *J* = 7.1 Hz, 3H), 1.17 (t, *J* = 7.1 Hz, 3H); ^{13}C NMR (100 MHz, $CDCl_3$) δ 144.56, 137.27, 134.73, 132.98, 129.68, 128.37, 120.52, 89.19, 89.13, 85.93, 64.95, 64.28 (dd, *J* = 13.8, 6.8 Hz), 43.24 (d, *J* = 4.2 Hz), 29.24 (d, *J* = 9.5 Hz), 21.48, 15.96 (dd, *J* = 25.1, 7.3 Hz); HRMS (ESI) calcd for $C_{22}H_{26}ClO_5NaPS_2^+$ (M+Na$^+$): 523.0757, found: 523.0764.

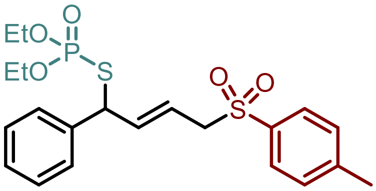

(*E*)-*O,O*-diethyl *S*-(1-phenyl-4-tosylbut-2-en-1-yl) phosphorothioate (4q): purified by column chromatography (petroleum ether/ethyl acetate = 2:1), colorless oil, 48% yield. ^{1}H NMR (400 MHz, $CDCl_3$) δ 7.63 (d, *J* = 8.1 Hz, 2H), 7.35 – 7.27 (m, 3H), 7.26 – 7.17 (m, 4H), 5.81 (dd, *J* = 15.2, 7.6 Hz, 1H), 5.61 (dt, *J* = 15.1, 7.5 Hz, 1H), 4.92 (dd, *J* = 11.2, 7.6 Hz, 1H), 4.20 – 3.86 (m, 4H), 3.85 – 3.69 (m, 2H), 2.41 (s, 3H), 1.23

(t, J = 7.1 Hz, 6H); ^{13}C NMR (100 MHz, $CDCl_3$) 13C NMR (101 MHz, CDCl3) δ 144.74, 139.73, 139.66 (d, J = 6.3 Hz), 134.84, 129.69, 128.75, 128.53, 127.94, 127.77, 119.22, 63.66 (dd, J = 5.7, 4.7 Hz), 59.55, 51.64 (d, J = 3.1 Hz), 21.66, 15.93 (dd, J = 7.4, 3.3 Hz); HRMS (ESI) calcd for $C_{21}H_{27}O_5NaPS_2^+$ (M+Na$^+$): 477.0935, found: 477.0932.

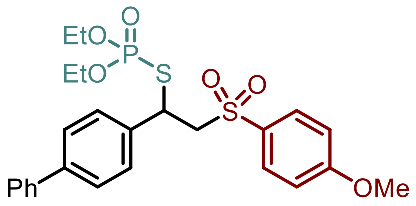

***S*-(1-([1,1'-Biphenyl]-4-yl)-2-((4-methoxyphenyl)sulfonyl)ethyl) *O,O*-diethyl phosphorothioate (5a):** purified by column chromatography (petroleum ether/ethyl acetate = 2:1), colorless oil, 88% yield. ^{1}H NMR (400 MHz, $CDCl_3$) δ 7.54 – 7.48 (m, 4H), 7.44 (t, J = 7.5 Hz, 2H), 7.41 – 7.33 (m, 3H), 7.23 (d, J = 8.3 Hz, 2H), 6.75 (d, J = 8.9 Hz, 2H), 4.78 (ddd, J = 12.5, 10.3, 4.7 Hz, 1H), 4.14 – 4.00 (m, 4H), 3.99 – 3.84 (m, 2H), 3.68 (s, 3H), 1.30 (t, J = 7.1 Hz, 3H), 1.21 (t, J = 7.1 Hz, 3H); ^{13}C NMR (100 MHz, $CDCl_3$) δ 163.51, 141.13, 140.19, 137.25 (d, J = 5.1 Hz), 130.77, 130.14, 128.90, 128.25, 127.67, 127.25, 126.93, 114.14, 63.98 (dd, J = 16.0, 6.1 Hz), 62.07 (d, J = 5.9 Hz), 55.52, 44.15 (d, J = 3.5 Hz), 15.93 (dd, J = 14.7, 7.4 Hz); HRMS (ESI) calcd for $C_{25}H_{29}O_6PS_2Na^+$ (M+Na$^+$): 543.1041, found: 543.1047.

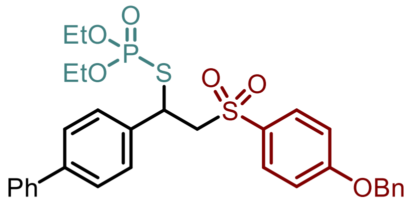

4-((2-([1,1'-Biphenyl]-4-yl)-2-((diethoxyphosphoryl)thio)ethyl)sulfonyl)phenyl benzoate (5b): purified by column chromatography (petroleum ether/ethyl acetate = 2:1), white solid, 65% yield. ^{1}H NMR (400 MHz, $CDCl_3$) δ 7.55 – 7.46 (m, 4H), 7.44 – 7.33 (m, 8H), 7.27 (dd, J = 7.9, 1.9 Hz, 2H), 7.21 (d, J = 8.2 Hz, 2H), 6.80 (d, J = 8.8 Hz, 2H), 4.94 – 4.74 (m, 3H), 4.15 – 3.83 (m, 6H), 1.30 (t, J = 7.1 Hz, 3H), 1.21 (t, J = 7.1 Hz, 3H); ^{13}C NMR (100 MHz, $CDCl_3$) δ 162.61, 141.14, 140.24, 137.15 (d, J = 5.2 Hz), 135.58, 130.98, 130.14, 128.96, 128.71, 128.40, 128.27, 127.70, 127.48, 127.25, 126.96, 114.89, 70.24, 64.01 (dd, J = 16.3, 6.1 Hz), 62.05 (d, J = 5.8 Hz), 44.18 (d, J =

3.4 Hz), 15.95 (dd, *J* = 14.8, 7.3 Hz); HRMS (ESI) calcd for $C_{31}H_{33}O_6PS_2Na^+$ ($M+Na^+$): 619.1354, found: 619.1354.

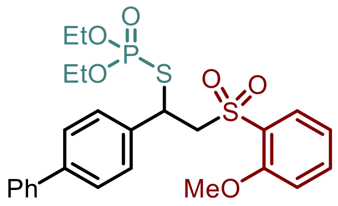

S-(1-([1,1'-Biphenyl]-4-yl)-2-((2-methoxyphenyl)sulfonyl)ethyl) *O,O*-diethyl phosphorothioate (5c): purified by column chromatography (petroleum ether/ethyl acetate = 2:1), white solid, 71% yield. ^1H NMR (400 MHz, $CDCl_3$) δ 7.56 (dd, *J* = 7.8, 1.2 Hz, 1H), 7.43 (d, *J* = 4.3 Hz, 4H), 7.38 – 7.32 (m, 2H), 7.28 – 7.22 (m, 2H), 7.17 (d, *J* = 8.2 Hz, 2H), 6.82 (t, *J* = 7.6 Hz, 1H), 6.75 (d, *J* = 8.3 Hz, 1H), 4.73 (td, *J* = 11.3, 4.1 Hz, 1H), 4.36 (dd, *J* = 14.5, 10.8 Hz, 1H), 4.17 – 3.98 (m, 4H), 3.95 – 3.75 (m, 4H), 1.30 (t, *J* = 7.1 Hz, 3H), 1.18 (t, *J* = 7.1 Hz, 3H); ^{13}C NMR (100 MHz, $CDCl_3$) δ 156.77, 141.01, 140.32, 137.35 (d, *J* = 4.9 Hz), 135.26, 130.41, 128.86, 128.10, 127.58, 126.93, 126.75, 120.49, 111.83, 63.93 (dd, *J* = 20.1, 6.0 Hz), 60.11 (d, *J* = 6.6 Hz), 56.20, 44.33 (d, *J* = 3.5 Hz), 15.92 (dd, *J* = 19.0, 7.4 Hz); HRMS (ESI) calcd for $C_{25}H_{29}O_6PS_2Na^+$ ($M+Na^+$): 543.1041, found: 543.1041.

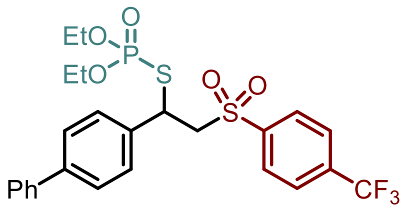

***S*-(1-([1,1'-Biphenyl]-4-yl)-2-((4-(trifluoromethyl)phenyl)sulfonyl)ethyl) *O,O*-diethyl phosphorothioate (5d):** purified by column chromatography (petroleum ether/ethyl acetate = 2:1), white solid, 71% yield. ^1H NMR (400 MHz, $CDCl_3$) δ 7.72 (d, *J* = 8.2 Hz, 2H), 7.57 (d, *J* = 8.3 Hz, 2H), 7.50 – 7.41 (m, 4H), 7.39 – 7.33 (m, 3H), 7.22 (d, *J* = 8.2 Hz, 2H), 4.82 (ddd, *J* = 12.9, 10.8, 4.4 Hz, 1H), 4.19 (dd, *J* = 14.8, 4.4 Hz, 1H), 4.13 – 3.99 (m, 4H), 3.98 – 3.86 (m, 1H), 1.30 (t, *J* = 7.1 Hz, 3H), 1.22 (t, *J* = 7.1 Hz, 3H); ^{13}C NMR (100 MHz, $CDCl_3$) δ 142.89, 141.61, 139.81, 136.62 (d, *J* = 5.6 Hz), 134.98 (q, *J* = 33.0 Hz), 128.91, 128.59, 128.25, 127.81, 127.34, 126.89, 126.00 (q, *J* = 3.6 Hz), 122.98 (q, *J* = 273.2 Hz), 64.16 (dd, *J* = 11.6, 6.3 Hz), 61.91 (d, *J* = 5.2 Hz), 43.88 (d, *J* = 3.4 Hz), 15.93 (dd, *J* = 11.7, 7.3 Hz); ^{19}F NMR (376 MHz, $CDCl_3$) δ

-63.19 (s); HRMS (ESI) calcd for $C_{25}H_{26}O_5F_3NaPS_2^+$ (M+Na$^+$): 581.0809, found: 581.0809.

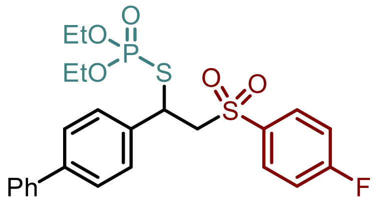

***S*-(1-([1,1'-Biphenyl]-4-yl)-2-((4-fluorophenyl)sulfonyl)ethyl) *O,O*-diethyl phosphorothioate (5e):** purified by column chromatography (petroleum ether/ethyl acetate = 2:1), white solid, 86% yield. ^{1}H NMR (400 MHz, $CDCl_3$) δ 7.65 – 7.57 (m, 2H), 7.50 (d, *J* = 7.4 Hz, 2H), 7.45 (t, *J* = 7.5 Hz, 2H), 7.41 – 7.34 (m, 3H), 7.24 (d, *J* = 8.2 Hz, 2H), 6.99 (t, *J* = 8.5 Hz, 2H), 4.80 (ddd, *J* = 12.6, 10.6, 4.6 Hz, 1H), 4.13 – 3.96 (m, 5H), 3.96 – 3.83 (m, 1H), 1.29 (t, *J* = 7.1 Hz, 3H), 1.22 (t, *J* = 7.1 Hz, 3H); ^{13}C NMR (100 MHz, $CDCl_3$) δ 165.56 (d, *J* = 256.5 Hz), 141.52, 140.12, 136.93 (d, *J* = 5.5 Hz), 135.39 (d, *J* = 3.1 Hz), 130.88 (d, *J* = 9.7 Hz), 128.94, 128.23, 127.74, 127.42, 126.99, 116.26 (d, *J* = 22.7 Hz), 64.09 (dd, *J* = 13.5, 6.2 Hz), 62.03 (d, *J* = 5.5 Hz), 44.02 (d, *J* = 3.5 Hz), 15.94 (dd, *J* = 12.2, 7.3 Hz); ^{19}F NMR (376 MHz, $CDCl_3$) δ -103.38 (tt, *J* = 8.3, 5.1 Hz); HRMS (ESI) calcd for $C_{24}H_{26}O_5FPS_2Na^+$ (M+Na$^+$): 531.0841, found: 531.0842.

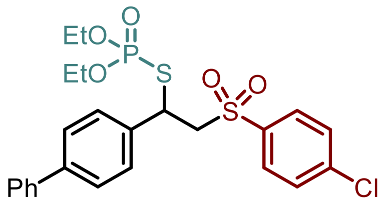

***S*-(1-([1,1'-Biphenyl]-4-yl)-2-((4-chlorophenyl)sulfonyl)ethyl) *O,O*-diethyl phosphorothioate (5f):** purified by column chromatography (petroleum ether/ethyl acetate = 2:1), colorless oil, 76% yield. ^{1}H NMR (400 MHz, $CDCl_3$) δ 7.55 – 7.48 (m, 4H), 7.45 (t, *J* = 7.5 Hz, 2H), 7.41 – 7.34 (m, 3H), 7.28 (d, *J* = 8.5 Hz, 2H), 7.22 (d, *J* = 8.2 Hz, 2H), 4.79 (ddd, *J* = 12.7, 10.6, 4.5 Hz, 1H), 4.15 – 3.96 (m, 5H), 3.95 – 3.85 (m, 1H), 1.30 (t, *J* = 7.1 Hz, 3H), 1.22 (t, *J* = 7.1 Hz, 3H); ^{13}C NMR (100 MHz, $CDCl_3$) δ 141.63, 140.15, 140.07, 137.82, 136.78 (d, *J* = 5.5 Hz), 129.44, 129.23, 128.91, 128.23, 127.76, 127.41, 127.08, 64.10 (dd, *J* = 13.2, 6.2 Hz), 61.96 (d, *J* = 5.5 Hz), 43.98 (d, *J*

= 3.5 Hz), 15.95 (dd, J = 12.9, 7.3 Hz); HRMS (ESI) calcd for $C_{24}H_{26}O_4ClPS_2Na^+$ (M+Na$^+$): 547.0545, found: 547.0546.

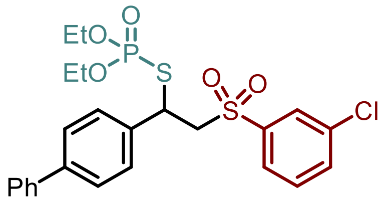

S-(1-([1,1'-Biphenyl]-4-yl)-2-((3-chlorophenyl)sulfonyl)ethyl) O,O-diethyl phosphorothioate (5g): purified by column chromatography (petroleum ether/ethyl acetate = 2:1), colorless solid, 54% yield. ^{1}H NMR (400 MHz, $CDCl_3$) δ 7.55 – 7.49 (m, 4H), 7.47 – 7.34 (m, 6H), 7.28 – 7.21 (m, 3H), 4.86 – 4.74 (m, 1H), 4.15 – 3.98 (m, 5H), 3.98 – 3.85 (m, 1H), 1.31 (t, J = 7.0 Hz, 3H), 1.22 (t, J = 7.0 Hz, 3H); ^{13}C NMR (100 MHz, $CDCl_3$) δ 141.46, 140.97, 140.10, 136.66 (d, J = 5.5 Hz), 135.12, 133.42, 130.31, 128.89, 128.28, 128.22, 127.72, 127.37, 127.00, 126.02, 64.10 (dd, J = 15.0, 6.2 Hz), 61.94 (d, J = 5.4 Hz), 44.00 (d, J = 3.4 Hz), 15.97 (dd, J = 14.1, 7.3 Hz); HRMS (ESI) calcd for HRMS (ESI) calcd for $C_{24}H_{26}O_4ClPS_2Na^+$ (M+Na$^+$): 547.0545, found: 547.0549.

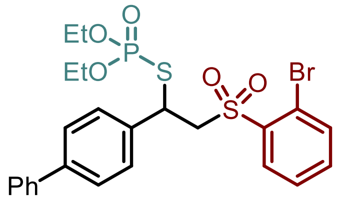

S-(1-([1,1'-Biphenyl]-4-yl)-2-((2-bromophenyl)sulfonyl)ethyl) O,O-diethyl phosphorothioate (5h): purified by column chromatography (petroleum ether/ethyl acetate = 2:1), colorless oil, 37% yield. ^{1}H NMR (400 MHz, $CDCl_3$) δ 7.61 (dd, J = 7.9, 1.6 Hz, 1H), 7.55 (dd, J = 7.9, 0.7 Hz, 1H), 7.47 – 7.40 (m, 4H), 7.39 – 7.33 (m, 1H), 7.31 – 7.16 (m, 5H), 7.15 – 7.06 (m, 1H), 4.82 (td, J = 11.5, 4.2 Hz, 1H), 4.62 (dd, J = 14.8, 11.1 Hz, 1H), 4.19 – 4.01 (m, 4H), 3.96 – 3.83 (m, 1H), 1.31 (t, J = 7.1 Hz, 3H), 1.23 (t, J = 7.1 Hz, 3H); ^{13}C NMR (100 MHz, $CDCl_3$) δ 141.21, 140.16, 138.32, 136.60 (d, J = 5.2 Hz), 134.84, 134.04, 132.07, 128.91, 128.15, 127.67, 127.17, 126.89, 120.58, 64.04 (dd, J = 16.1, 6.1 Hz), 59.22 (d, J = 6.2 Hz), 44.24 (d, J = 3.5 Hz), 15.97 (dd, J = 14.3, 7.3 Hz); HRMS (ESI) calcd for $C_{24}H_{26}O_5BrPS_2Na^+$ (M+Na$^+$): 591.0040, found: 591.0042.

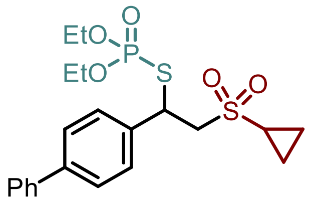

***S*-(1-([1,1'-Biphenyl]-4-yl)-2-(cyclopropylsulfonyl)ethyl) *O,O*-diethyl phosphorothioate (5i):** purified by column chromatography (petroleum ether/ethyl acetate = 2:1), colorless oil, 86% yield. ^{1}H NMR (400 MHz, $CDCl_3$) δ 7.64 – 7.56 (m, 4H), 7.52 (d, *J* = 8.3 Hz, 2H), 7.44 (t, *J* = 7.5 Hz, 2H), 7.36 (t, *J* = 7.3 Hz, 1H), 4.91 (ddd, *J* = 12.4, 9.8, 5.1 Hz, 1H), 4.20 – 4.04 (m, 3H), 3.97 – 3.81 (m, 3H), 1.94 (tt, *J* = 8.0, 4.8 Hz, 1H), 1.32 (t, *J* = 7.1 Hz, 3H), 1.22 (t, *J* = 7.0 Hz, 3H), 1.18 – 1.08 (m, 2H), 0.88 – 0.78 (m, 2H); ^{13}C NMR (100 MHz, $CDCl_3$) δ 141.51, 140.01, 138.26 (d, *J* = 5.1 Hz), 128.93, 128.36, 127.78, 127.60, 127.01, 64.09 (dd, *J* = 19.9, 6.1 Hz), 60.40 (d, *J* = 6.0 Hz), 44.05 (d, *J* = 3.4 Hz), 30.90, 15.95 (dd, *J* = 15.7, 7.4 Hz), 5.52, 5.22; HRMS (ESI) calcd for $C_{21}H_{27}O_5PS_2Na^+$ (M+Na$^+$): 477.0935, found: 477.0940.

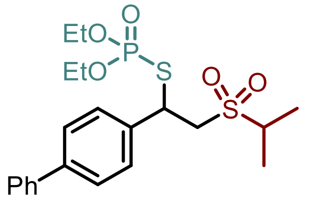

***S*-(1-([1,1'-Biphenyl]-4-yl)-2-(isopropylsulfonyl)ethyl) *O,O*-diethyl phosphorothioate (5j):** purified by column chromatography (petroleum ether/ethyl acetate = 2:1), colorless oil, 88% yield. ^{1}H NMR (400 MHz, $CDCl_3$) δ 7.65 – 7.54 (m, 4H), 7.50 (d, *J* = 8.2 Hz, 2H), 7.44 (t, *J* = 7.5 Hz, 2H), 7.36 (t, *J* = 7.3 Hz, 1H), 4.89 (ddd, *J* = 12.6, 9.6, 5.0 Hz, 1H), 4.27 – 4.01 (m, 3H), 3.98 – 3.85 (m, 2H), 3.75 (dd, *J* = 14.5, 9.6 Hz, 1H), 2.80 (dt, *J* = 13.7, 6.8 Hz, 1H), 1.38 – 1.30 (m, 6H), 1.27 (d, *J* = 6.8 Hz, 3H), 1.21 (t, *J* = 7.1 Hz, 3H); ^{13}C NMR (100 MHz, $CDCl_3$) δ 141.49, 140.11, 138.26 (d, *J* = 5.3 Hz), 128.90, 128.20, 127.72, 127.60, 127.04, 64.12 (dd, *J* = 24.2, 6.2 Hz), 55.82 (d, *J* = 5.7 Hz), 54.05, 43.71 (d, *J* = 3.4 Hz), 15.94 (dd, *J* = 18.7, 7.4 Hz), 15.32, 14.75; HRMS (ESI) calcd for $C_{21}H_{29}O_5PS_2Na^+$ (M+Na$^+$): 479.1092, found: 479.1087.

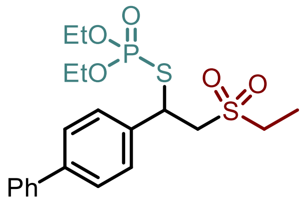

***S*-(1-([1,1'-Biphenyl]-4-yl)-2-(ethylsulfonyl)ethyl) *O,O*-diethyl phosphorothioate (5k):** purified by column chromatography (petroleum ether/ethyl acetate = 2:1), colorless oil, 92% yield. ^{1}H NMR (400 MHz, $CDCl_3$) δ 7.67 – 7.54 (m, 4H), 7.50 (d, *J* = 8.1 Hz, 2H), 7.45 (t, *J* = 7.5 Hz, 2H), 7.37 (t, *J* = 7.2 Hz, 1H), 4.93 – 4.77 (m, 1H), 4.20 – 4.04 (m, 3H), 3.96 – 3.84 (m, 2H), 3.76 (dd, *J* = 14.7, 9.8 Hz, 1H), 2.76 – 2.56 (m, 2H), 1.33 (t, *J* = 7.0 Hz, 3H), 1.23 (dt, *J* = 19.4, 7.2 Hz, 6H); ^{13}C NMR (100 MHz, $CDCl_3$) δ 141.62, 139.98, 138.03 (d, *J* = 5.2 Hz), 128.94, 128.24, 127.81, 127.71, 127.03, 64.14 (dd, *J* = 21.7, 6.1 Hz), 58.44 (d, *J* = 5.8 Hz), 48.61, 43.94 (d, *J* = 3.3 Hz), 15.94 (dd, *J* = 18.8, 7.4 Hz), 6.48; HRMS (ESI) calcd for $C_{20}H_{27}O_5PS_2Na^+$ ($M+Na^+$): 465.0935, found: 465.0934.

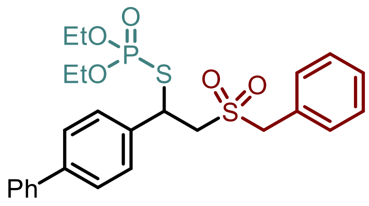

***S*-(1-([1,1'-Biphenyl]-4-yl)-2-(benzylsulfonyl)ethyl) *O,O*-diethyl phosphorothioate (5l):** purified by column chromatography (petroleum ether/ethyl acetate = 2:1), colorless solid, 47% yield. ^{1}H NMR (400 MHz, $CDCl_3$) δ 7.63 (d, *J* = 8.1 Hz, 2H), 7.58 (d, *J* = 7.7 Hz, 2H), 7.52 – 7.42 (m, 4H), 7.40 – 7.34 (m, 4H), 7.30 – 7.22 (m, 2H), 4.89 (ddd, *J* = 12.7, 9.6, 5.0 Hz, 1H), 4.18 – 4.02 (m, 3H), 4.02 – 3.83 (m, 3H), 3.78 (dd, *J* = 14.7, 5.0 Hz, 1H), 3.62 (dd, *J* = 14.7, 9.5 Hz, 1H), 1.32 (t, *J* = 7.1 Hz, 3H), 1.20 (t, *J* = 7.1 Hz, 3H); ^{13}C NMR (100 MHz, $CDCl_3$) δ 141.70, 140.04, 138.18 (d, *J* = 4.8 Hz), 130.88, 129.12, 128.96, 128.93, 128.46, 127.80, 127.71, 127.15, 127.07, 64.10 (dd, *J* = 23.8, 6.1 Hz), 60.67, 58.27 (d, *J* = 6.4 Hz), 43.97 (d, *J* = 3.4 Hz), 15.93 (dd, *J* = 19.3, 7.4 Hz); HRMS (ESI) calcd for $C_{25}H_{29}O_5PS_2Na^+$ ($M+Na^+$): 527.1092, found: 527.1086.

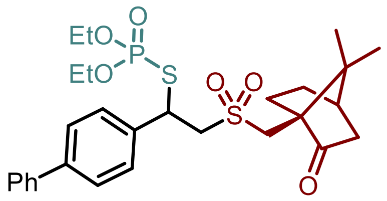

S-(1-([1,1'-Biphenyl]-4-yl)-2-((((1*S*,4*R*)-7,7-dimethyl-2-oxobicyclo[2.2.1]heptan-1-yl)methyl)sulfonyl)ethyl) *O,O*-diethyl phosphorothioate (5m): purified by column chromatography (petroleum ether/ethyl acetate = 2:1), colorless oil, 98% yield (dr= 1:1). ^{1}H NMR (400 MHz, $CDCl_3$) δ 7.61 – 7.50 (m, 6H), 7.43 (dd, *J* = 8.1, 6.8 Hz, 2H), 7.39 – 7.32 (m, 1H), 4.99 – 4.83 (m, 1H), 4.55 (dd, *J* = 14.7, 11.2 Hz, 0.5H), 4.24 (dd, *J* = 14.7, 5.9 Hz, 0.5H), 4.19 – 4.02 (m, 3H), 3.96 – 3.80 (m, 2H), 3.33 (d, *J* = 14.9 Hz, 0.5H), 2.75 (dd, *J* = 14.9, 9.8 Hz, 1H), 2.41 – 1.82 (m, 7H), 1.73 (ddd, *J* = 14.1, 9.3, 4.7 Hz, 0.5H), 1.45 – 1.36 (m, 1H), 1.32 (td, *J* = 7.0, 3.9 Hz, 3H), 1.23 (t, *J* = 7.1 Hz, 1.5H), 1.17 (t, *J* = 7.1 Hz, 1.5H), 1.00 (s, 1.5H), 0.83 (s, 1.5H), 0.80 (s, 1.5H), 0.46 (s, 1.5H); ^{13}C NMR (100 MHz, $CDCl_3$) δ 215.09 (d, *J* = 8.0 Hz), 141.51 (d, *J* = 28.7 Hz), 140.20 (d, *J* = 24.5 Hz), 138.39 (dd, *J* = 56.4, 4.6 Hz), 128.89 (d, *J* = 8.7 Hz), 128.60 (d, *J* = 56.9 Hz), 127.74 (d, *J* = 2.6 Hz), 127.61 (d, *J* = 2.6 Hz), 127.03 (d, *J* = 8.5 Hz), 64.06 (d, *J* = 6.0 Hz), 63.84 (dd, *J* = 9.4, 5.8 Hz), 62.23 (dd, *J* = 39.3, 6.7 Hz), 59.15 (d, *J* = 3.5 Hz), 52.54 (d, *J* = 30.7 Hz), 48.60 (d, *J* = 1.8 Hz), 44.19 (dd, *J* = 46.7, 3.4 Hz), 42.61 (d, *J* = 3.9 Hz), 42.45 (d, *J* = 24.4 Hz), 27.08 (d, *J* = 2.4 Hz), 25.56 (d, *J* = 16.5 Hz), 19.71 (d, *J* = 15.3 Hz), 19.41 (d, *J* = 10.5 Hz), 16.02 (d, *J* = 7.4 Hz), 15.83 (t, *J* = 7.8 Hz); HRMS (ESI) calcd for $C_{28}H_{37}O_6PS_2Na^+$ (M+Na$^+$): 587.1667, found: 587.1664.

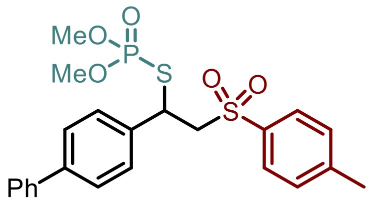

S-(1-([1,1'-biphenyl]-4-yl)-2-tosylethyl) *O,O*-dibenzyl phosphorothioate (5n): purified by column chromatography (petroleum ether/ethyl acetate = 2:1), white solid, 97% yield. ^{1}H NMR (400 MHz, $CDCl_3$) δ 7.56 – 7.48 (m, 4H), 7.48 – 7.34 (m, 5H), 7.25 (d, *J* = 8.2 Hz, 2H), 7.13 (d, *J* = 7.8 Hz, 2H), 4.84 – 4.71 (m, 1H), 4.06 – 3.89 (m, 2H), 3.70 (d, *J* = 12.8 Hz, 3H), 3.58 (d, *J* = 12.8 Hz, 3H), 2.31 (s, 3H); ^{13}C NMR (100 MHz, $CDCl_3$) δ 144.56, 141.23, 140.11, 137.21 (d, *J* = 4.9 Hz), 136.28, 129.65, 128.89, 128.22, 128.00, 127.71, 127.28, 126.95, 61.88 (d, *J* = 6.2 Hz), 54.01 (dd, *J* = 21.7, 5.9

Hz), 44.04 (d, J = 3.4 Hz), 21.58; HRMS (ESI) calcd for $C_{23}H_{25}O_5PNaS_2^+$ (M+Na$^+$): 499.0779, found: 499.0777.

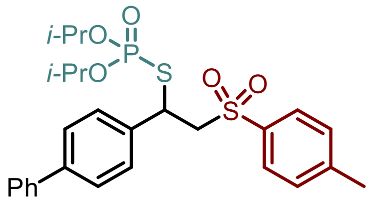

***S*-(1-([1,1'-biphenyl]-4-yl)-2-tosylethyl) *O,O*-diisopropyl phosphorothioate (5o):** purified by column chromatography (petroleum ether/ethyl acetate = 2:1), colorless oil, 57% yield. ^{1}H NMR (400 MHz, $CDCl_3$) δ 7.55 – 7.40 (m, 6H), 7.39 – 7.33 (m, 3H), 7.23 (d, J = 8.2 Hz, 2H), 7.10 (d, J = 8.0 Hz, 2H), 4.80 (td, J = 12.2, 4.0 Hz, 1H), 4.71 – 4.54 (m, 2H), 4.15 (dd, J = 14.6, 4.1 Hz, 1H), 3.99 (dd, J = 14.5, 11.0 Hz, 1H), 2.29 (s, 3H), 1.35 – 1.26 (m, 9H), 1.22 (d, J = 6.2 Hz, 3H); ^{13}C NMR (100 MHz, $CDCl_3$) δ 144.33, 141.17, 140.32, 137.09 (d, J = 6.3 Hz), 136.45, 129.55, 128.86, 128.27, 127.98, 127.61, 127.31, 126.97, 73.43 (dd, J = 10.5, 6.8 Hz), 61.96 (d, J = 4.7 Hz), 44.18 (d, J = 3.5 Hz), 23.81 (t, J = 3.8 Hz), 23.56 (dd, J = 14.1, 5.6 Hz), 21.54; HRMS (ESI) calcd for $C_{27}H_{33}O_5PS_2Na^+$ (M+Na$^+$): 555.1405, found: 555.1406.

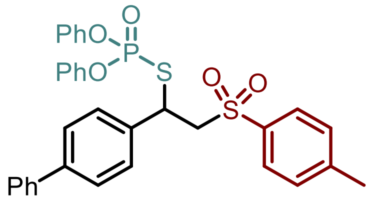

***S*-(1-([1,1'-biphenyl]-4-yl)-2-tosylethyl) *O,O*-diphenyl phosphorothioate (5p):** purified by column chromatography (petroleum ether/ethyl acetate = 2:1), white solid, 70% yield. ^{1}H NMR (400 MHz, $CDCl_3$) δ 7.51 – 7.41 (m, 6H), 7.38 – 7.27 (m, 7H), 7.23 – 7.01 (m, 10H), 4.95 – 4.80 (m, 1H), 4.01 – 3.86 (m, 2H), 2.28 (s, 3H); ^{13}C NMR (100 MHz, $CDCl_3$) δ 149.88 (t, J = 7.9 Hz), 144.50, 141.42, 140.20, 136.18, 136.12, 129.91, 129.61, 128.89, 128.26, 128.03, 127.70, 127.38, 126.99, 125.86 (dd, J = 6.4, 1.3 Hz), 120.66 (dd, J = 4.9, 1.3 Hz), 61.62 (d, J = 5.1 Hz), 45.27 (d, J = 3.6 Hz), 21.59; HRMS (ESI) calcd for $C_{33}H_{29}O_5PS_2Na^+$ (M+Na$^+$): 623.1092, found: 623.1089.

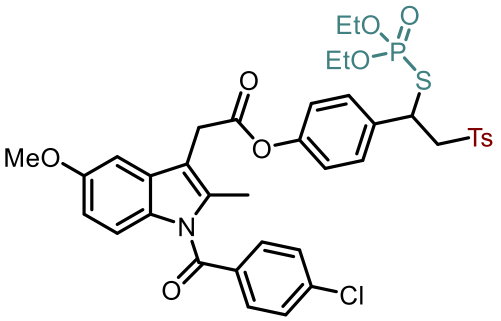

4-(1-((Diethoxyphosphoryl)thio)-2-tosylethyl)phenyl 2-(1-(4-chlorobenzoyl)-5-methoxy-2-methyl-1H-indol-3-yl)acetate (6a): purified by column chromatography (petroleum ether/ethyl acetate = 2:1), yellow oil, 94% yield; ^{1}H NMR (400 MHz, $CDCl_3$) δ 7.68 (d, *J* = 8.4 Hz, 2H), 7.55 – 7.44 (m, 4H), 7.22 – 7.13 (m, 4H), 7.04 (d, *J* = 2.3 Hz, 1H), 6.93 – 6.85 (m, 3H), 6.70 (dd, *J* = 9.0, 2.4 Hz, 1H), 4.73 (ddd, *J* = 12.8, 10.1, 4.7 Hz, 1H), 4.08 – 3.94 (m, 4H), 3.93 – 3.76 (m, 7H), 2.45 (s, 3H), 2.31 (s, 3H), 1.27 (t, *J* = 7.0 Hz, 3H), 1.20 (t, *J* = 7.1 Hz, 3H); ^{13}C NMR (100 MHz, $CDCl_3$) δ 168.87, 168.31, 156.12, 150.47, 144.78, 139.40, 136.32, 136.27, 136.16, 133.76, 131.22, 130.85, 130.46, 129.80, 129.19, 128.86, 127.91, 121.68, 115.04, 111.84, 111.65, 101.35, 64.03 (dd, *J* = 11.1, 6.1 Hz), 61.89 (d, *J* = 6.1 Hz), 55.74, 43.60 (d, *J* = 3.3 Hz), 30.52, 21.48, 15.92 (dd, *J* = 9.7, 7.3 Hz), 13.44; HRMS (ESI) calcd for $C_{38}H_{39}NO_9S_2NaPCl^+$ (M+Na$^+$): 806.1390, found: 806.1386.

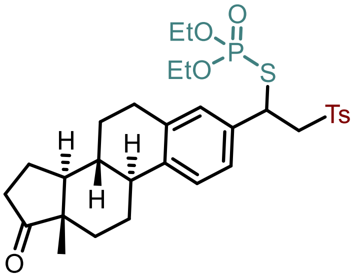

***O,O*-Diethyl *S*-(1-((8*R*,9*S*,13*S*,14*S*)-13-methyl-17-*oxo*-7,8,9,11,12,13,14,15,16,17-decahydro-6*H*-cyclopenta[*a*]phenanthren-3-yl)-2-tosylethyl) phosphorothioate (6b)**: purified by column chromatography (petroleum ether/ethyl acetate = 2:1), color-less oil, 55% yield; ^{1}H NMR (400 MHz, $CDCl_3$) δ 7.49 (dd, *J* = 8.1, 6.0 Hz, 2H), 7.19 – 7.09 (m, 3H), 6.98 (td, *J* = 7.9, 1.6 Hz, 1H), 6.85 – 6.80 (m, 1H), 4.73 – 4.61 (m, 1H), 4.15 – 3.99 (m, 4H), 3.96 – 3.86 (m, 2H), 2.92 – 2.57 (m, 2H), 2.52 (dd, *J* = 18.8, 8.6 Hz, 1H), 2.42 – 2.33 (m, 4H), 2.23 – 1.95 (m, 5H), 1.71 – 1.45 (m, 5H), 1.44 – 1.27 (m, 5H), 1.23 (t, *J* = 7.1 Hz, 3H), 0.92 (d, *J* = 6.2 Hz, 3H); ^{13}C NMR (100 MHz, $CDCl_3$) δ

144.15 (d, J = 3.2 Hz), 139.96, 136.76 (d, J = 3.1 Hz), 136.55 (d, J = 2.4 Hz), 135.34 (t, J = 6.2 Hz), 129.41 (d, J = 2.0 Hz), 128.04, 127.96, 127.94, 125.72 (d, J = 2.5 Hz), 125.15 (d, J = 5.1 Hz), 63.94 (dd, J = 19.6, 6.0 Hz), 61.85 (t, J = 4.6 Hz), 50.42 (d, J = 2.1 Hz), 47.90 (d, J = 1.4 Hz), 44.34, 43.97 (dd, J = 5.3, 3.7 Hz), 38.04 (d, J = 6.9 Hz), 35.83, 31.51, 29.19, 26.37 (d, J = 3.2 Hz), 25.67 (d, J = 6.4 Hz), 21.69 (d, J = 5.5 Hz), 21.58 (d, J = 1.6 Hz), 15.95 (dd, J = 15.5, 7.4 Hz), 13.87 (d, J = 2.9 Hz); HRMS (ESI) calcd for $C_{31}H_{41}O_6S_2PNa^+$ (M+Na$^+$): 627.1980, found: 627.1966.

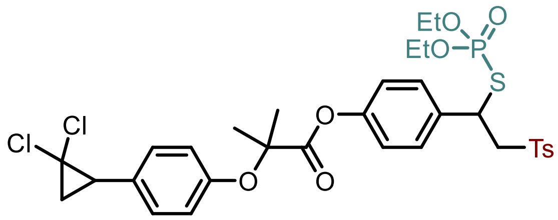

4-(1-((Diethoxyphosphoryl)thio)-2-tosylethyl)phenyl 2-(4-(2,2-dichlorocyclopropyl)phenoxy)-2-methylpropanoate (6c): purified by column chromatography (petroleum ether/ethyl acetate = 2:1), colorless oil, 78% yield; ^{1}H NMR (400 MHz, $CDCl_3$) δ 7.49 (d, J = 8.3 Hz, 2H), 7.20 – 7.15 (m, 6H), 6.93 – 6.89 (m, 2H), 6.79 (d, J = 8.4 Hz, 2H), 4.73 (ddd, J = 12.8, 10.0, 4.8 Hz, 1H), 4.09 – 3.95 (m, 4H), 3.90 – 3.80 (m, 2H), 2.86 (dd, J = 10.6, 8.4 Hz, 1H), 2.33 (s, 3H), 1.97 (dd, J = 10.7, 7.4 Hz, 1H), 1.84 – 1.78 (m, 1H), 1.74 (s, 6H), 1.28 (t, J = 7.1 Hz, 3H), 1.20 (t, J = 7.1 Hz, 3H); ^{13}C NMR (100 MHz, $CDCl_3$) δ 172.51, 154.92, 150.28, 144.77, 136.48 (d, J = 4.8 Hz), 136.11, 129.84, 129.79, 128.93, 128.49, 127.91, 121.54, 118.42, 79.22, 64.04 (dd, J = 11.6, 6.1 Hz), 61.88 (d, J = 6.2 Hz), 60.90, 43.59 (d, J = 3.4 Hz), 34.78, 25.83, 25.48 (t, J = 2.5 Hz), 21.55, 15.93 (dd, J = 10.4, 7.3 Hz); HRMS (ESI) calcd for $C_{32}H_{37}O_8S_2PNaCl_2^+$ (M+Na$^+$): 737.0942, found: 737.0954.

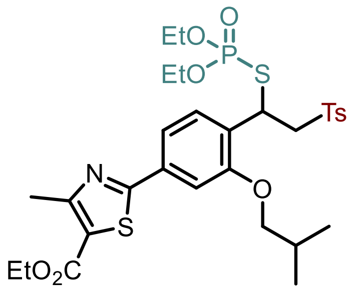

Ethyl 2-(4-(1-((diethoxyphosphoryl)thio)-2-tosylethyl)-3-isobutoxyphenyl)-4-methylthiazole-5-carboxylate (6d): purified by column chromatography (petroleum

ether/ethyl acetate = 2:1), colorless oil, 71% yield; ^{1}H NMR (400 MHz, $CDCl_3$) δ 7.77 (dd, *J* = 8.6, 2.2 Hz, 1H), 7.66 (d, *J* = 2.2 Hz, 1H), 7.43 (d, *J* = 8.2 Hz, 2H), 7.07 (d, *J* = 8.1 Hz, 2H), 6.63 (d, *J* = 8.7 Hz, 1H), 4.85 (s, 1H), 4.41 – 4.29 (m, 3H), 4.19 – 4.03 (m, 3H), 3.96 – 3.85 (m, 2H), 3.67 (dt, *J* = 14.6, 8.6 Hz, 2H), 2.78 (s, 3H), 2.30 (s, 3H), 2.13 (dd, *J* = 13.2, 6.6 Hz, 1H), 1.41 (t, *J* = 7.1 Hz, 3H), 1.34 (t, *J* = 7.1 Hz, 3H), 1.23 (t, *J* = 7.1 Hz, 3H), 1.10 (t, *J* = 6.7 Hz, 6H); ^{13}C NMR (100 MHz, $CDCl_3$) δ 168.73, 162.30, 161.08, 158.56, 144.34, 135.92, 129.36, 128.75, 128.18, 127.89, 126.24 (d, *J* = 4.6 Hz), 125.39, 121.09, 111.67, 74.98, 63.89 (dd, *J* = 16.3, 6.0 Hz), 61.24, 60.01 (d, *J* = 3.9 Hz), 28.31, 21.52, 19.57, 19.48, 17.52, 15.96 (dd, *J* = 17.4, 7.3 Hz), 14.38; HRMS (ESI) calcd for $C_{30}H_{40}NO_8NaPS_3^+$ (M+Na$^+$): 692.1551, found: 692.1546.

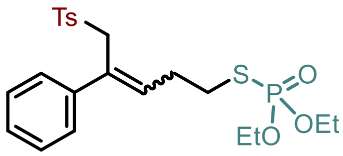

***O,O*-Diethyl *S*-(4-phenyl-5-tosylpent-3-en-1-yl) phosphorothioate (8)**: purified by column chromatography (petroleum ether/ethyl acetate = 2:1), colorless oil, 24% yield; ^{1}H NMR (400 MHz, $CDCl_3$) δ 7.64 (d, *J* = 7.9 Hz, 2H), 7.28 – 7.20 (m, 5H), 7.04 (d, *J* = 6.6 Hz, 2H), 5.65 (t, *J* = 7.1 Hz, 1H), 4.16 – 4.00 (m, 6H), 2.75 (dt, *J* = 14.5, 7.2 Hz, 2H), 2.40 (d, *J* = 6.8 Hz, 5H), 1.29 (t, *J* = 6.9 Hz, 6H); ^{13}C NMR (100 MHz, $CDCl_3$) δ 144.58, 137.86, 135.79, 134.68, 130.90, 129.61, 128.45, 128.29, 127.51, 64.89, 63.54 (d, *J* = 5.9 Hz), 30.67 (d, *J* = 5.3 Hz), 30.14 (d, *J* = 3.8 Hz), 21.64, 16.06 (d, *J* = 7.3 Hz). HRMS (ESI) calcd for $C_{22}H_{29}O_5NaPS_2^+$ (M+Na$^+$): 491.1092, found: 491.1092.

2. Scale-Up Experiment

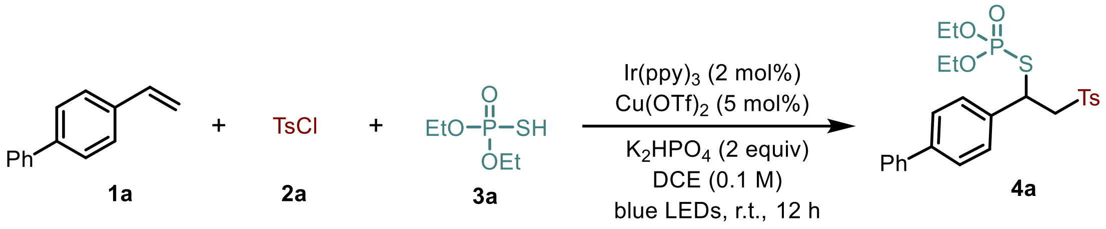

An oven-dried flask was charged with 4-vinyl-1,1'-biphenyl **1a** (6 mmol, 1.0 equiv), 4-methylbenzenesulfonyl chloride **2a** (12 mmol, 2 equiv), K_2HPO_4 (12 mmol, 2 equiv), $Cu(OTf)_2$ (5 mol%) and *fac*-Ir(ppy)$_3$ (2 mol%) under nitrogen atmosphere. Then anhydrous DCE (60 mL) and *O*,*O*-diethyl *S*-hydrogen phosphorothioate **3a** (6 mmol, 2 equiv) were added to the flask. The mixture was placed around a 30 W blue LEDs at a distance of ~5 cm and stirred under blue light irradiation for 12 hours at room temperature. After completion of reaction as monitored by TLC analysis, the mixture was filtered through a celite pad, and washed with ethyl acetate, then the filtrate was evaporated and the residue was purified directly by flash column chromatography on silica gel (petroleum ether/ethyl acetate = 2:1) to give the corresponding product **4a** in 78% yield (2.342 g).

3. Copies of ^{1}H ,^{13}C NMR and ^{19}F NMR Spectra

^{1}H NMR spectrum of compound **4a** (400 MHz, $CDCl_3$)

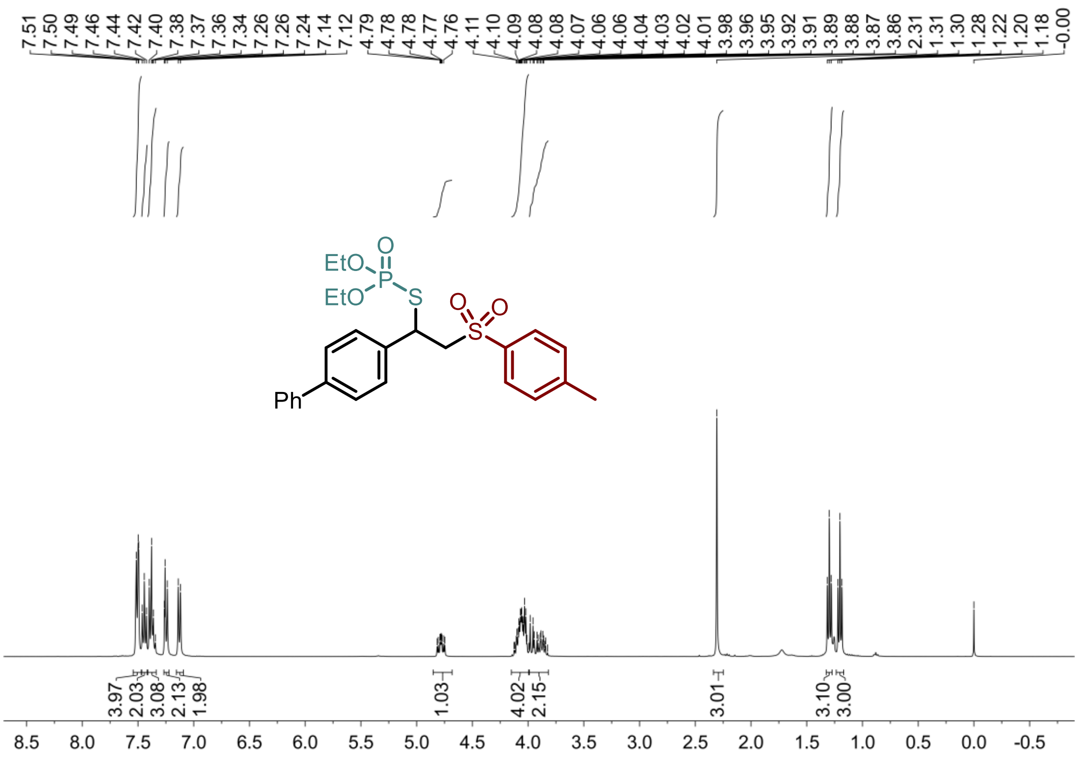

^{13}C NMR spectrum of compound **4a** (100 MHz, $CDCl_3$)

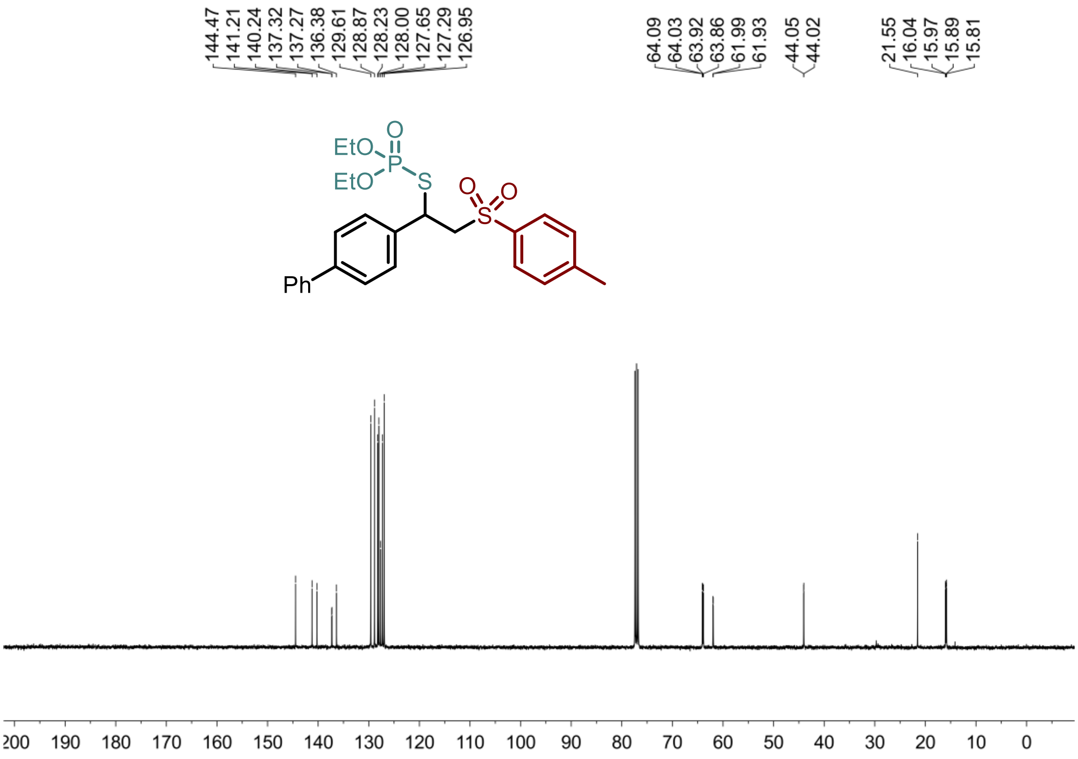

^{1}H NMR spectrum of compound **4b** (400 MHz, $CDCl_3$)

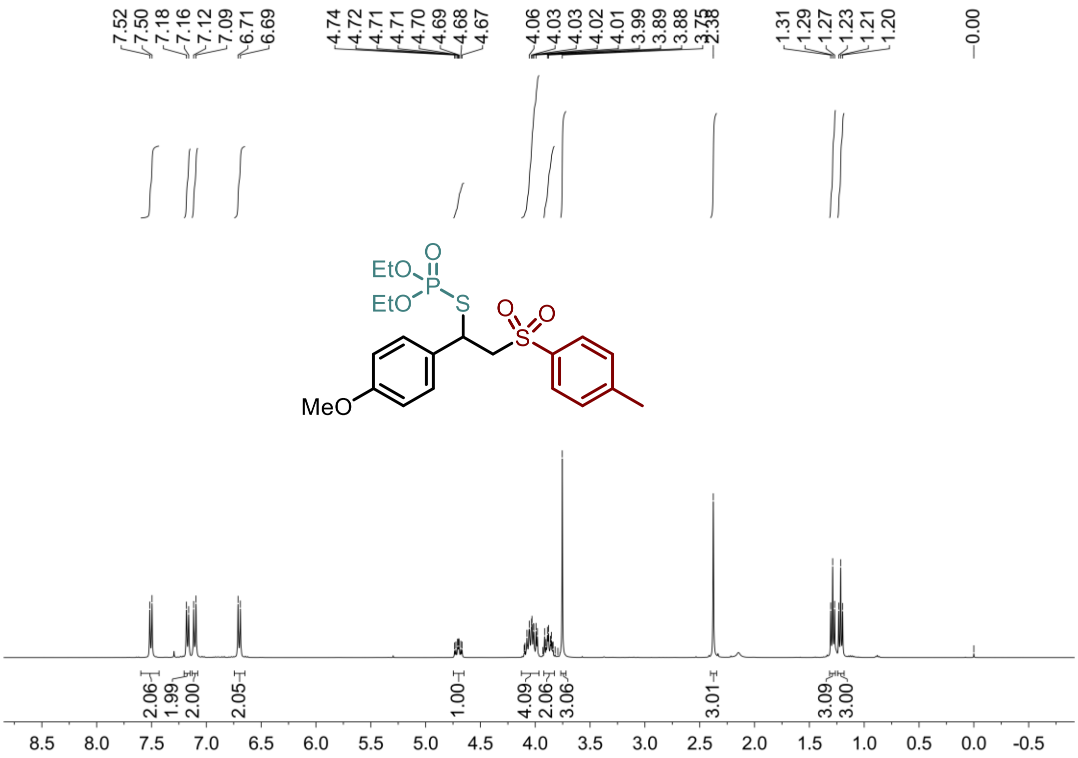

^{13}C NMR spectrum of compound **4b** (100 MHz, $CDCl_3$)

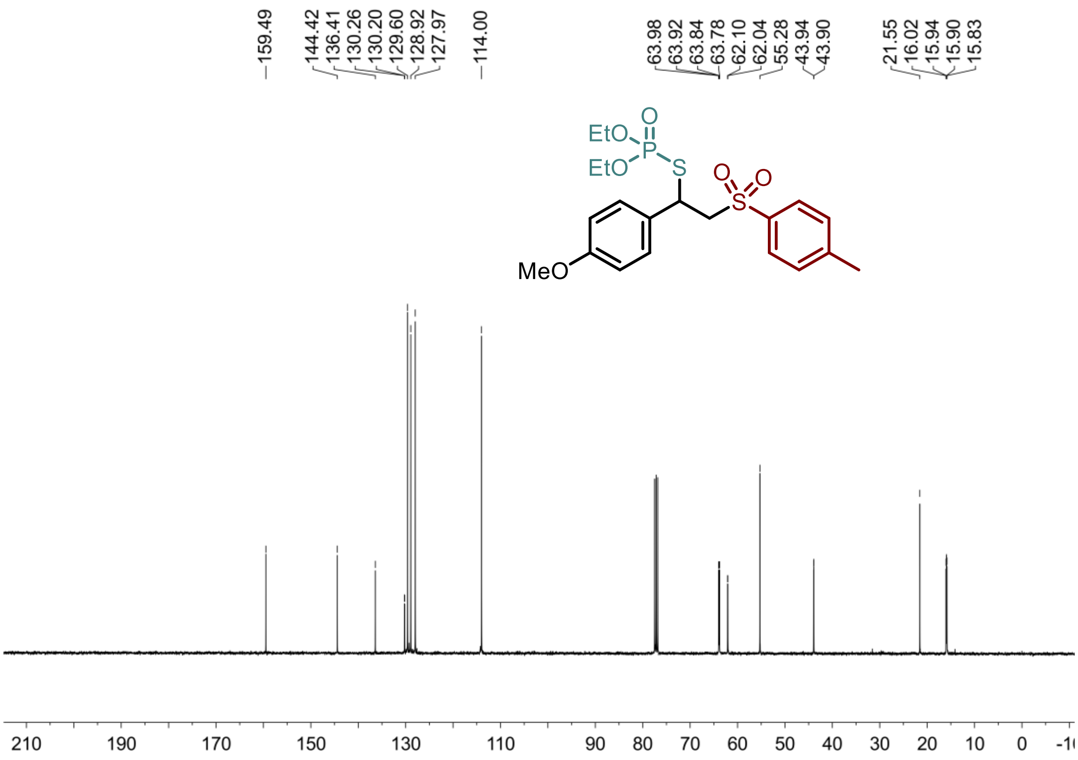

^{1}H NMR spectrum of compound **4c** (400 MHz, $CDCl_3$)

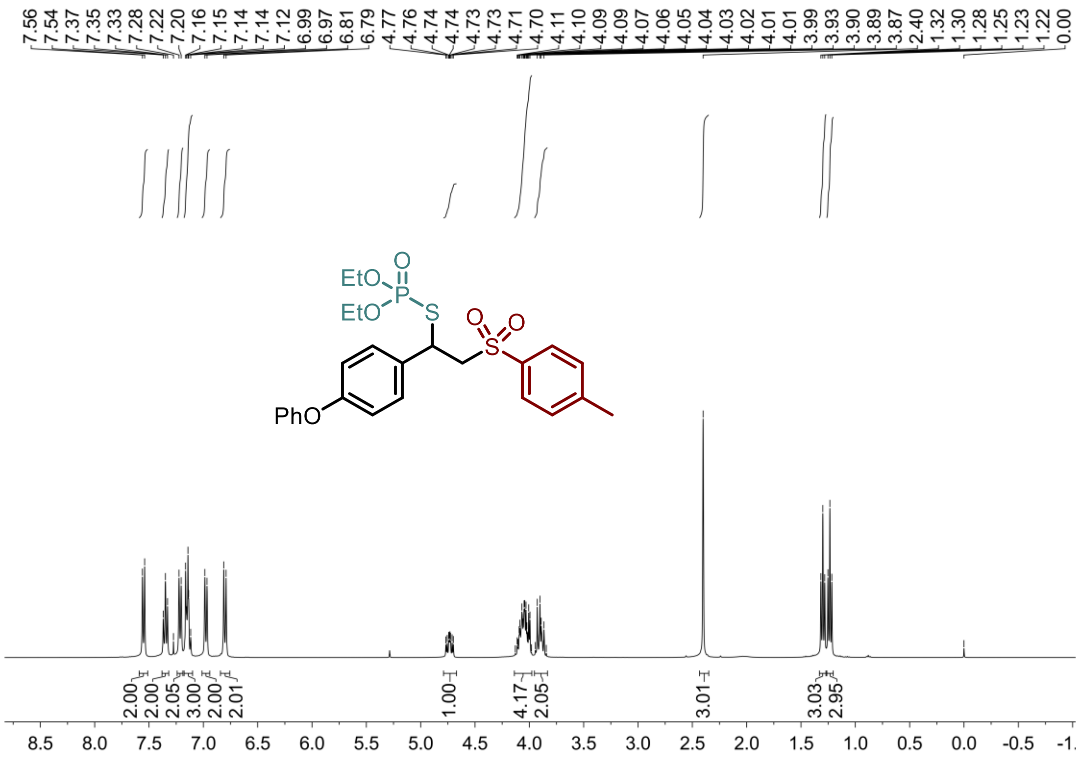

^{13}C NMR spectrum of compound **4c** (100 MHz, $CDCl_3$)

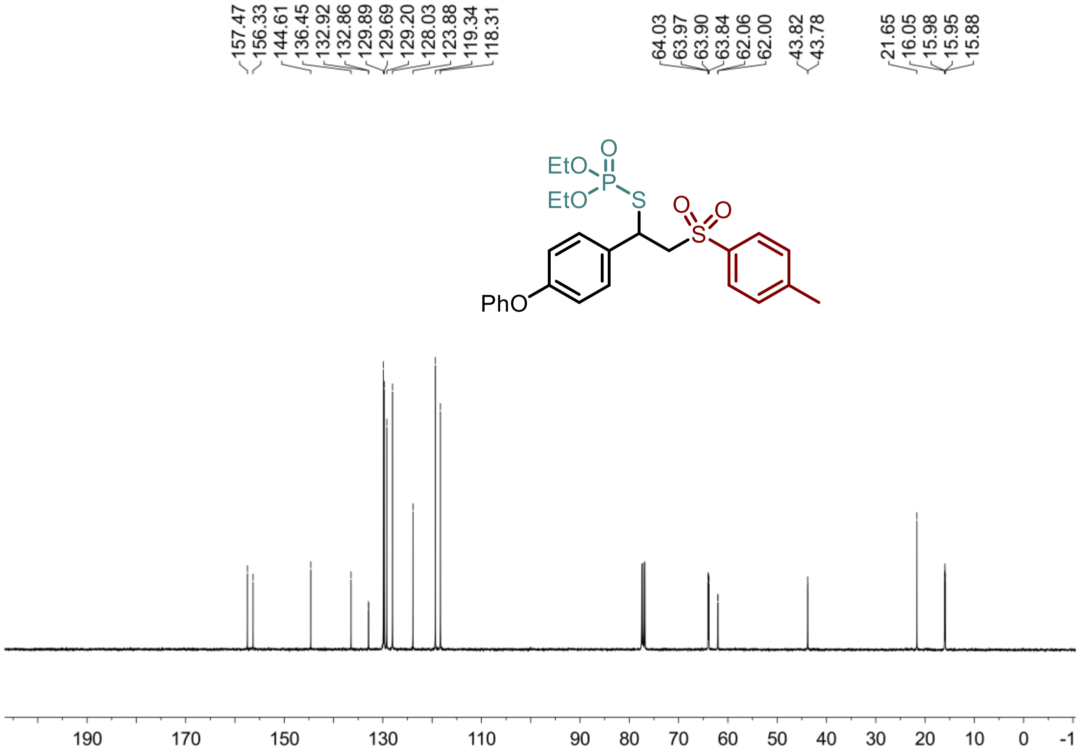

^{1}H NMR spectrum of compound **4d** (400 MHz, $CDCl_3$)

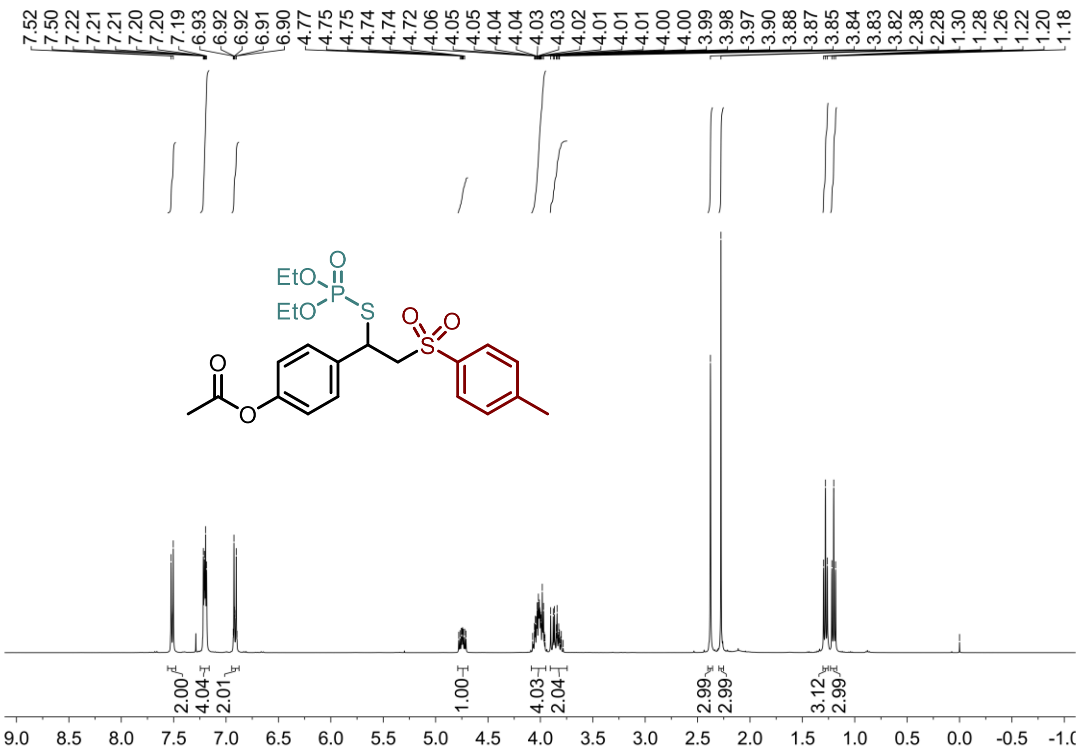

^{13}C NMR spectrum of compound **4d** (100 MHz, $CDCl_3$)

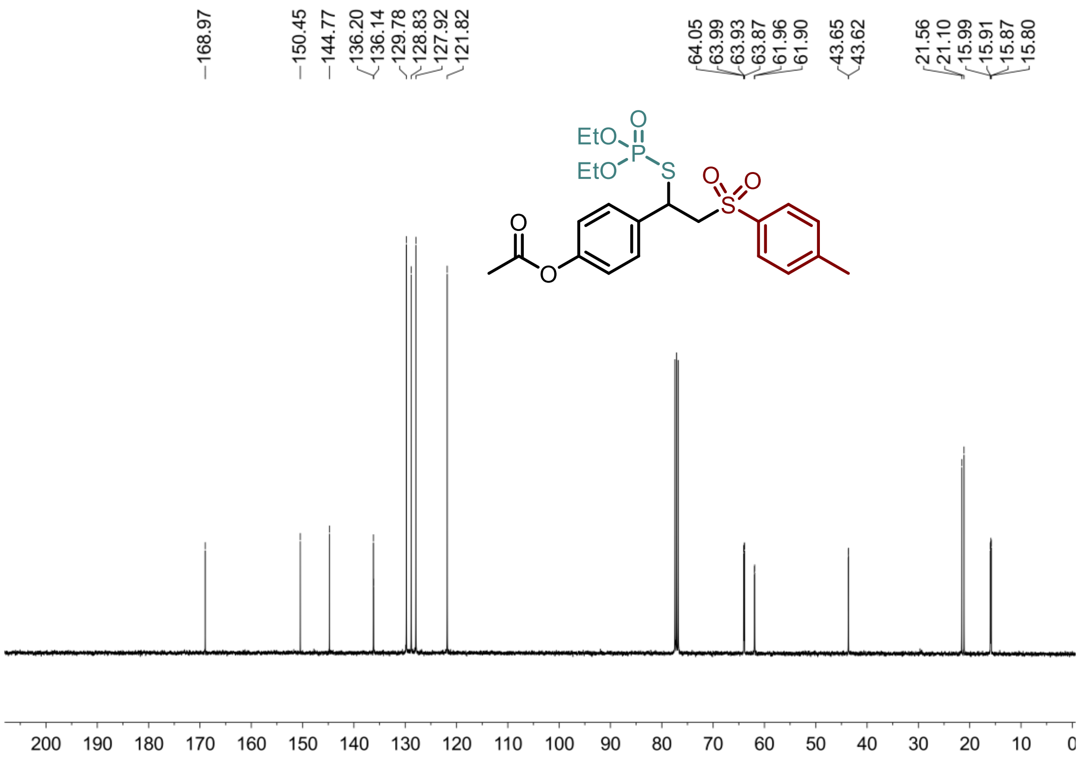

^{1}H NMR spectrum of compound **4e** (400 MHz, $CDCl_3$)

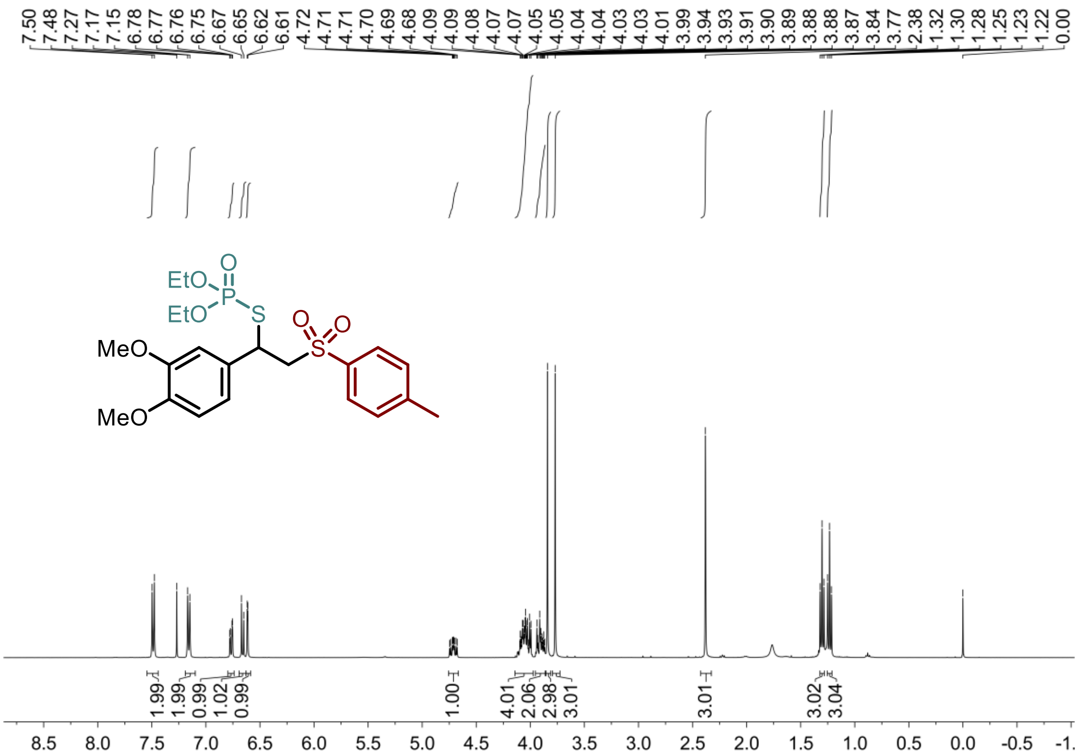

^{13}C NMR spectrum of compound **4e** (100 MHz, $CDCl_3$)

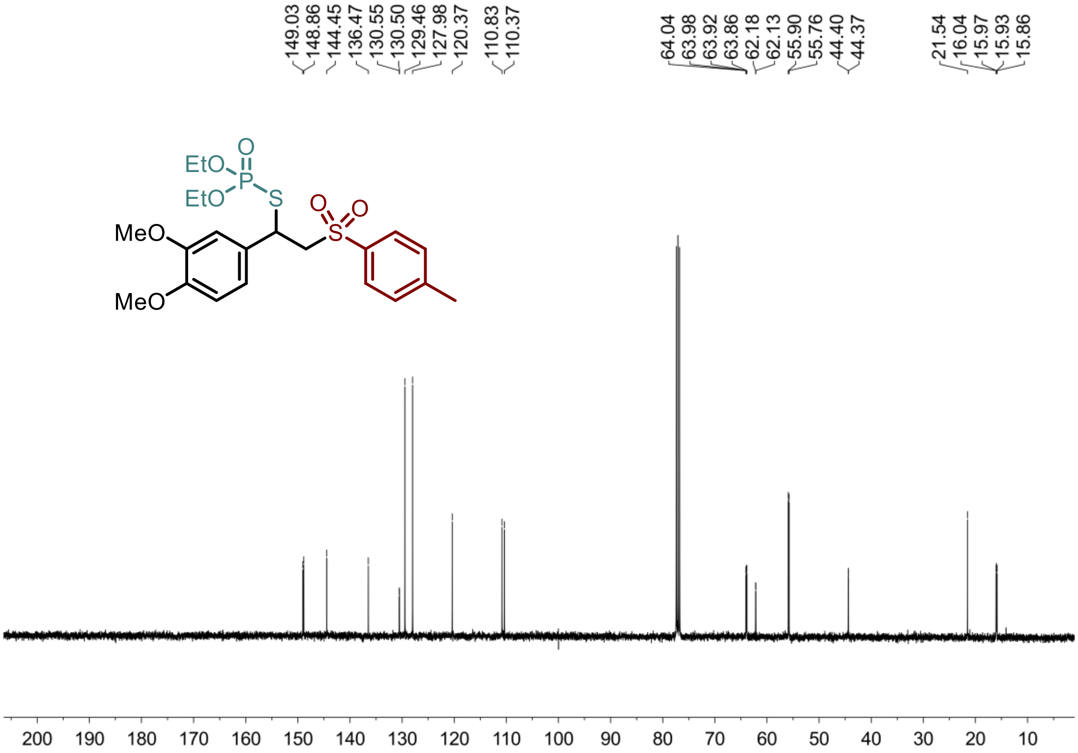

^{1}H NMR spectrum of compound **4f** (400 MHz, $CDCl_3$)

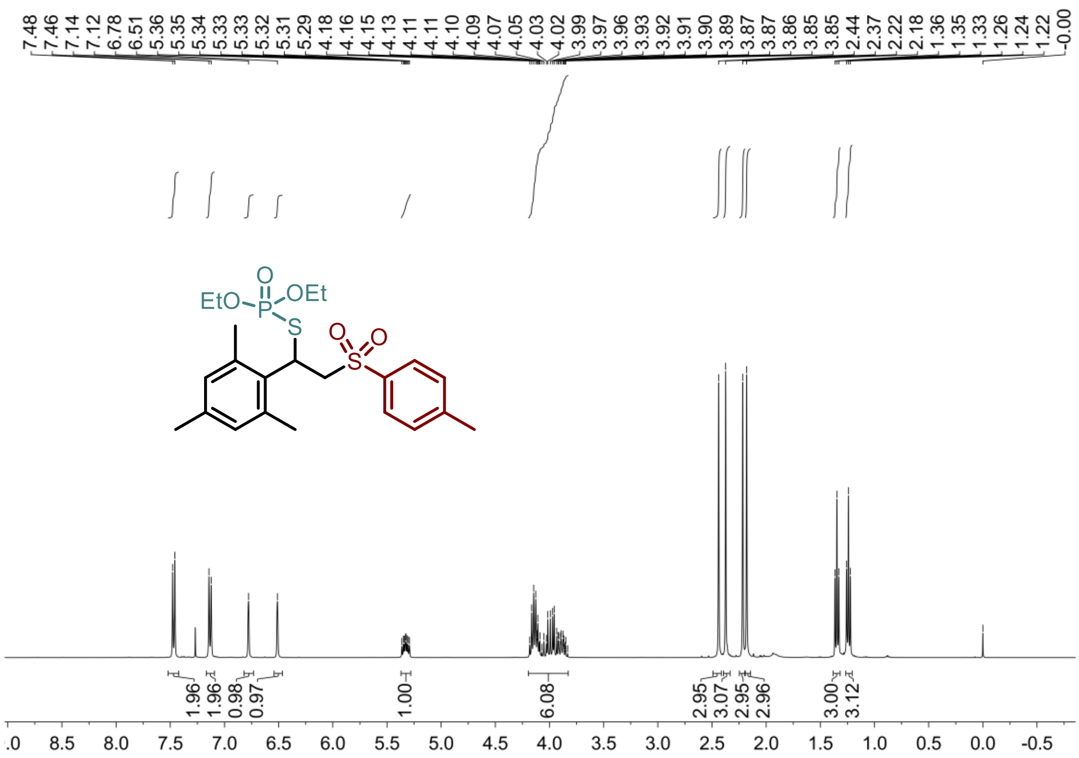

^{13}C NMR spectrum of compound **4f** (100 MHz, $CDCl_3$)

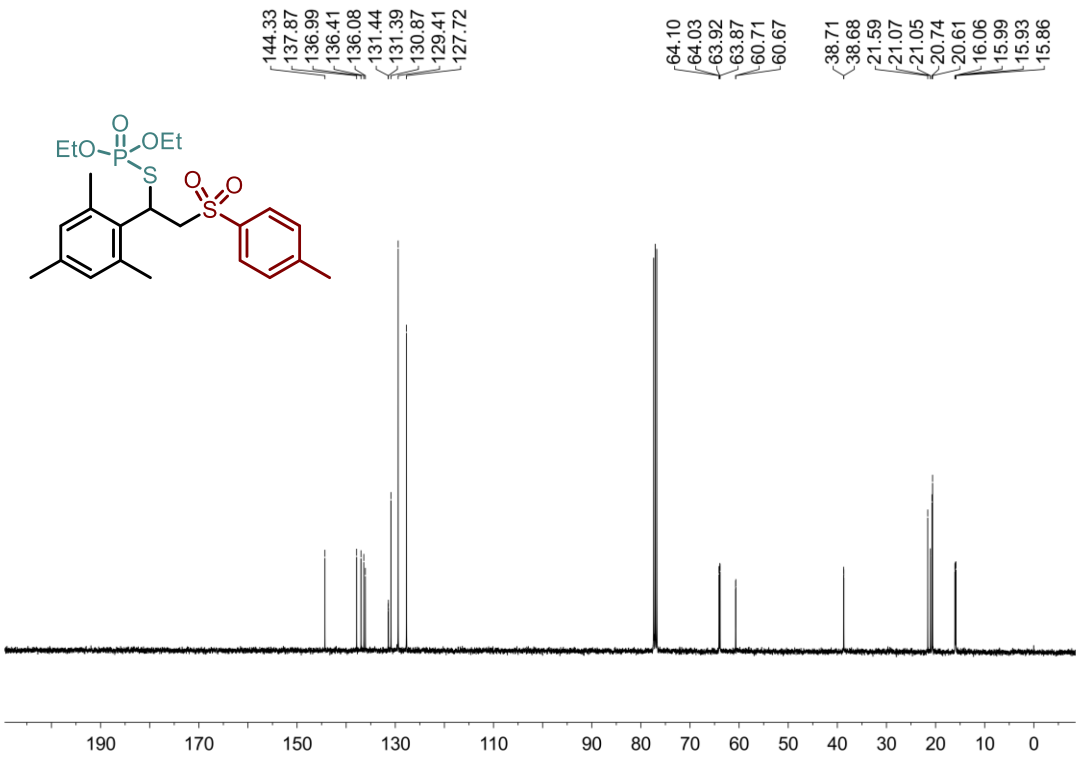

^{1}H NMR spectrum of compound **4g** (400 MHz, $CDCl_3$)

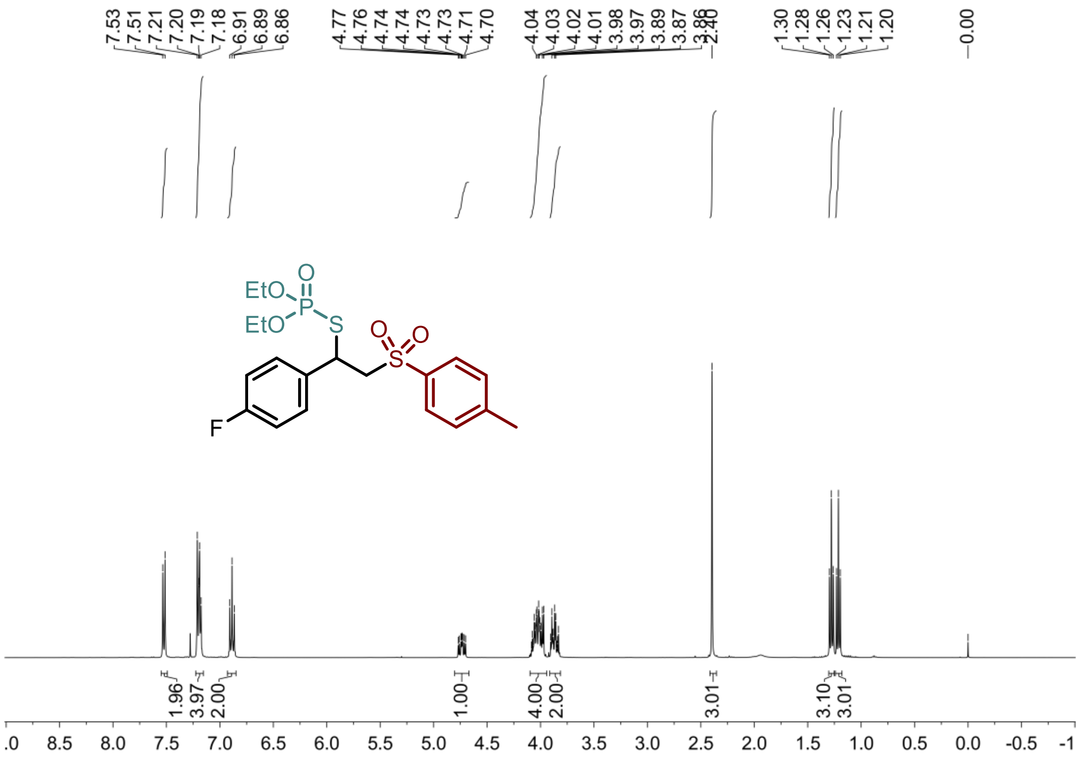

^{13}C NMR spectrum of compound **4g** (100 MHz, $CDCl_3$)

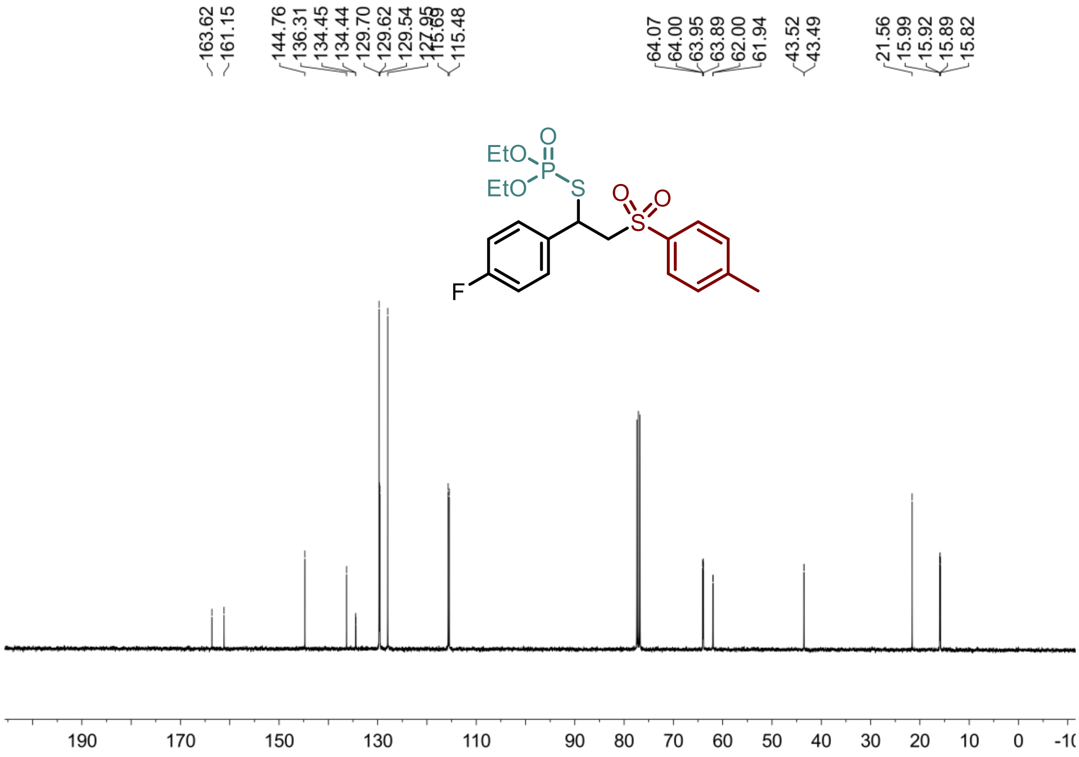

^{19}F NMR spectrum of compound **4g** (376 MHz, $CDCl_3$)

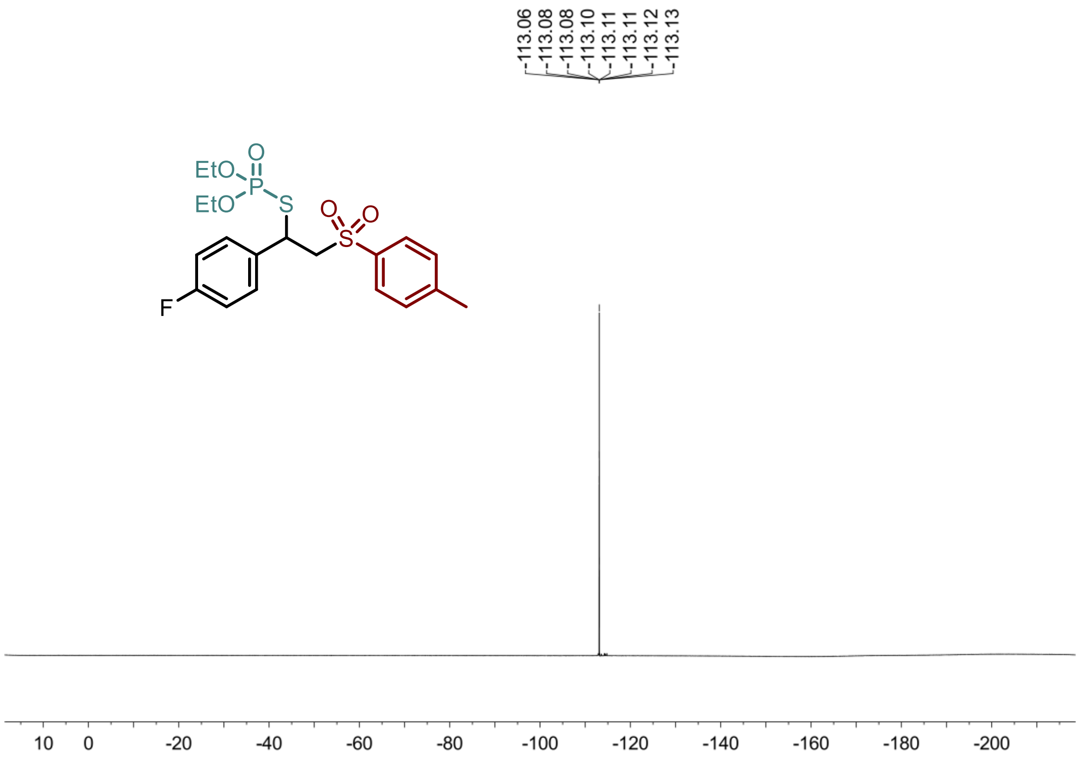

^{1}H NMR spectrum of compound **4h** (400 MHz, $CDCl_3$)

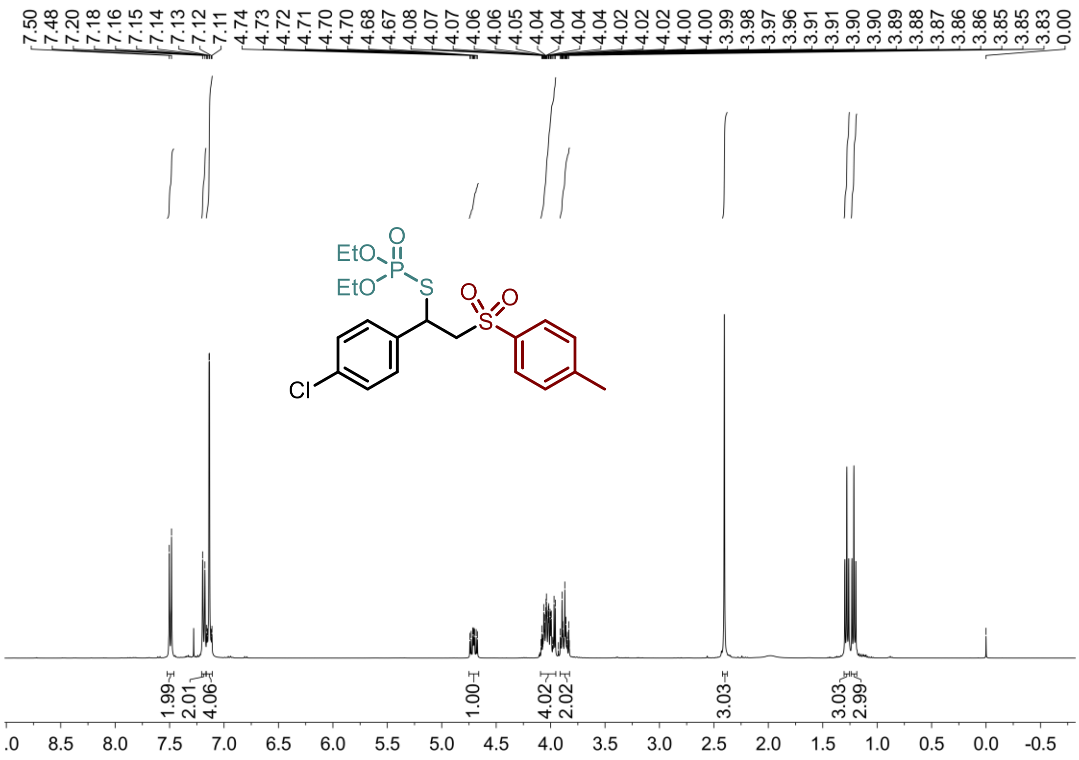

^{13}C NMR spectrum of compound **4h** (100 MHz, $CDCl_3$)

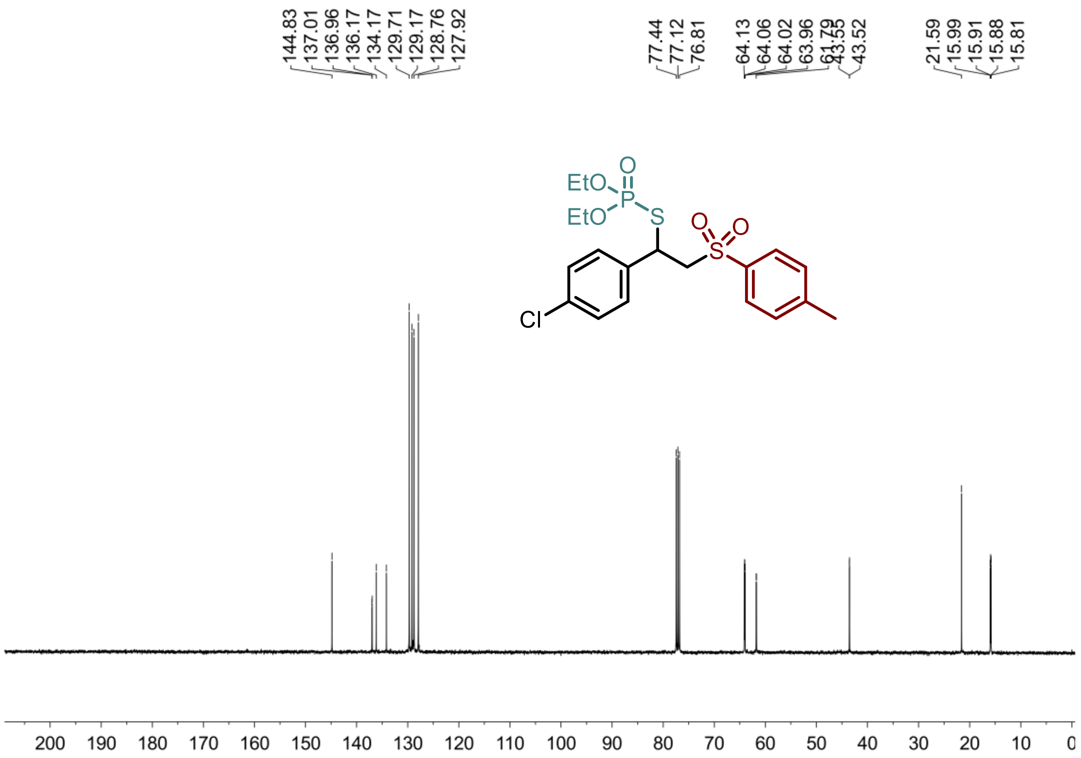

^{1}H NMR spectrum of compound **4i** (400 MHz, $CDCl_3$)

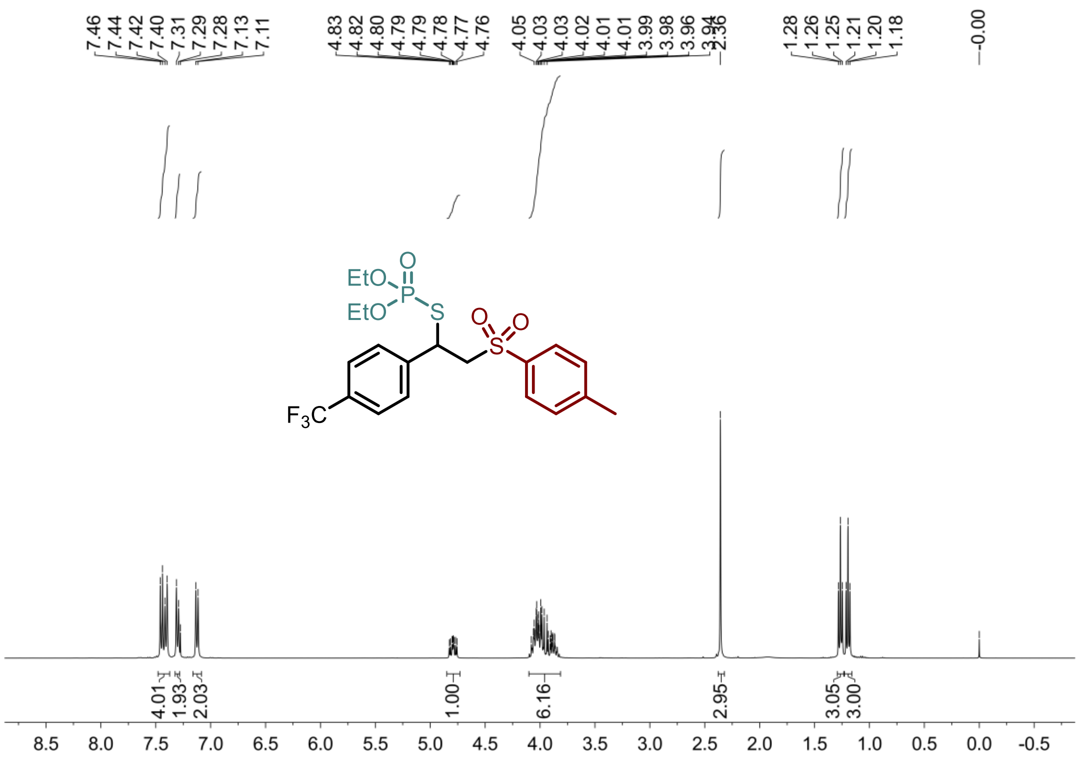

^{13}C NMR spectrum of compound **4i** (100 MHz, $CDCl_3$)

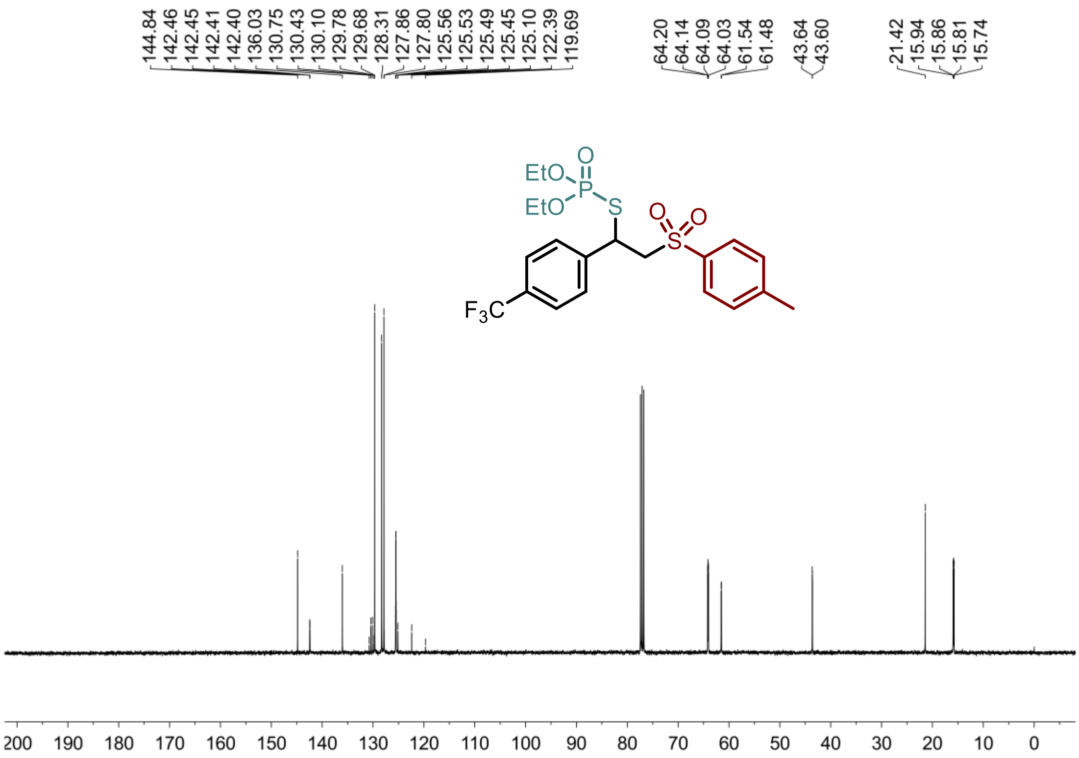

^{19}F NMR spectrum of compound **4i** (376 MHz, $CDCl_3$)

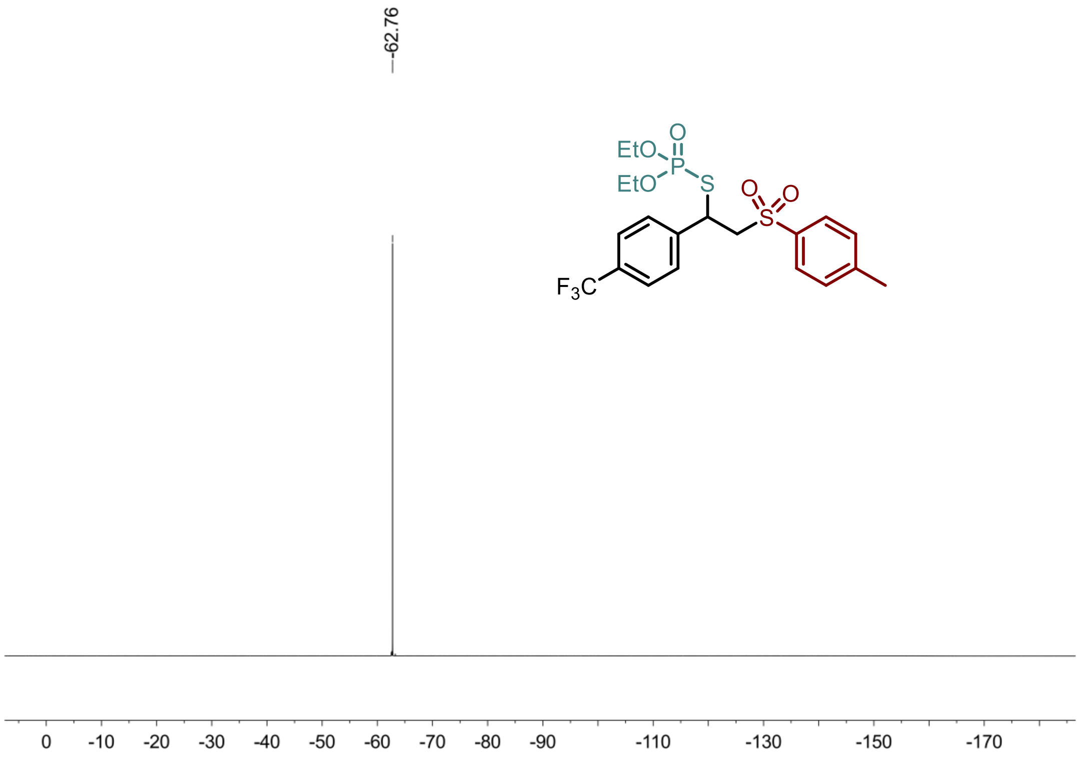

^{1}H NMR spectrum of compound **4j** (400 MHz, $CDCl_3$)

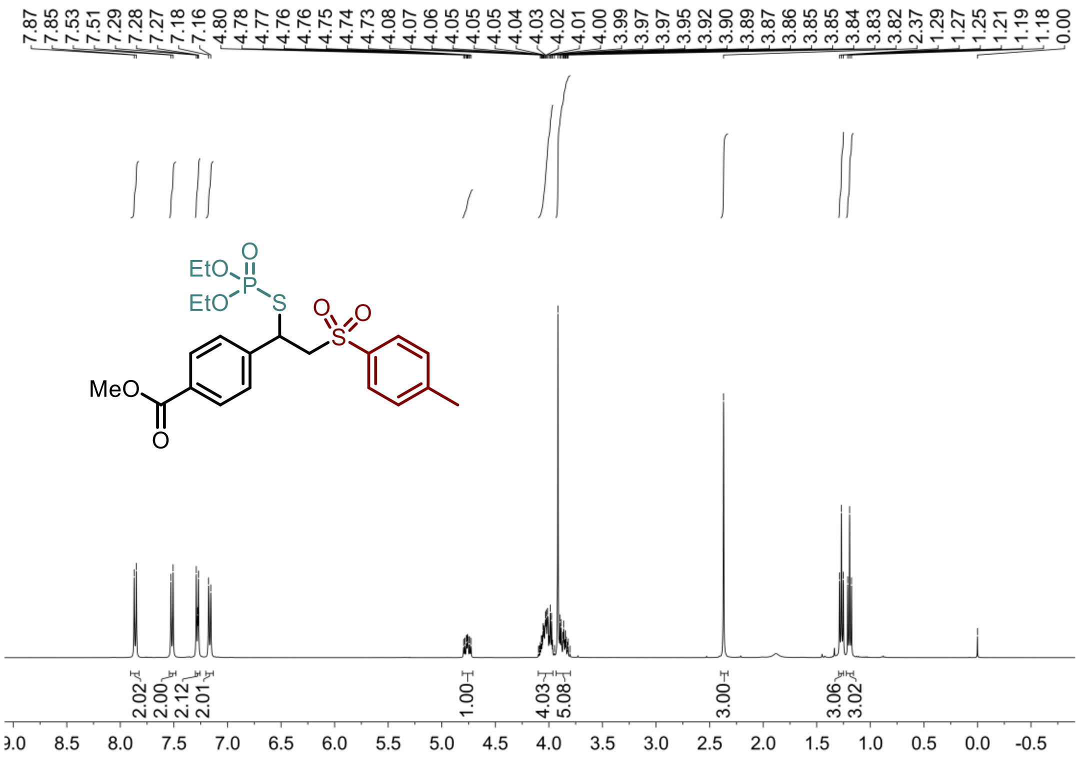

^{13}C NMR spectrum of compound **4j** (100 MHz, $CDCl_3$)

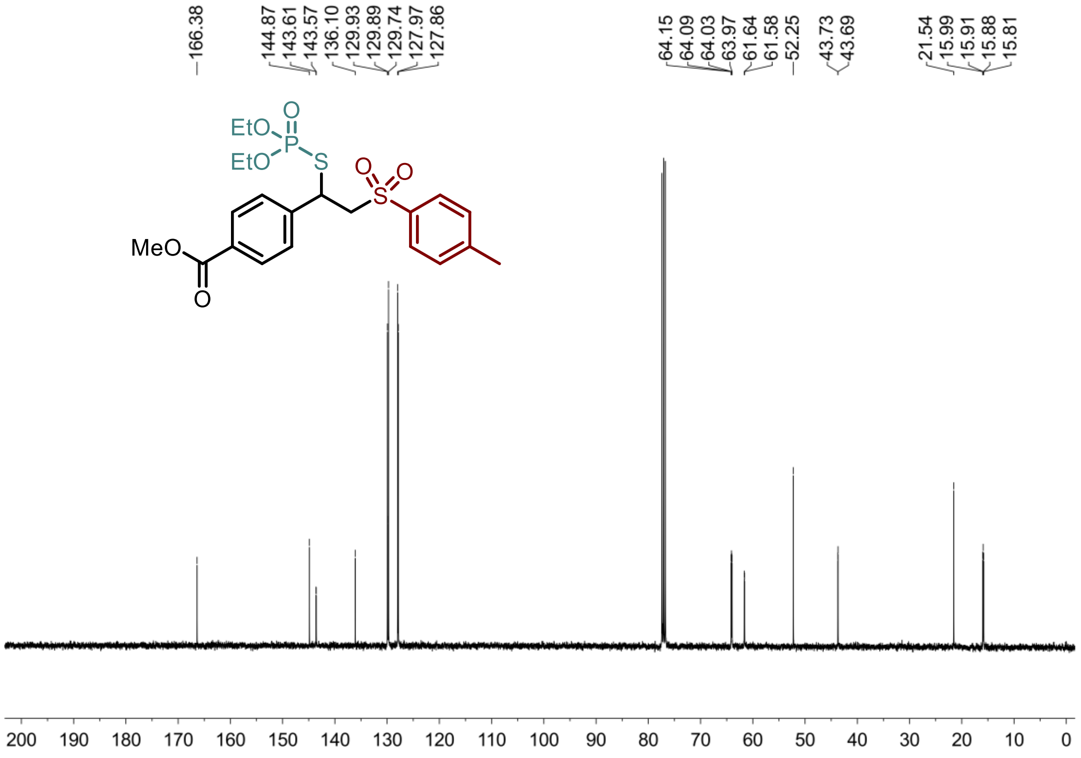

^{1}H NMR spectrum of compound **4k** (400 MHz, $CDCl_3$)

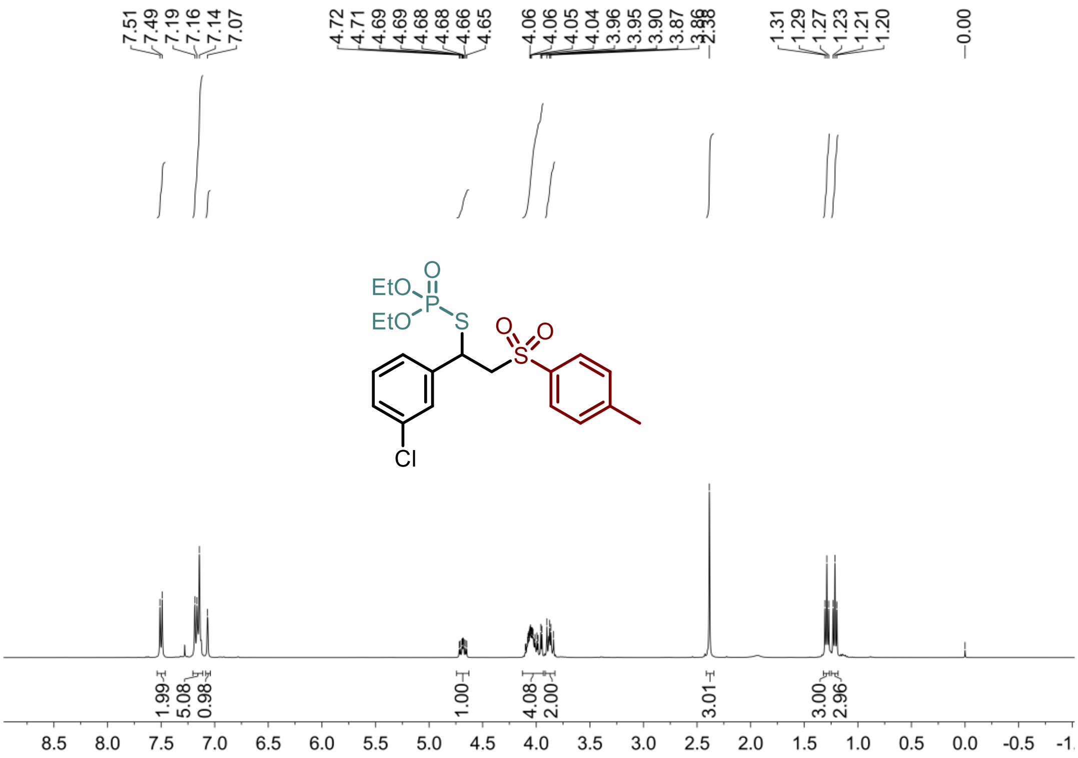

^{13}C NMR spectrum of compound **4k** (100 MHz, $CDCl_3$)

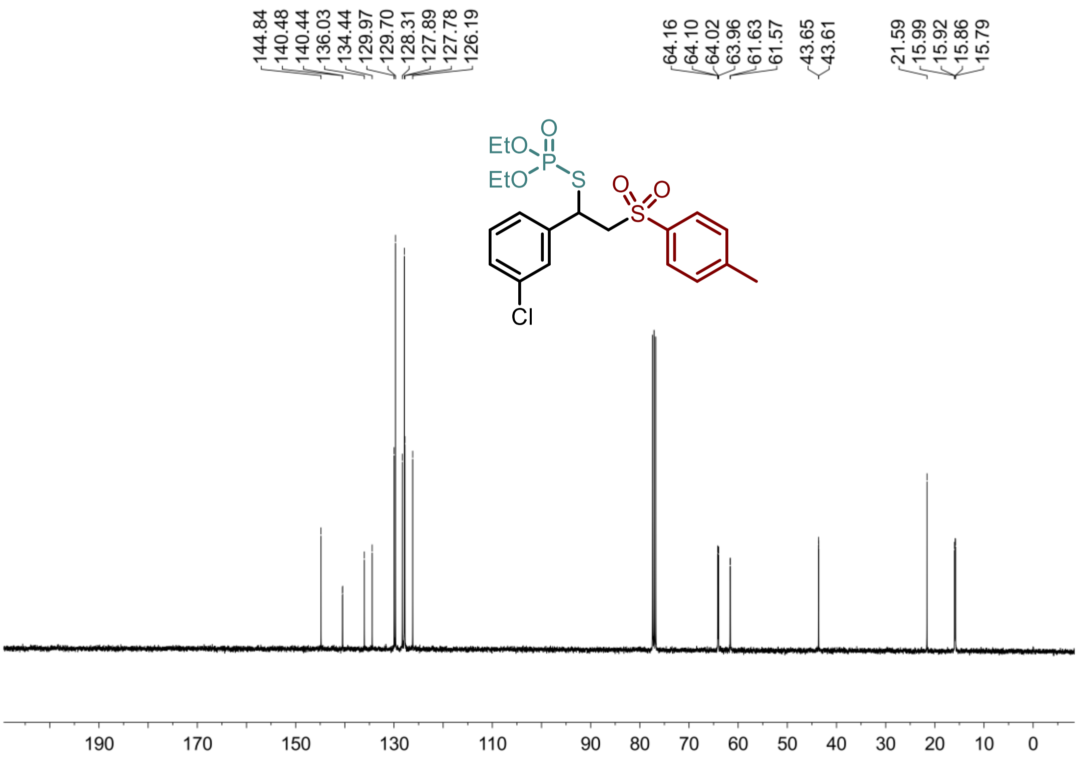

^{1}H NMR spectrum of compound **4l** (400 MHz, $CDCl_3$)

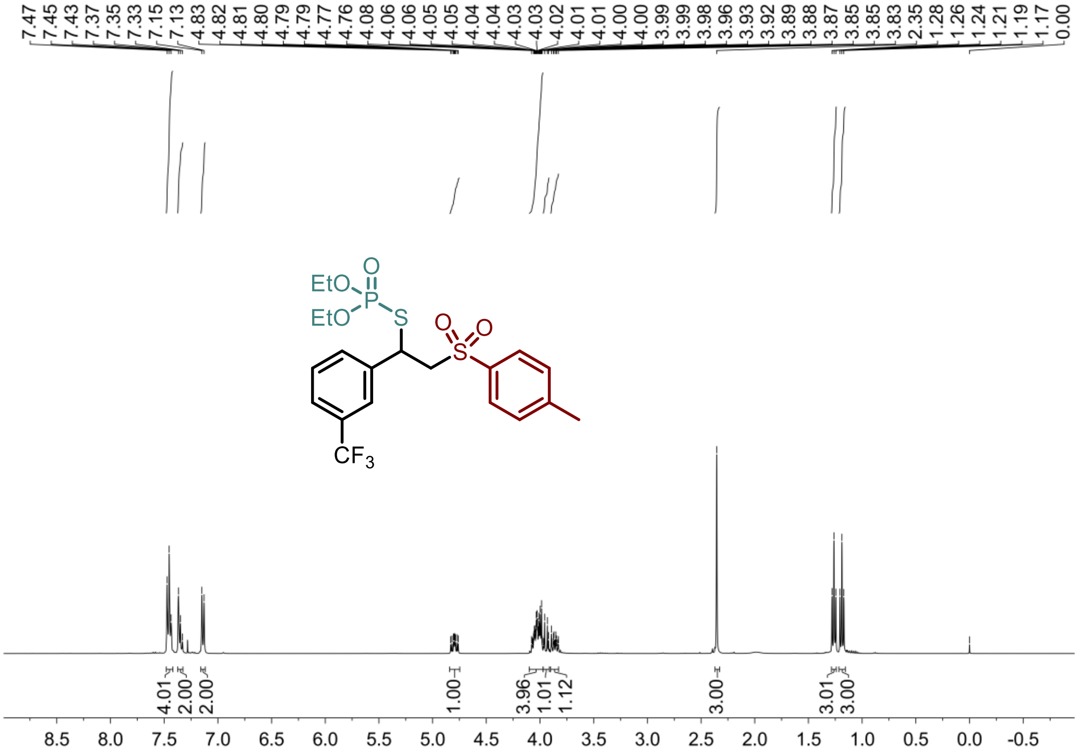

^{13}C NMR spectrum of compound **4l** (100 MHz, $CDCl_3$)

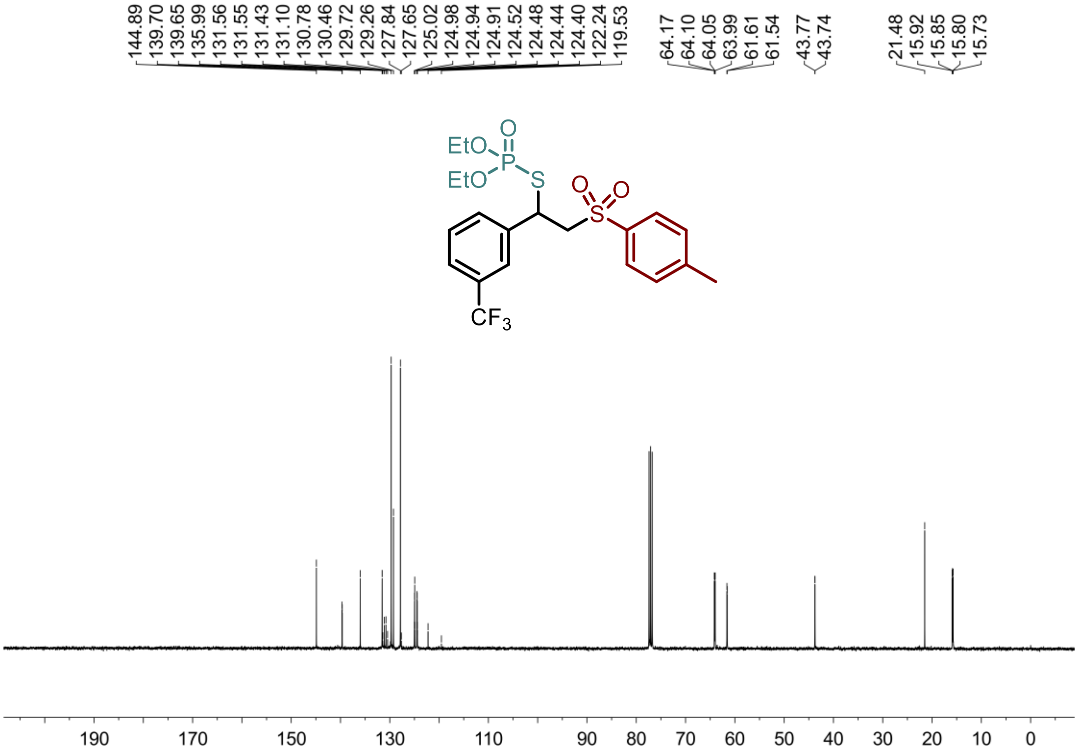

^{19}F NMR spectrum of compound **4l** (376 MHz, $CDCl_3$)

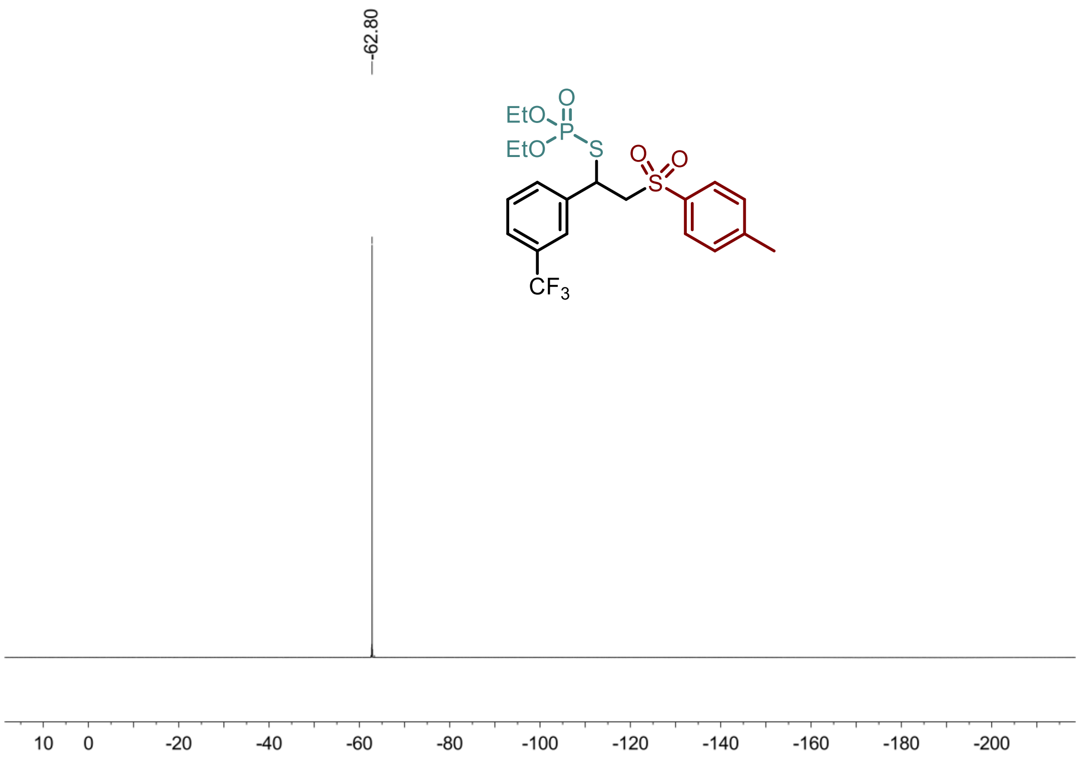

^{1}H NMR spectrum of compound 4**m** (400 MHz, $CDCl_3$)

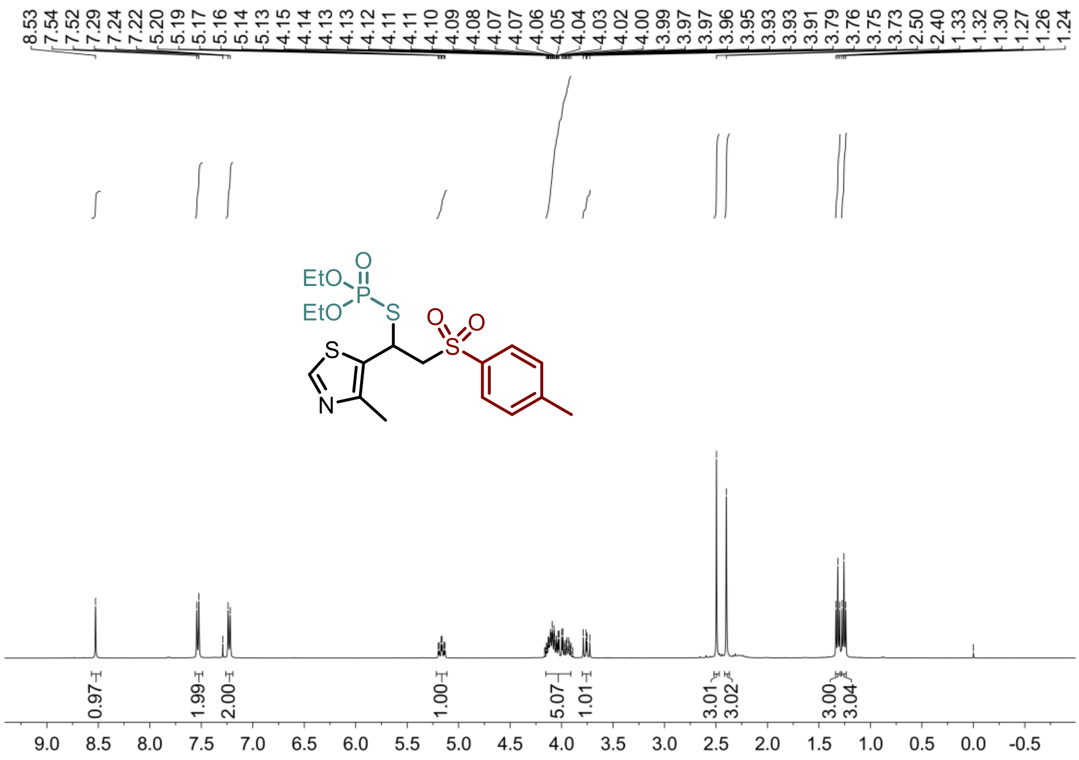

^{13}C NMR spectrum of compound **4m** (100 MHz, $CDCl_3$)

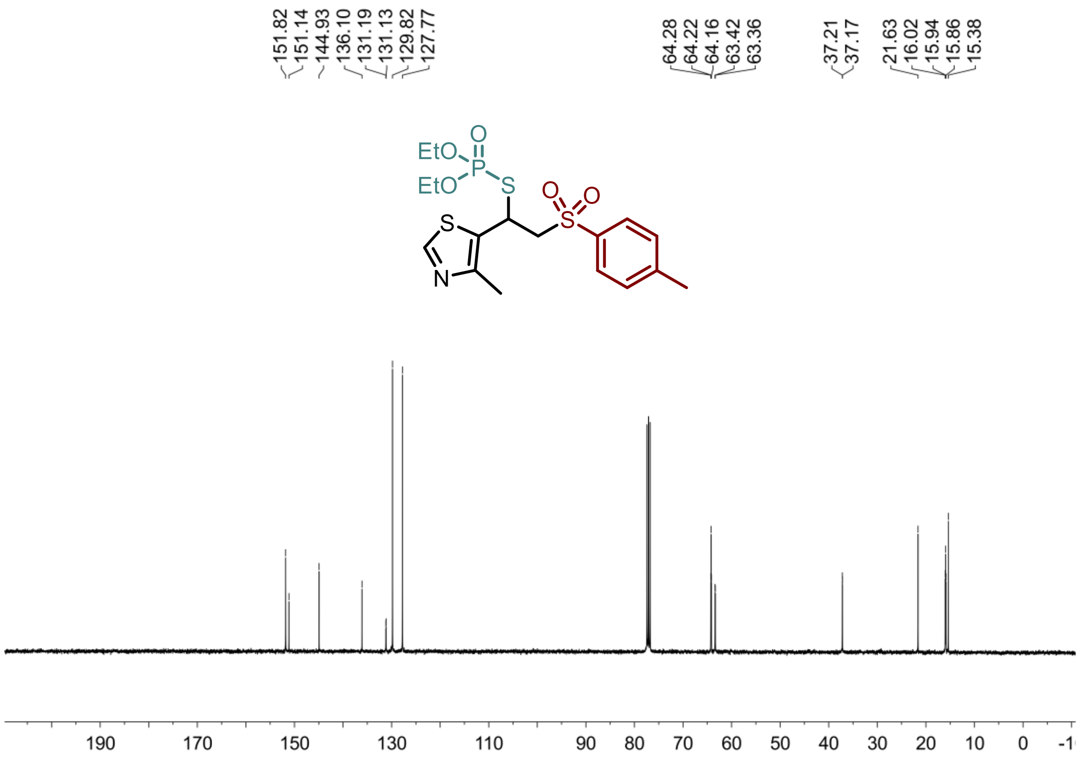

^{1}H NMR spectrum of compound **4n** (400 MHz, $CDCl_3$)

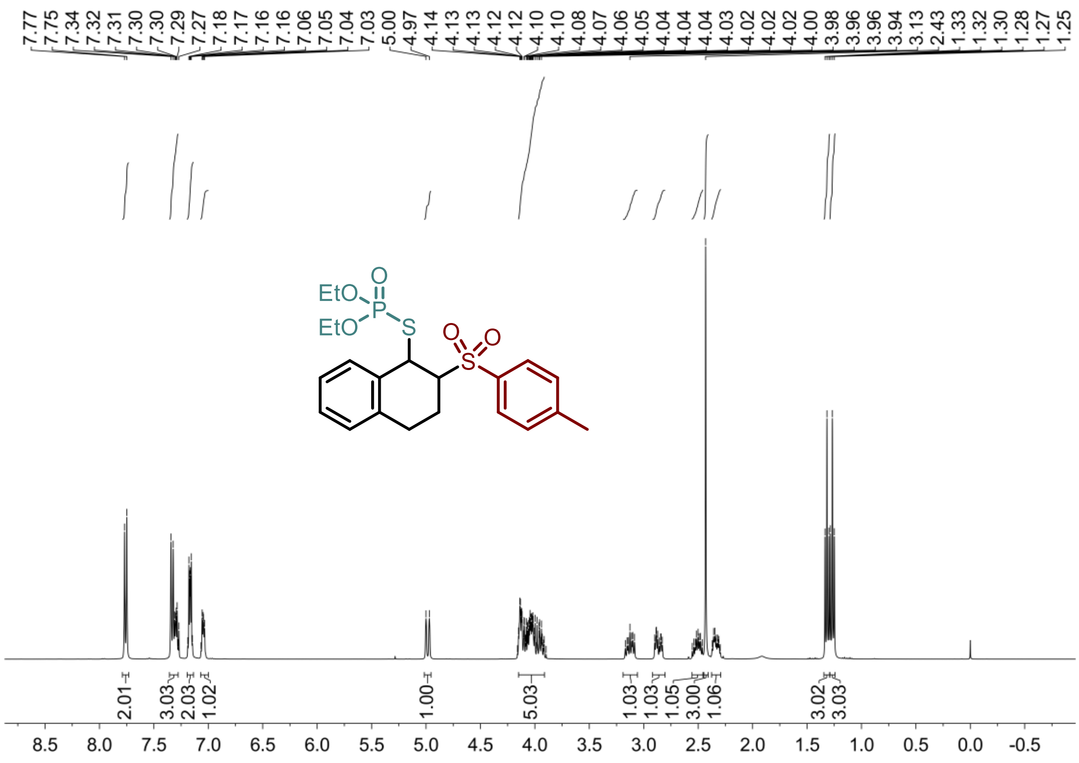

^{13}C NMR spectrum of compound 4**n** (100 MHz, $CDCl_3$)

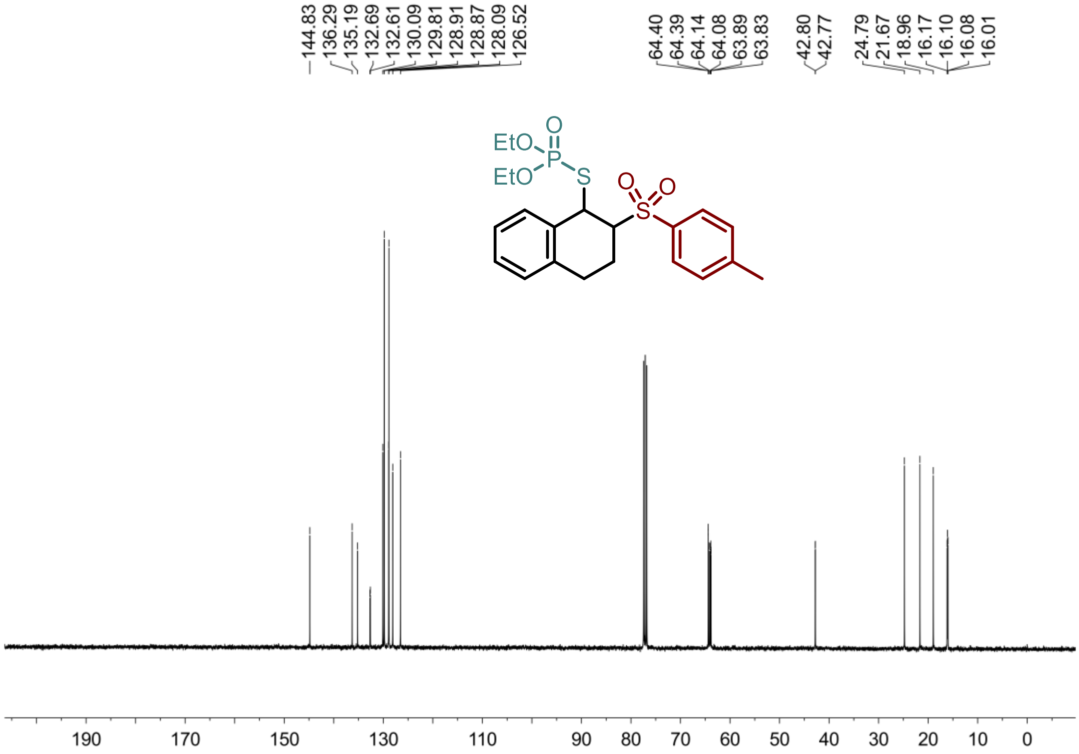

^{1}H NMR spectrum of compound **4o** (400 MHz, $CDCl_3$)

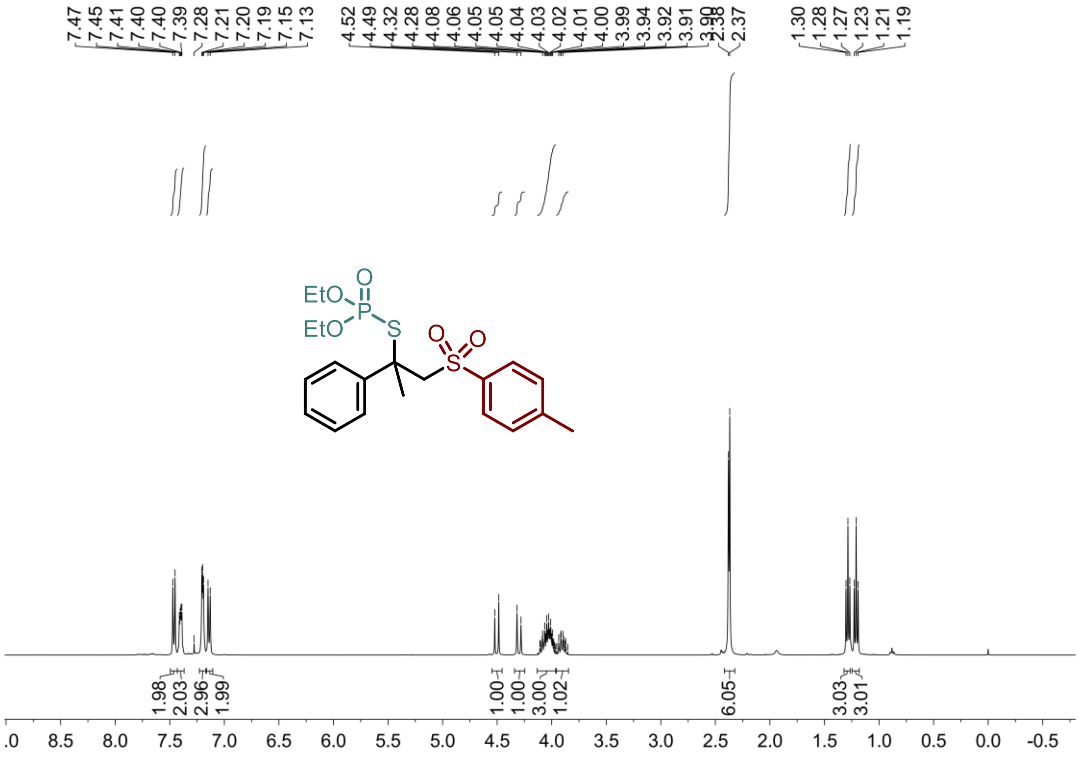

^{13}C NMR spectrum of compound **4o** (100 MHz, $CDCl_3$)

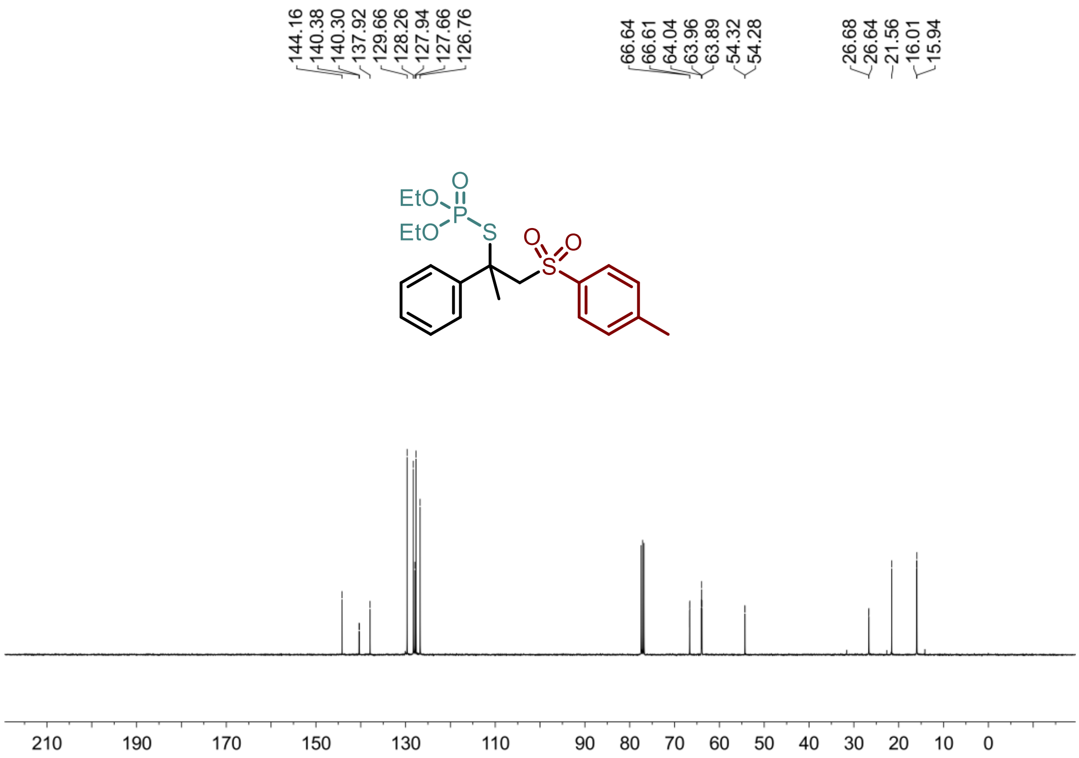

^{1}H NMR spectrum of compound **4p** (400 MHz, $CDCl_3$)

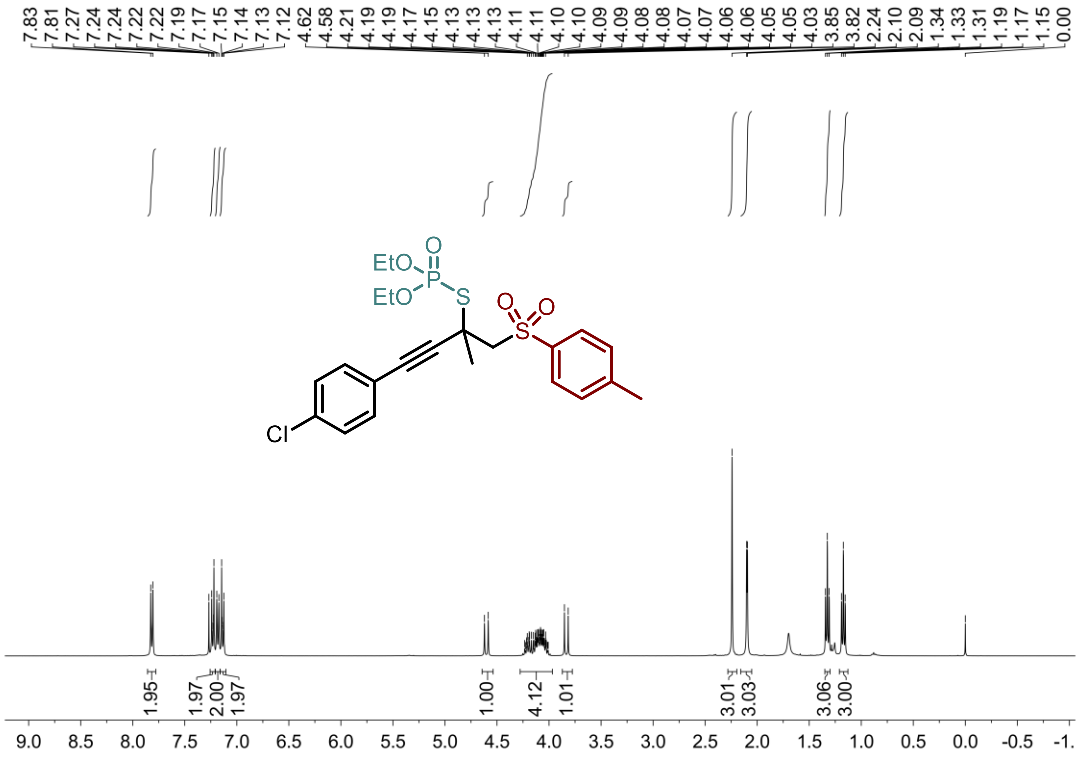

^{13}C NMR spectrum of compound **4p** (100 MHz, $CDCl_3$)

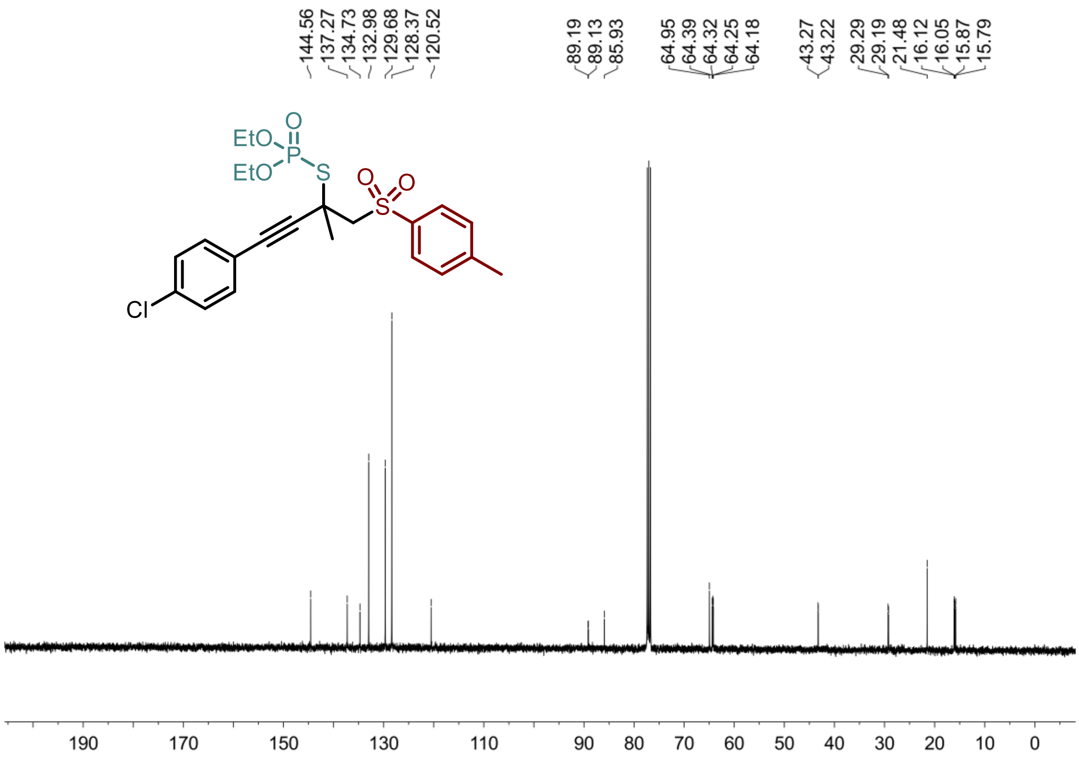

^{1}H NMR spectrum of compound 4**q** (400 MHz, $CDCl_3$)

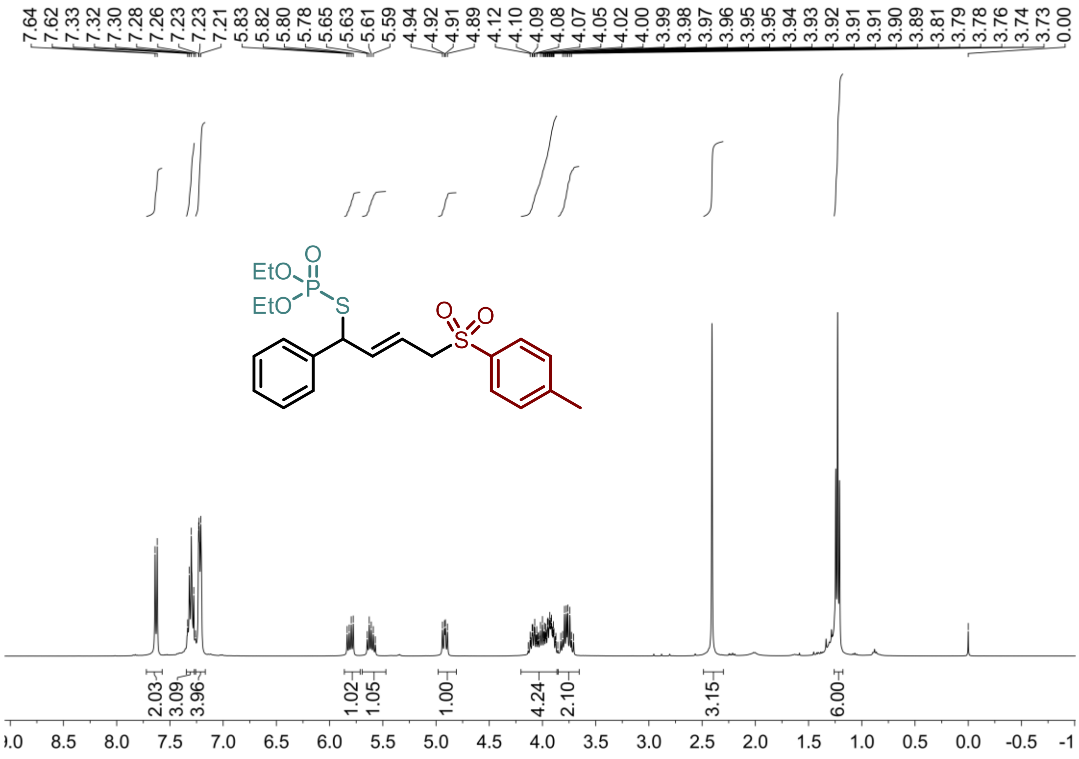

^{13}C NMR spectrum of compound **4q** (100 MHz, $CDCl_3$)

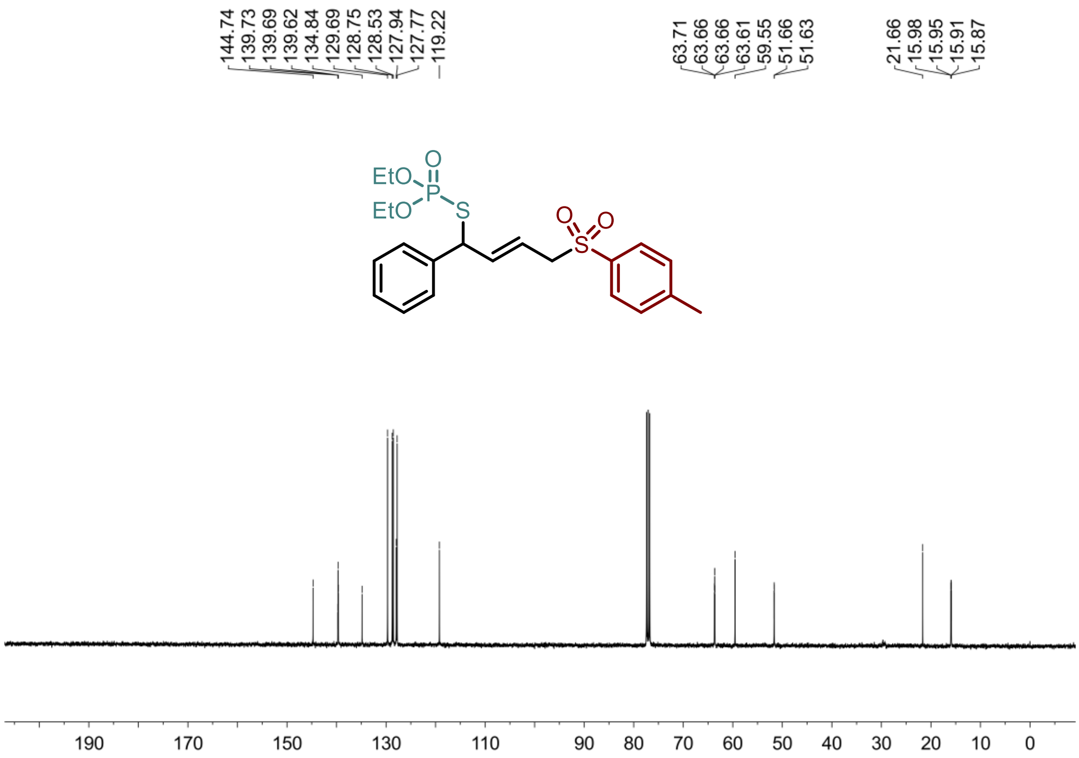

^{1}H NMR spectrum of compound **5a** (400 MHz, $CDCl_3$)

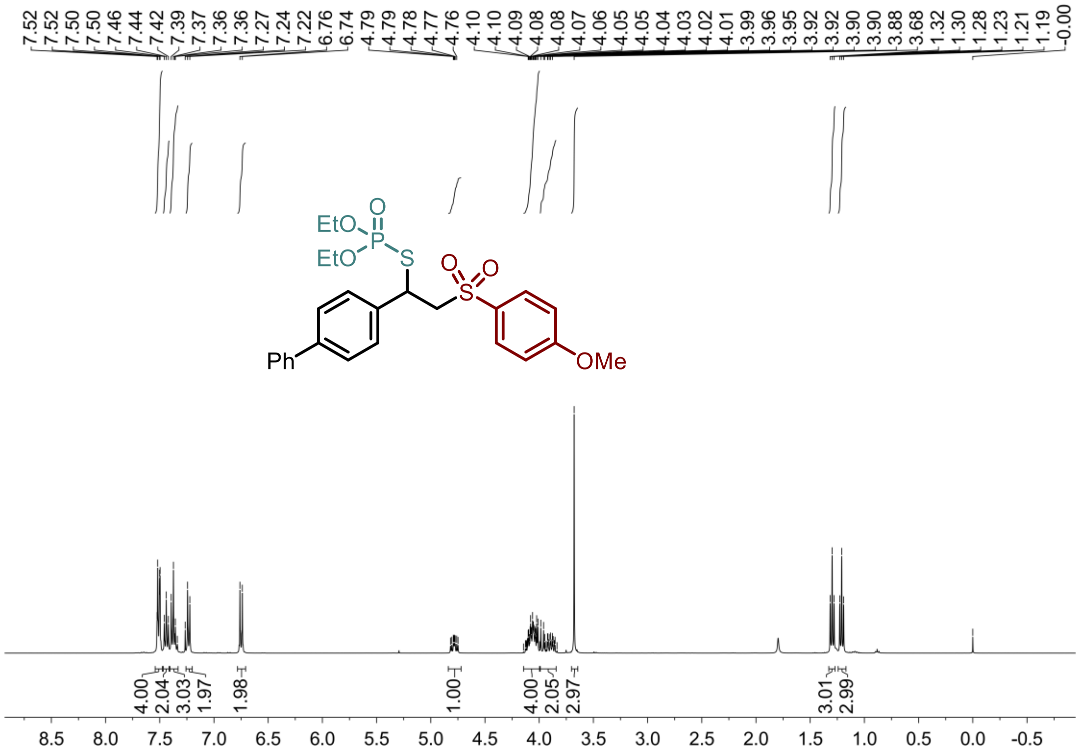

^{13}C NMR spectrum of compound **5a** (100 MHz, $CDCl_3$)

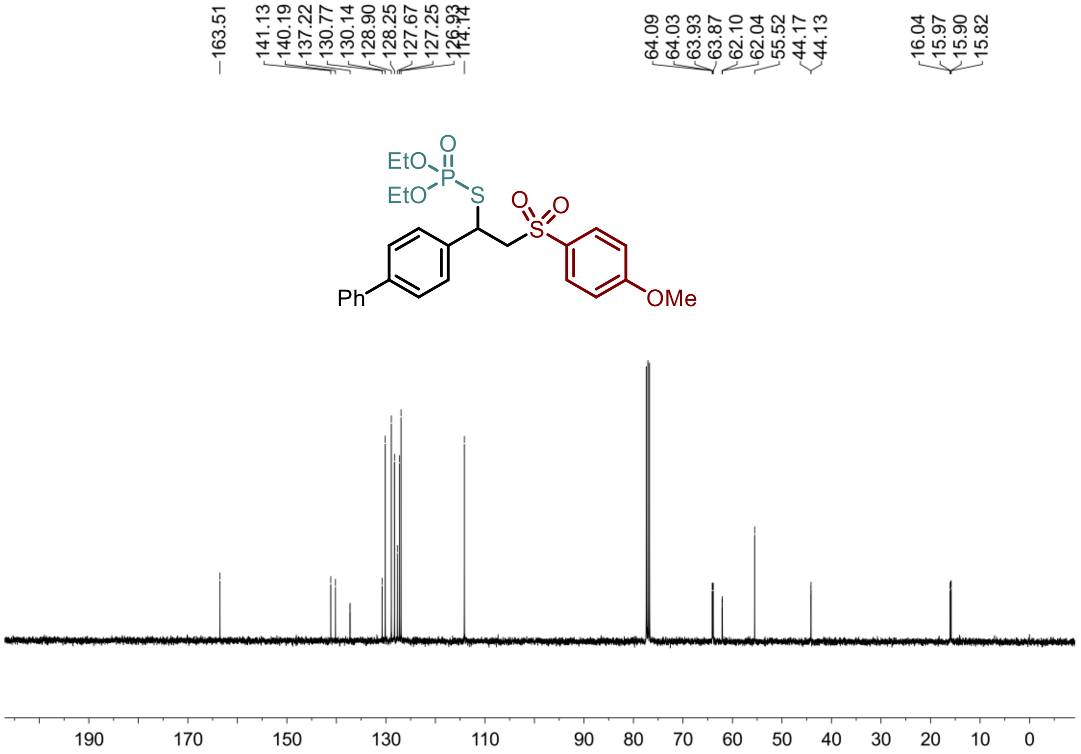

^{1}H NMR spectrum of compound **5b** (400 MHz, $CDCl_3$)

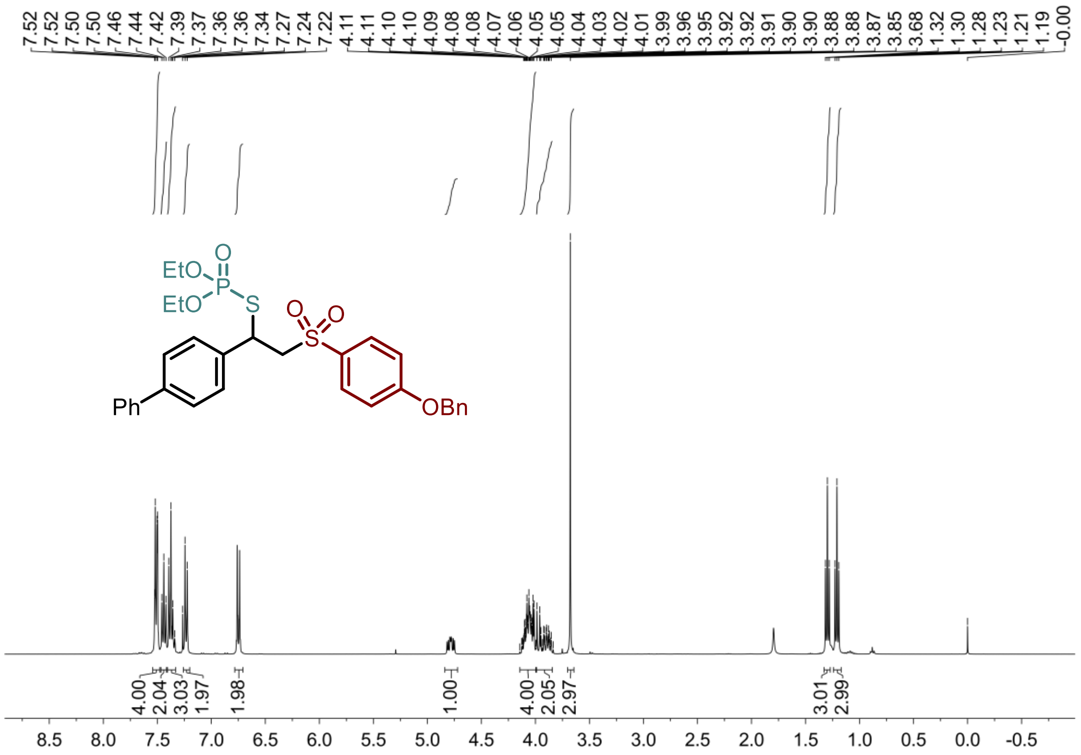

^{13}C NMR spectrum of compound **5b** (100 MHz, $CDCl_3$)

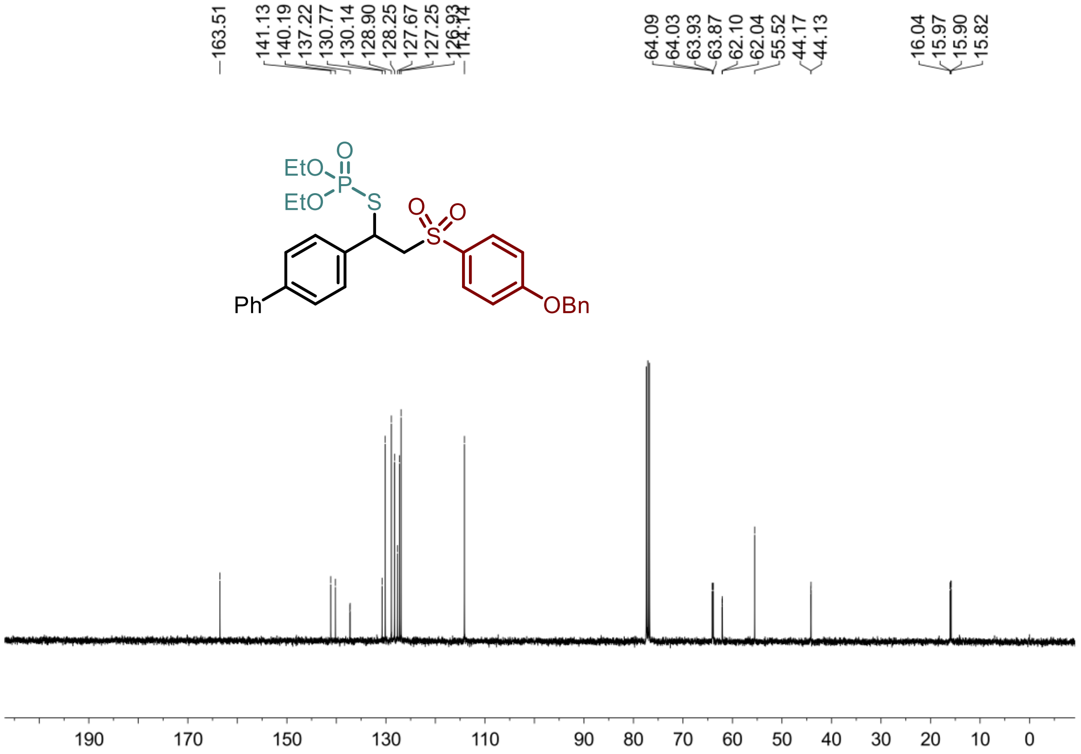

^{1}H NMR spectrum of compound **5c** (400 MHz, $CDCl_3$)

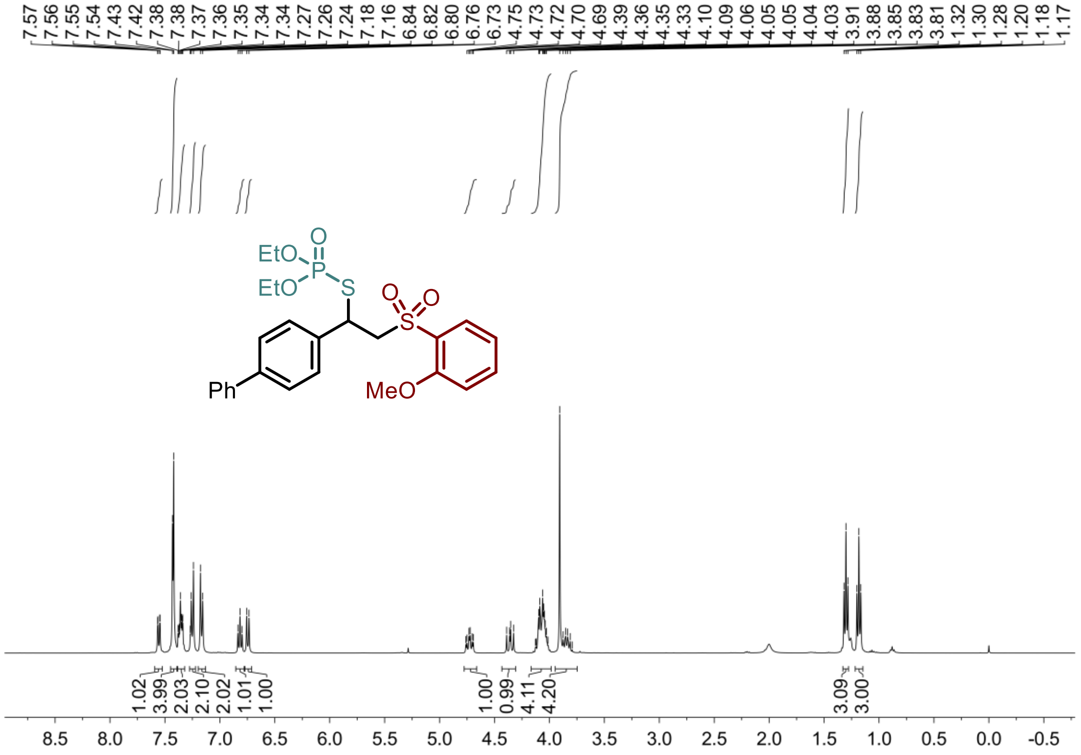

^{13}C NMR spectrum of compound **5c** (100 MHz, $CDCl_3$)

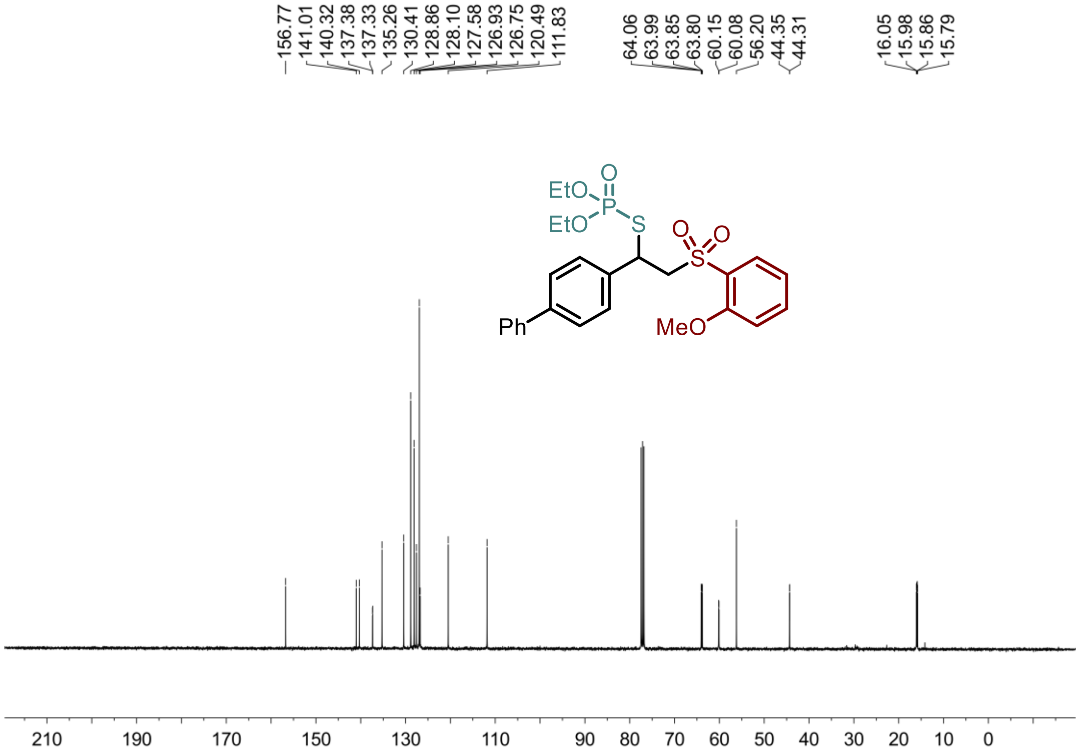

^{1}H NMR spectrum of compound **5d** (400 MHz, $CDCl_3$)

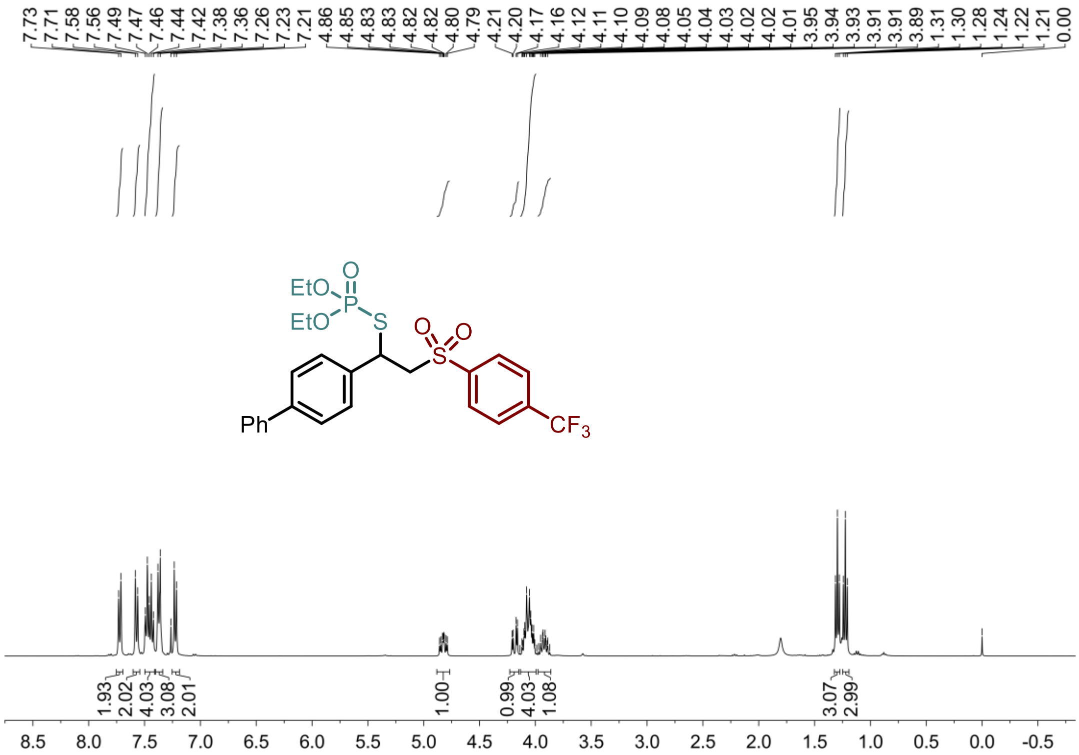

^{13}C NMR spectrum of compound **5d** (100 MHz, $CDCl_3$)

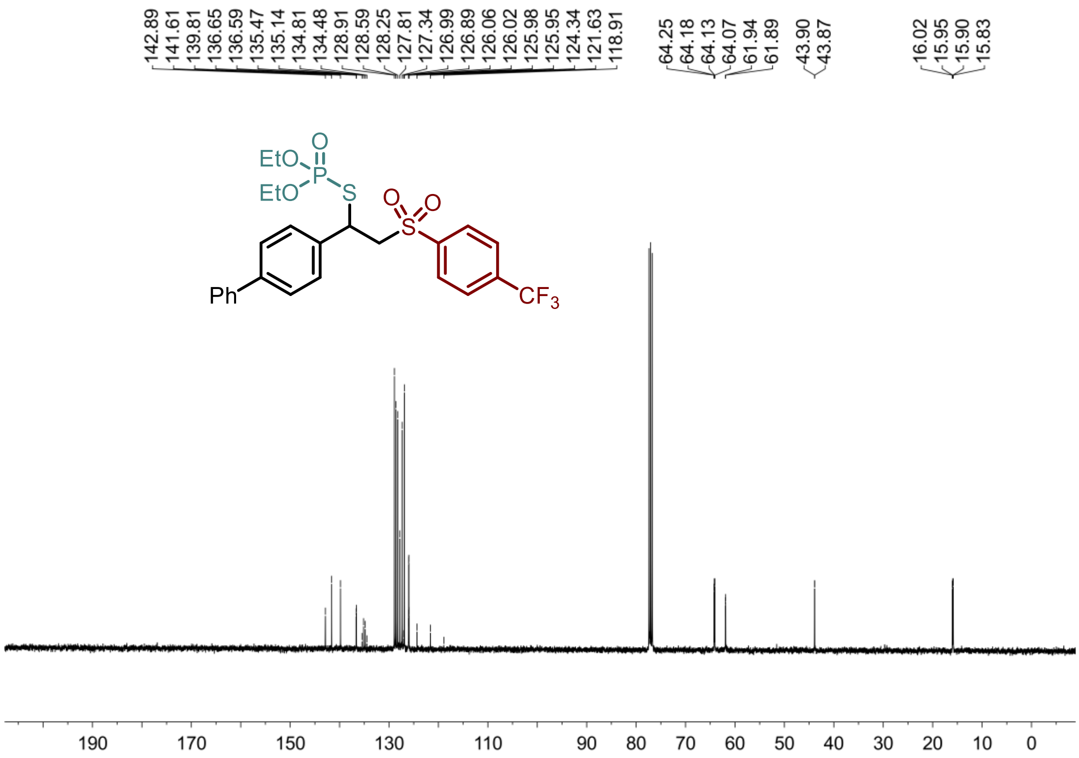

^{19}F NMR spectrum of compound **5d** (376 MHz, $CDCl_3$)

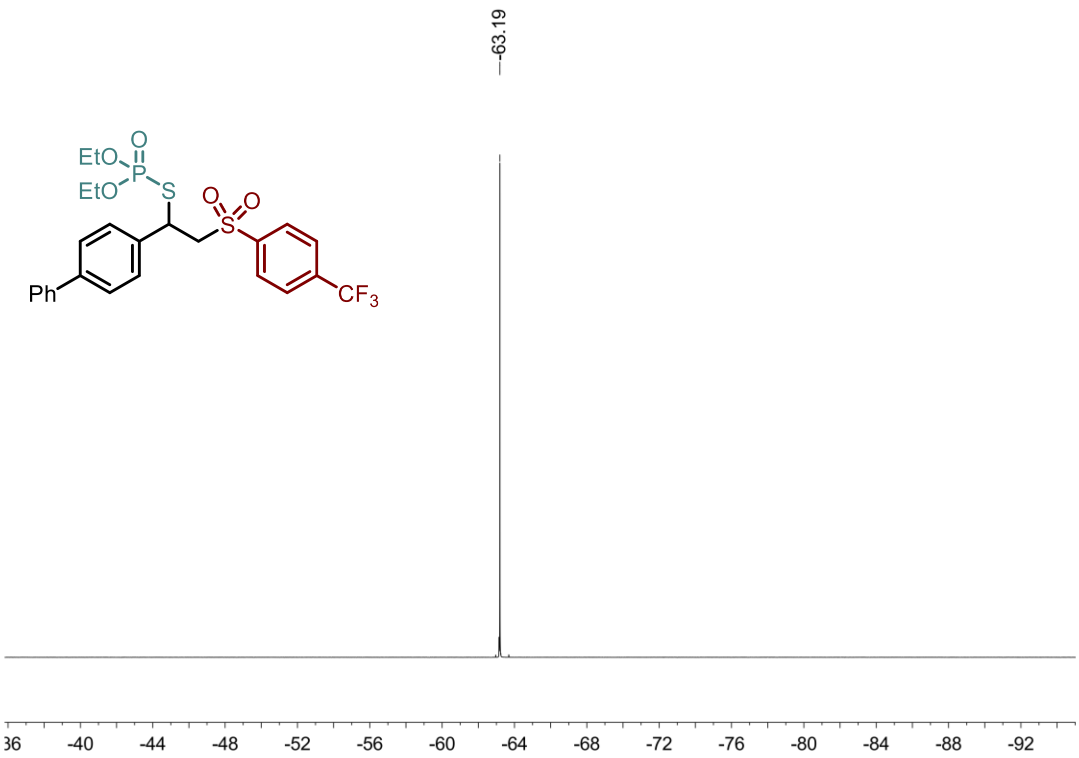

^{1}H NMR spectrum of compound **5e** (400 MHz, $CDCl_3$)

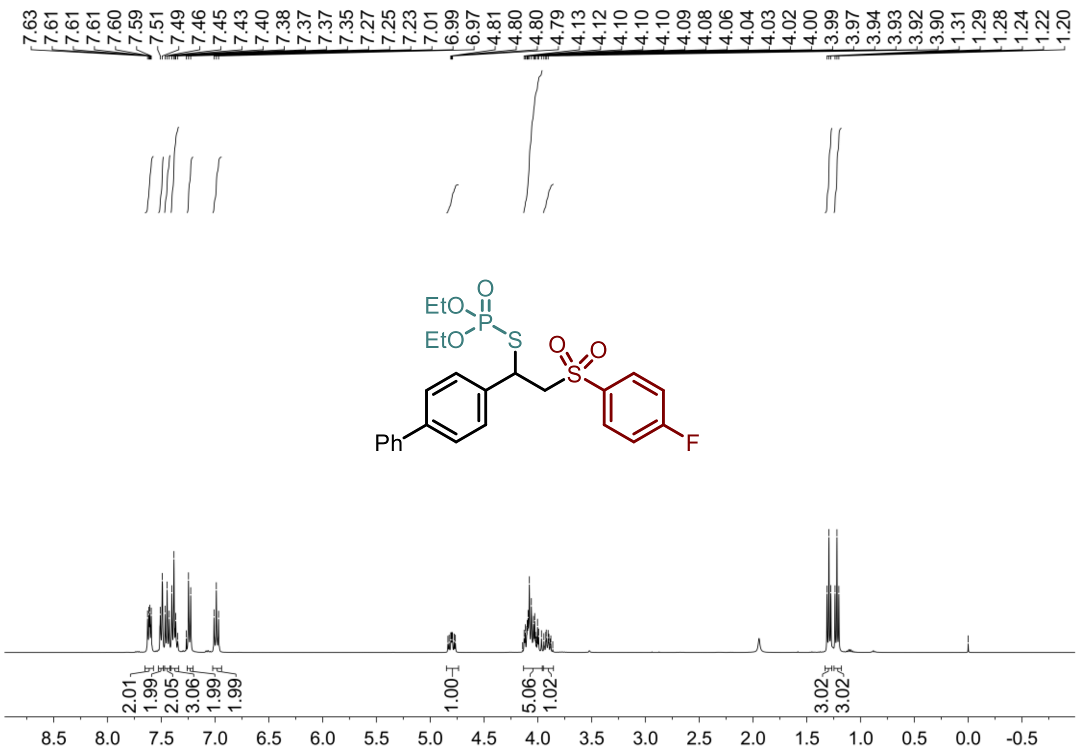

^{13}C NMR spectrum of compound **5e** (100 MHz, $CDCl_3$)

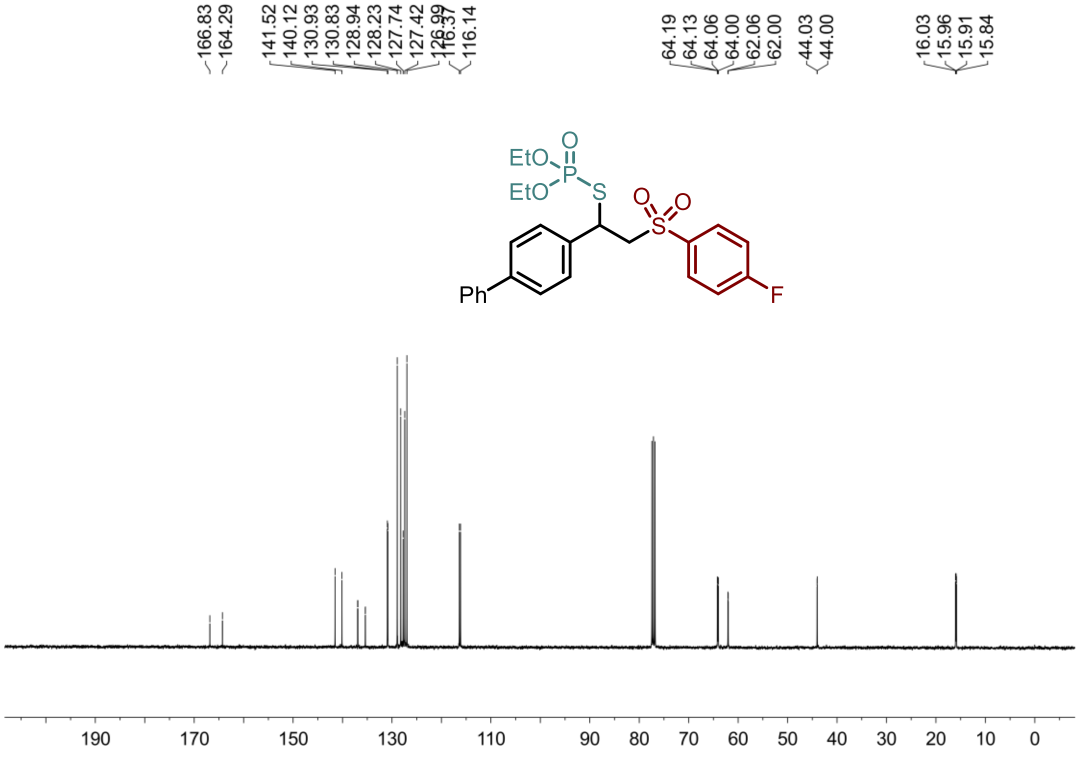

^{19}F NMR spectrum of compound **5e** (376 MHz, $CDCl_3$)

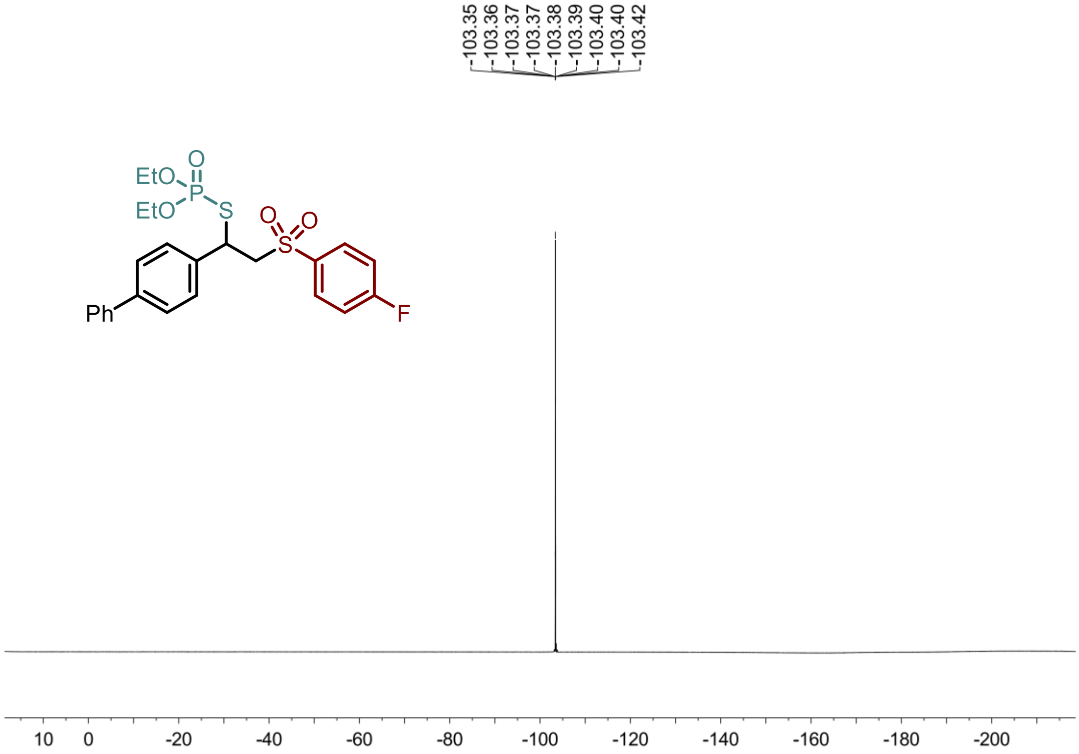

^{1}H NMR spectrum of compound **5f** (400 MHz, $CDCl_3$)

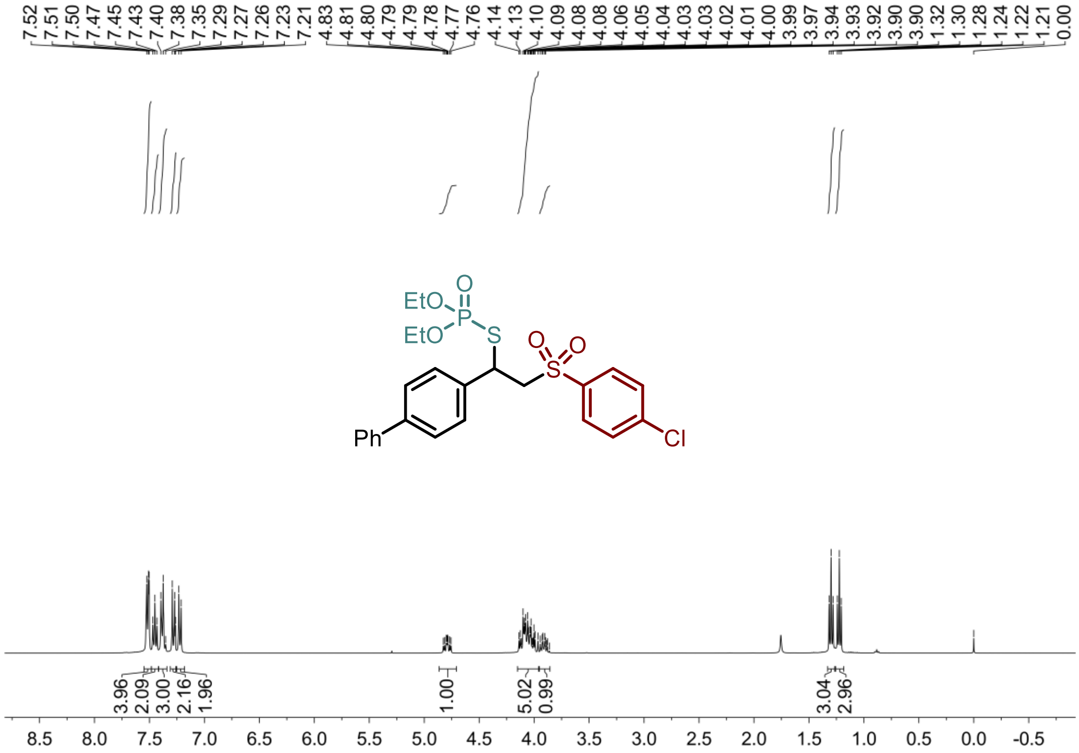

^{13}C NMR spectrum of compound **5f** (100 MHz, $CDCl_3$)

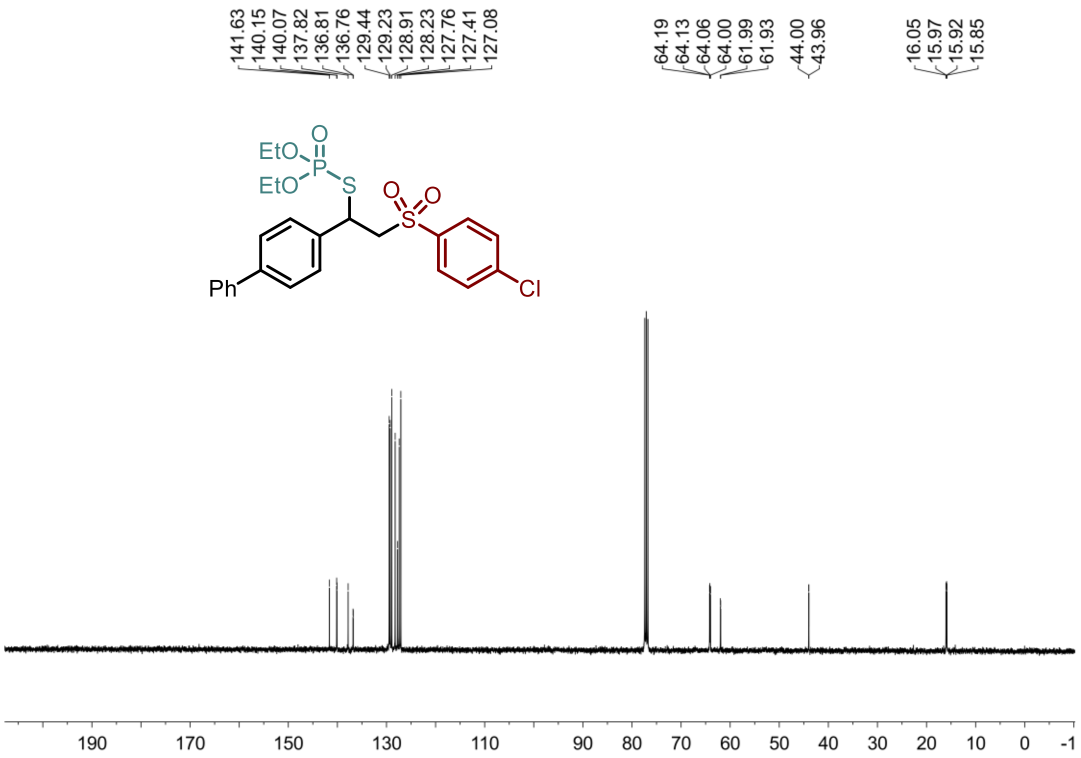

^{1}H NMR spectrum of compound **5g** (400 MHz, $CDCl_3$)

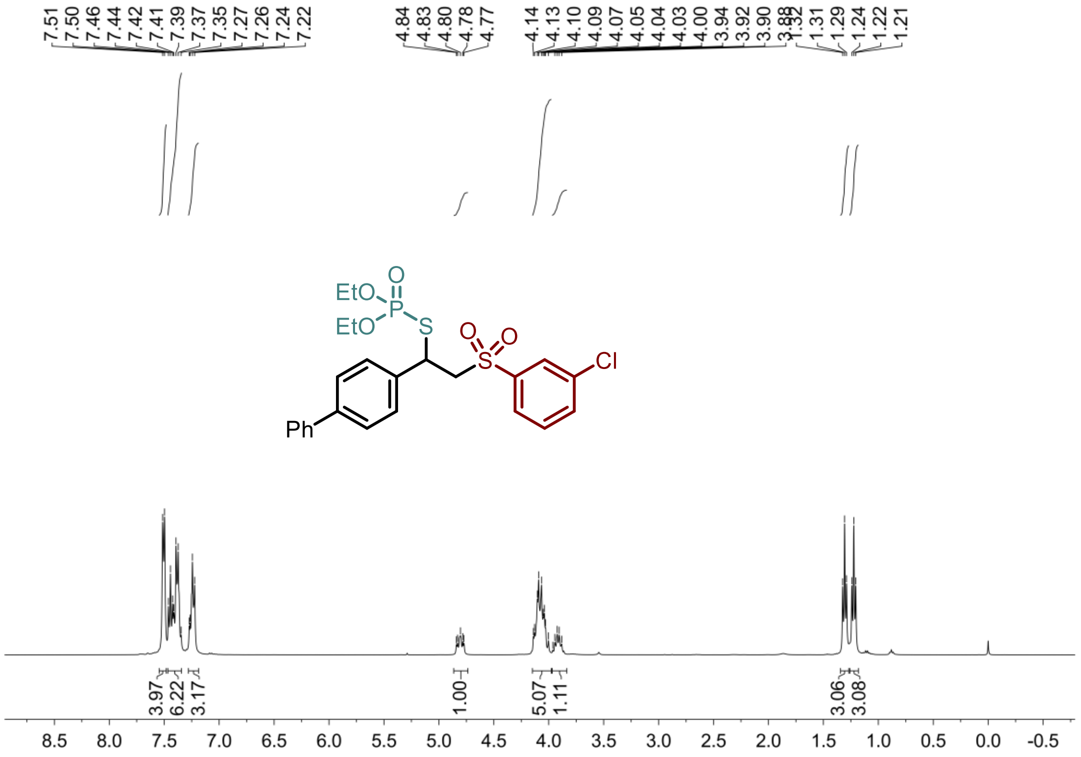

^{13}C NMR spectrum of compound **5g** (100 MHz, $CDCl_3$)

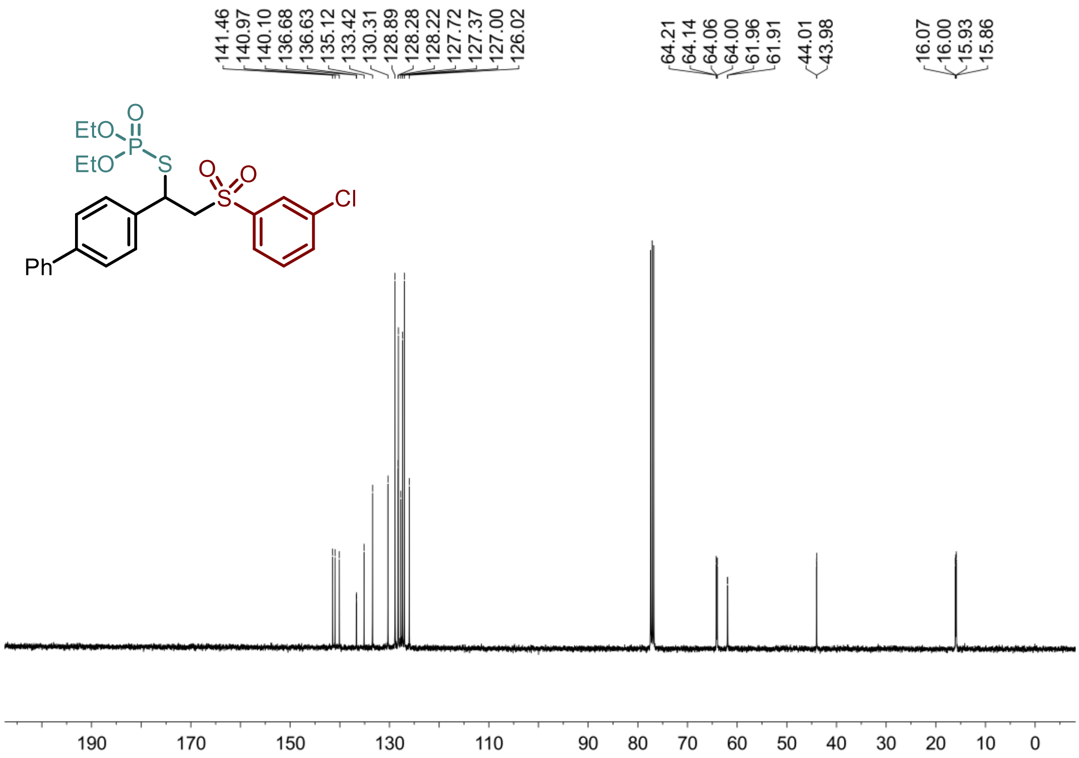

^{1}H NMR spectrum of compound **5h** (400 MHz, $CDCl_3$)

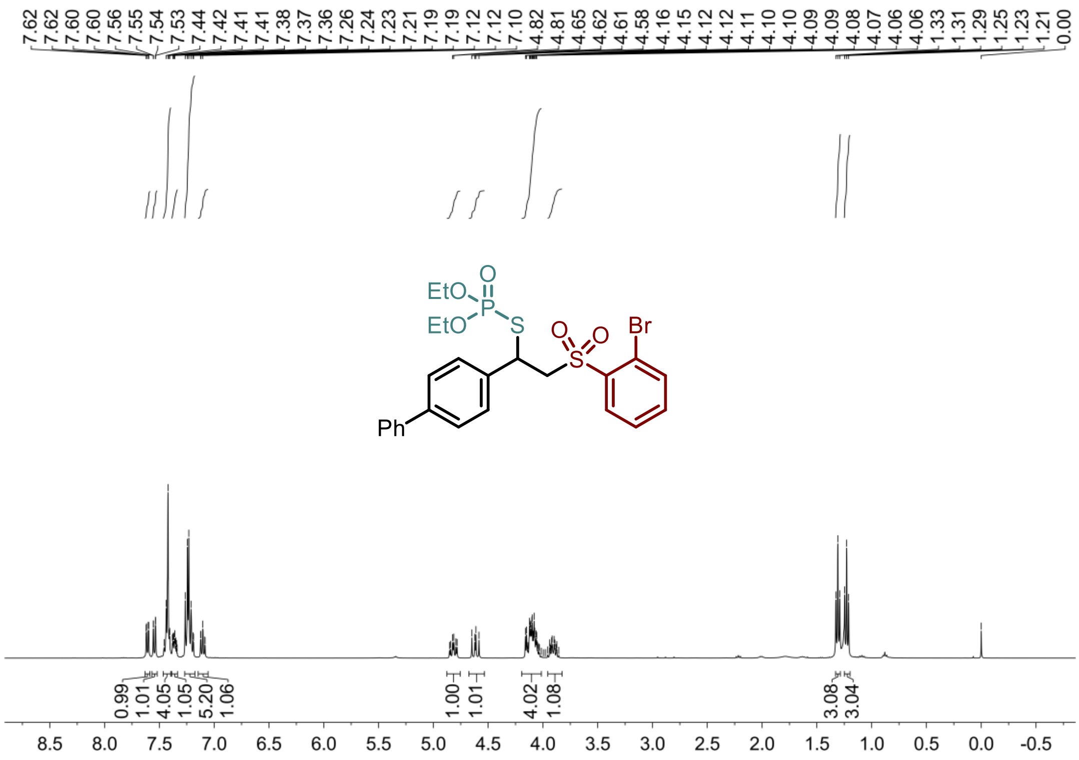

^{13}C NMR spectrum of compound **5h** (100 MHz, $CDCl_3$)

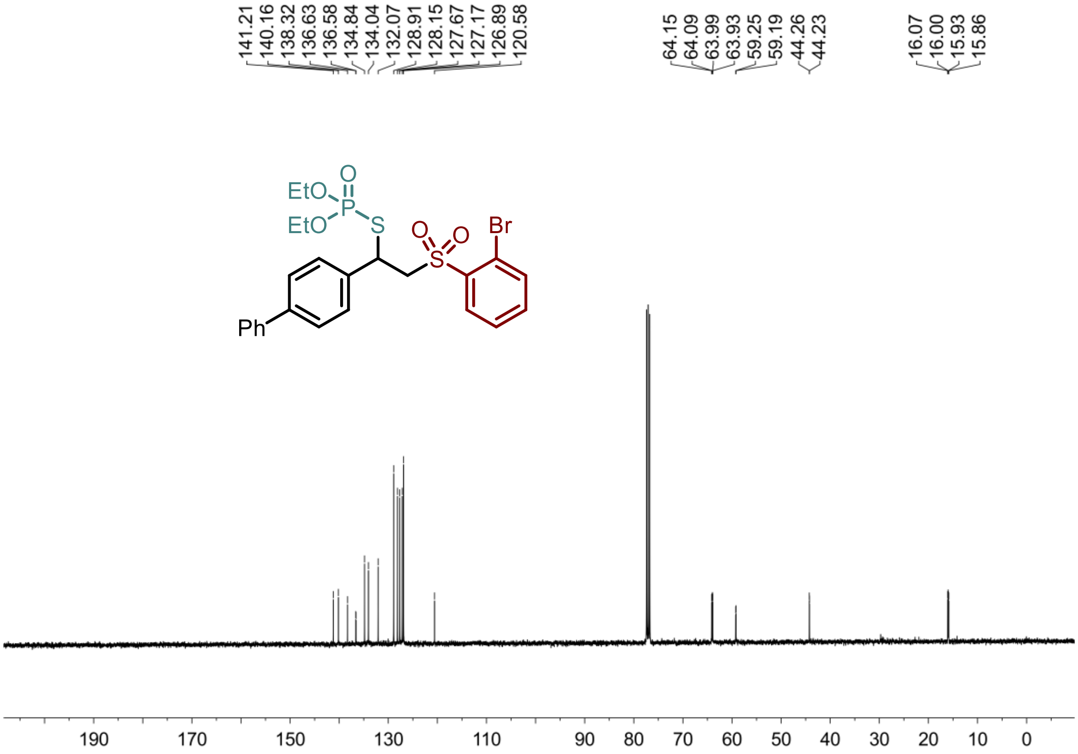

^{1}H NMR spectrum of compound **5i** (400 MHz, $CDCl_3$)

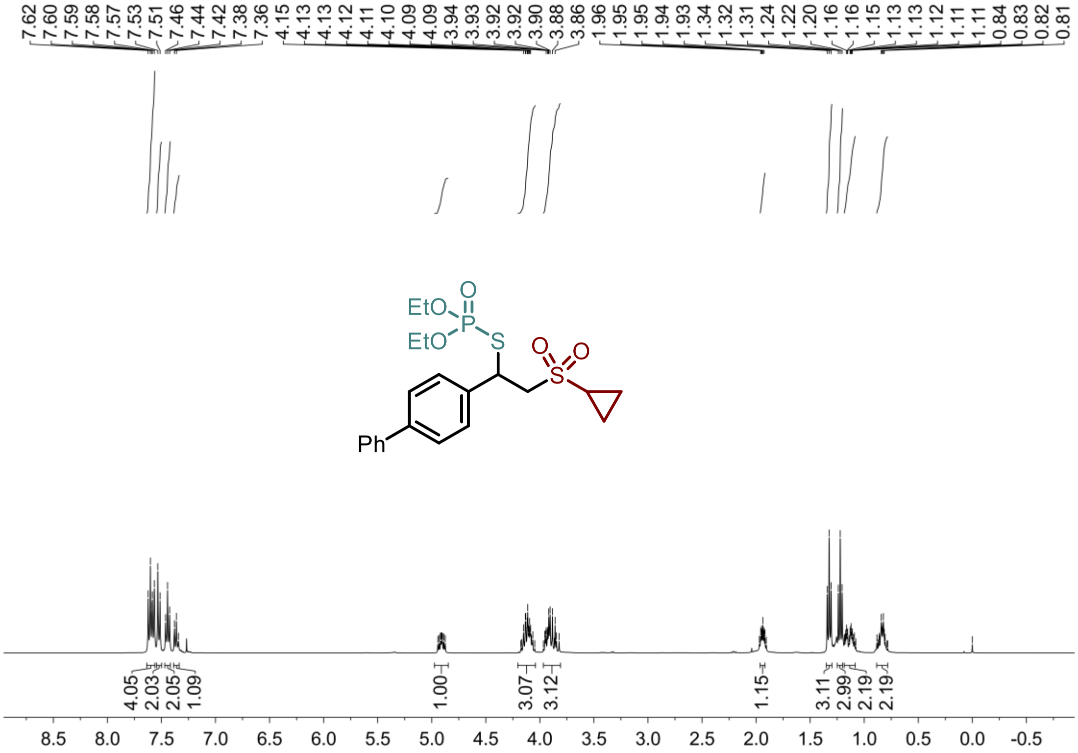

^{13}C NMR spectrum of compound **5i** (100 MHz, $CDCl_3$)

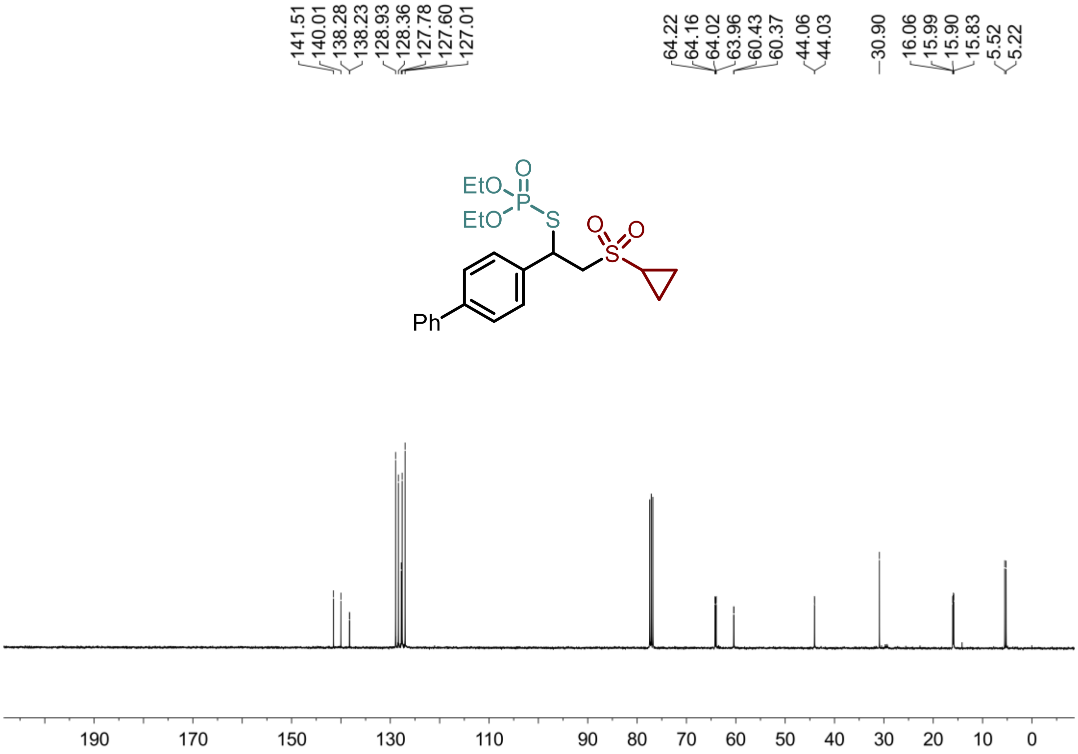

^{1}H NMR spectrum of compound **5j** (400 MHz, $CDCl_3$)

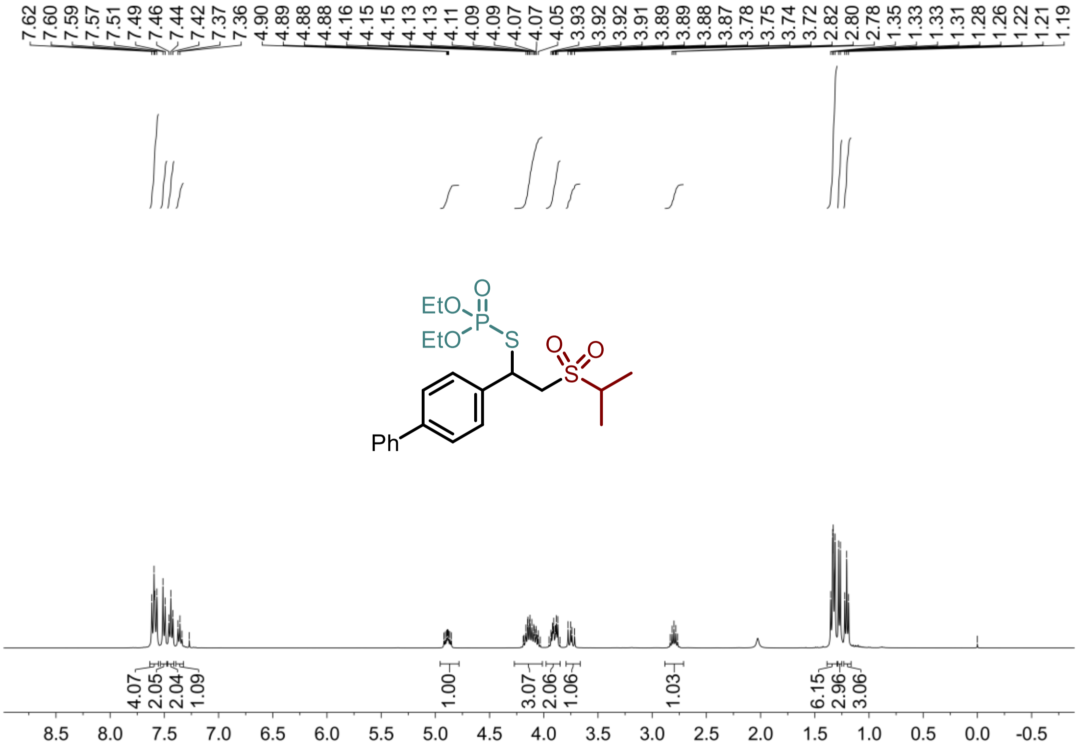

^{13}C NMR spectrum of compound **5j** (100 MHz, $CDCl_3$)

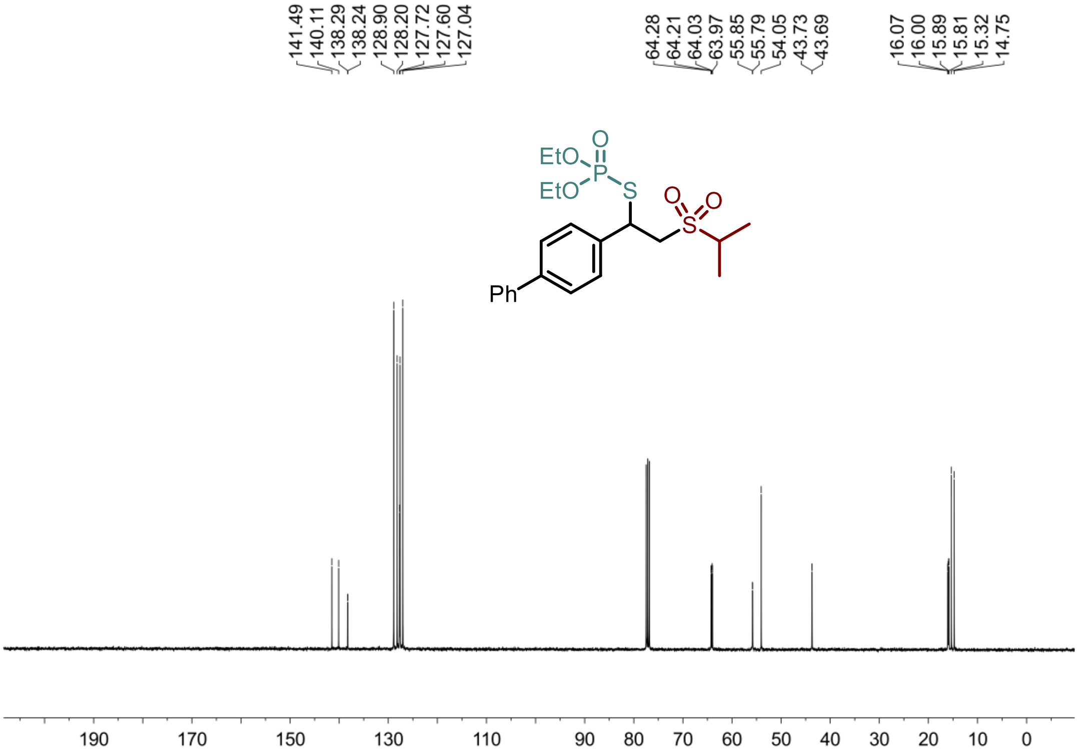

^{1}H NMR spectrum of compound **5k** (400 MHz, $CDCl_3$)\

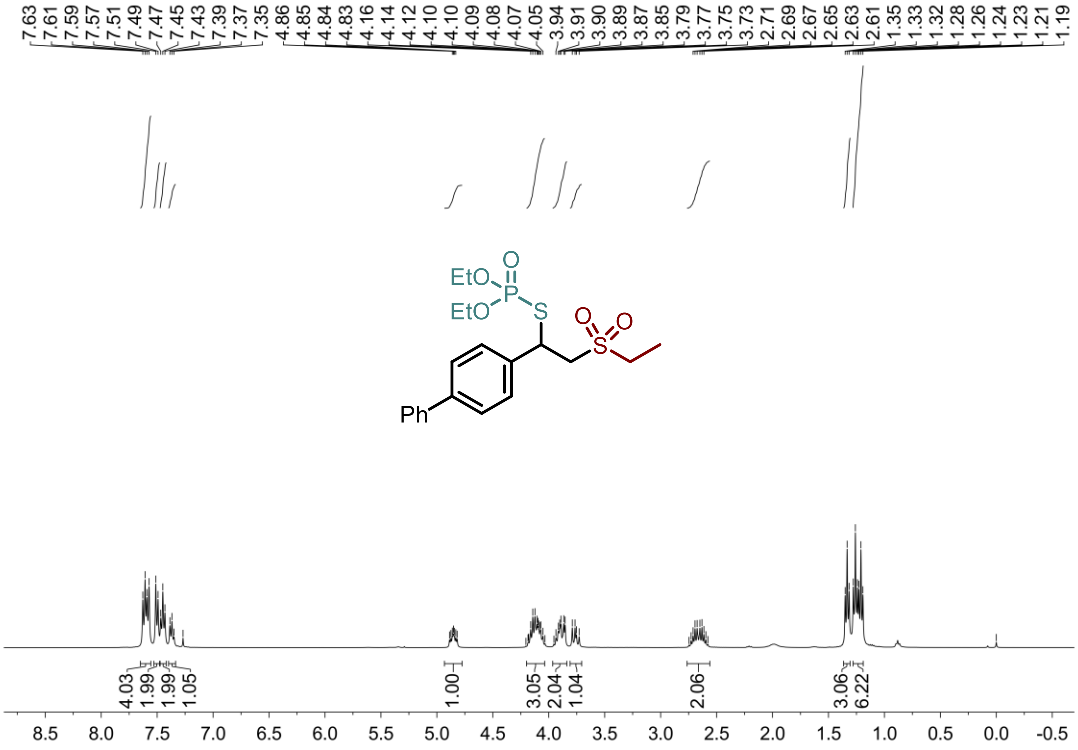

^{13}C NMR spectrum of compound **5k** (100 MHz, $CDCl_3$)

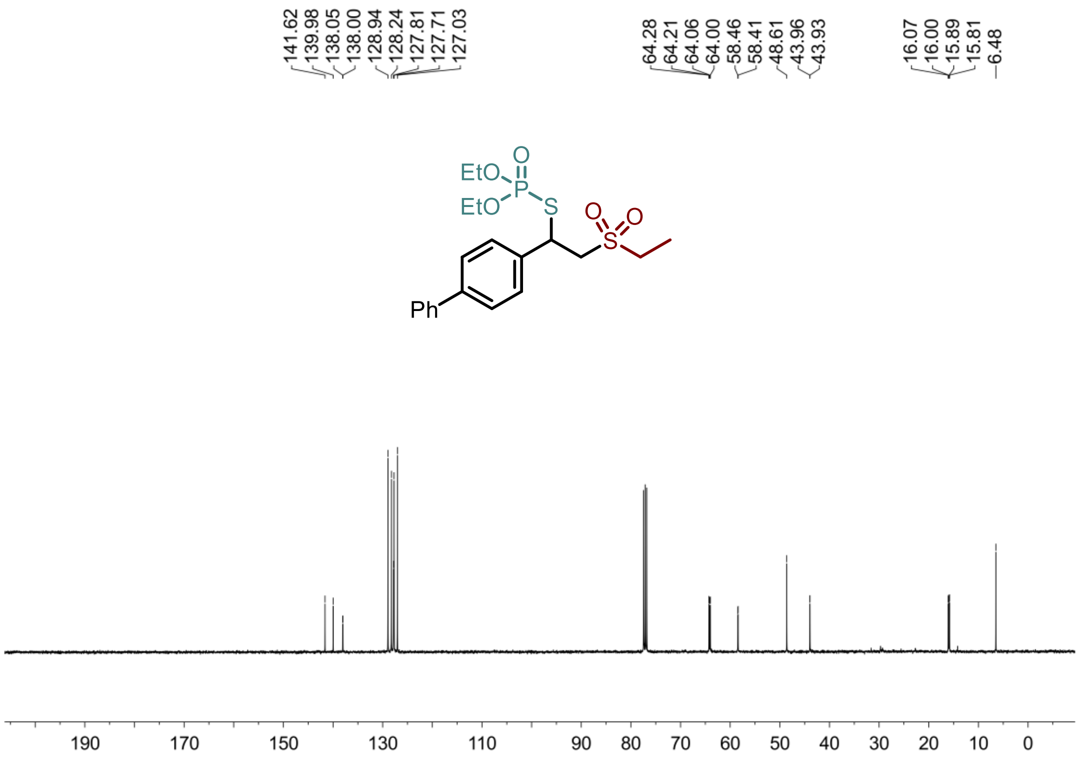

^{1}H NMR spectrum of compound **5l** (400 MHz, $CDCl_3$)

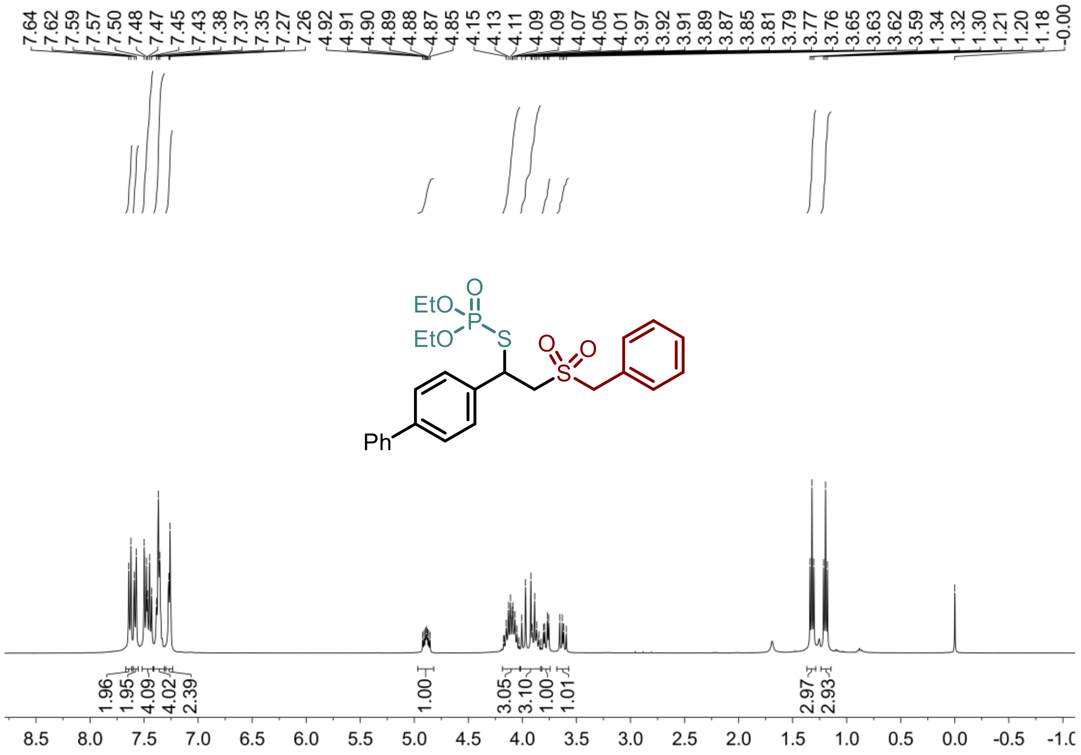

^{13}C NMR spectrum of compound **5l** (100 MHz, $CDCl_3$)

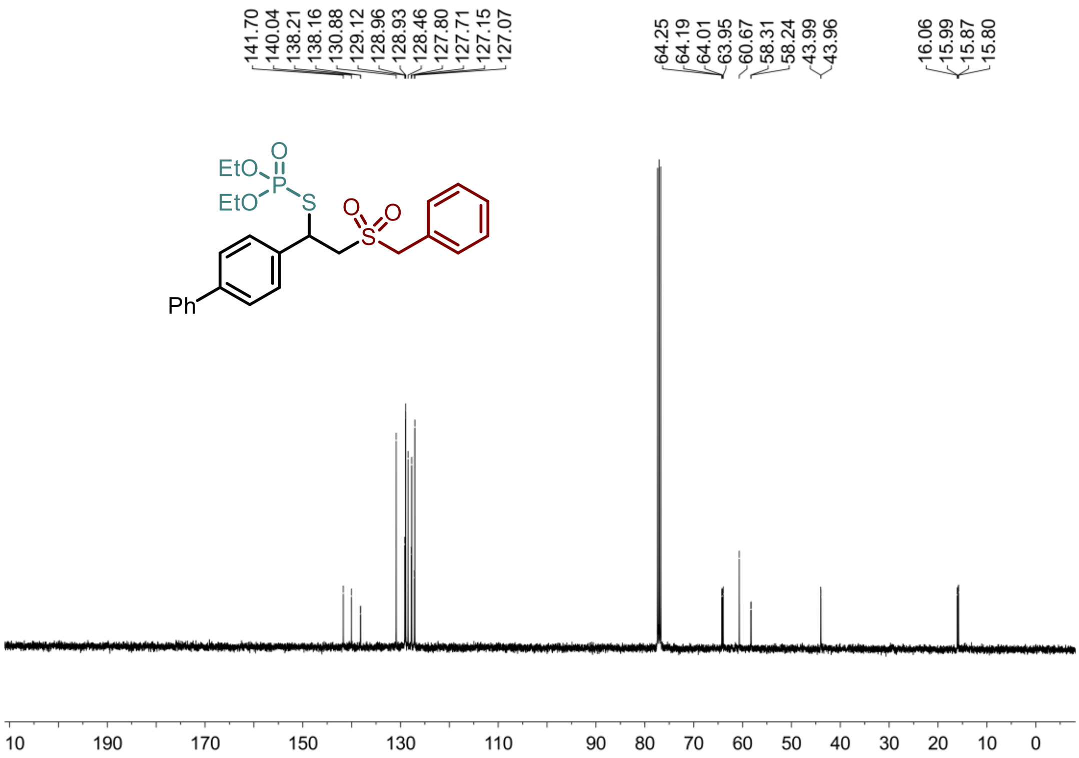

^{1}H NMR spectrum of compound **5m** (400 MHz, $CDCl_3$)

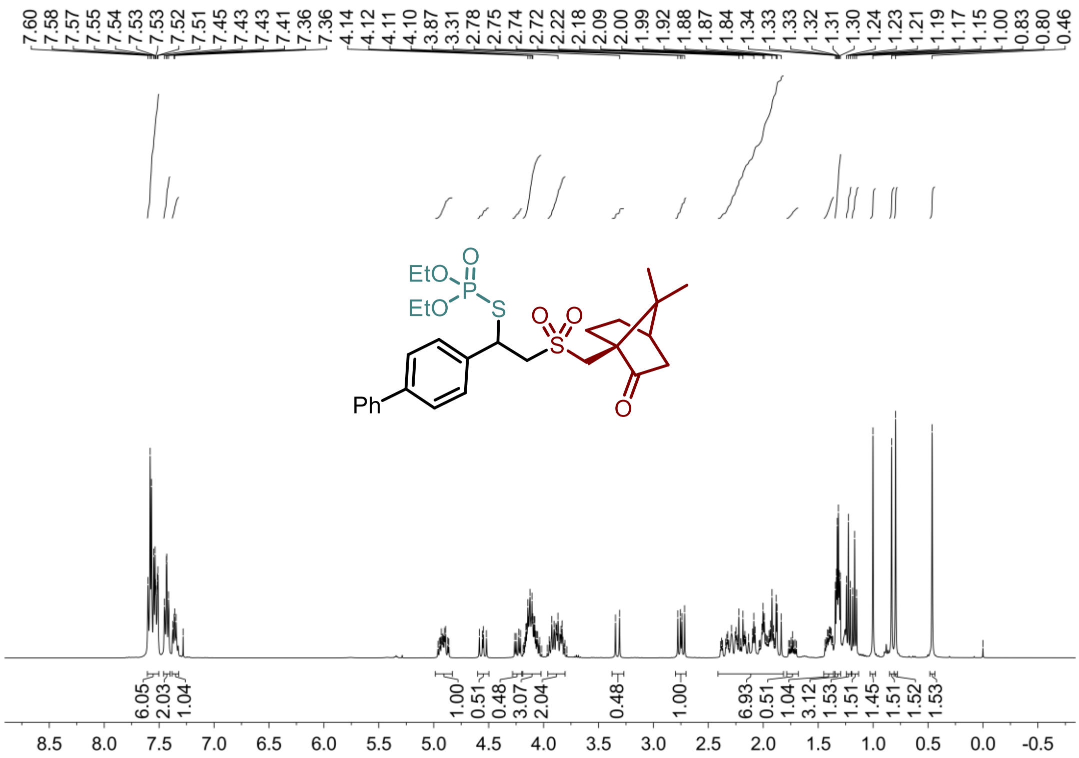

^{13}C NMR spectrum of compound **5m** (100 MHz, $CDCl_3$)

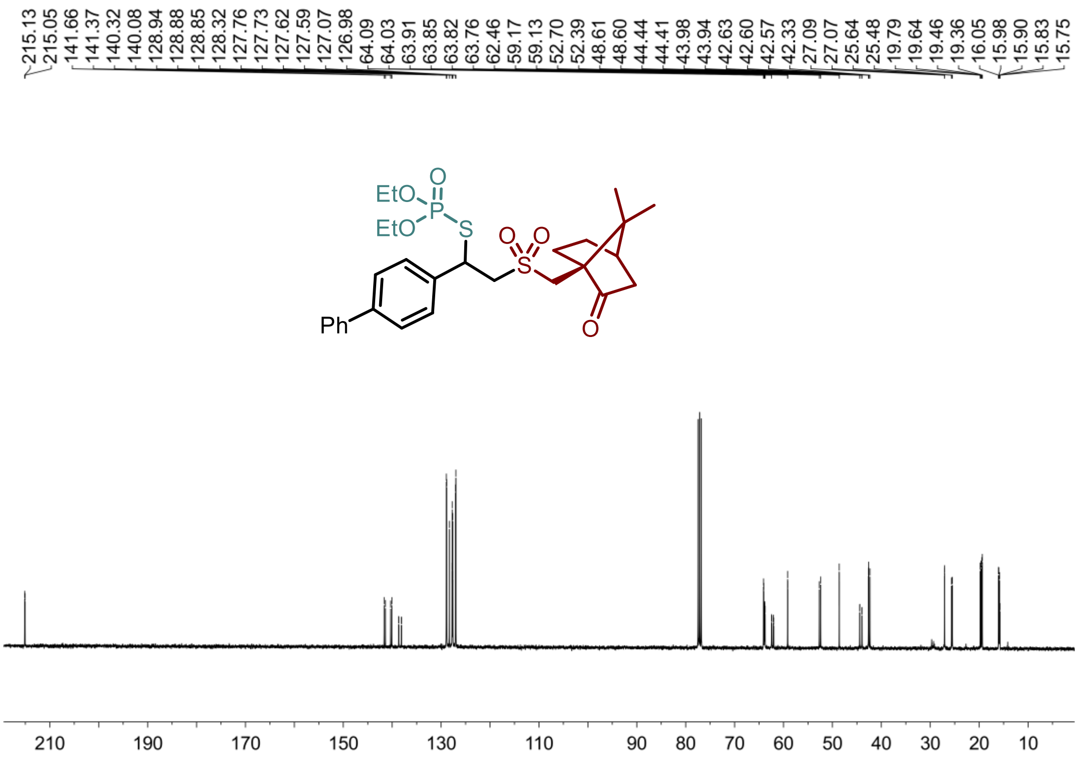

^{1}H NMR spectrum of compound **5n** (400 MHz, $CDCl_3$)

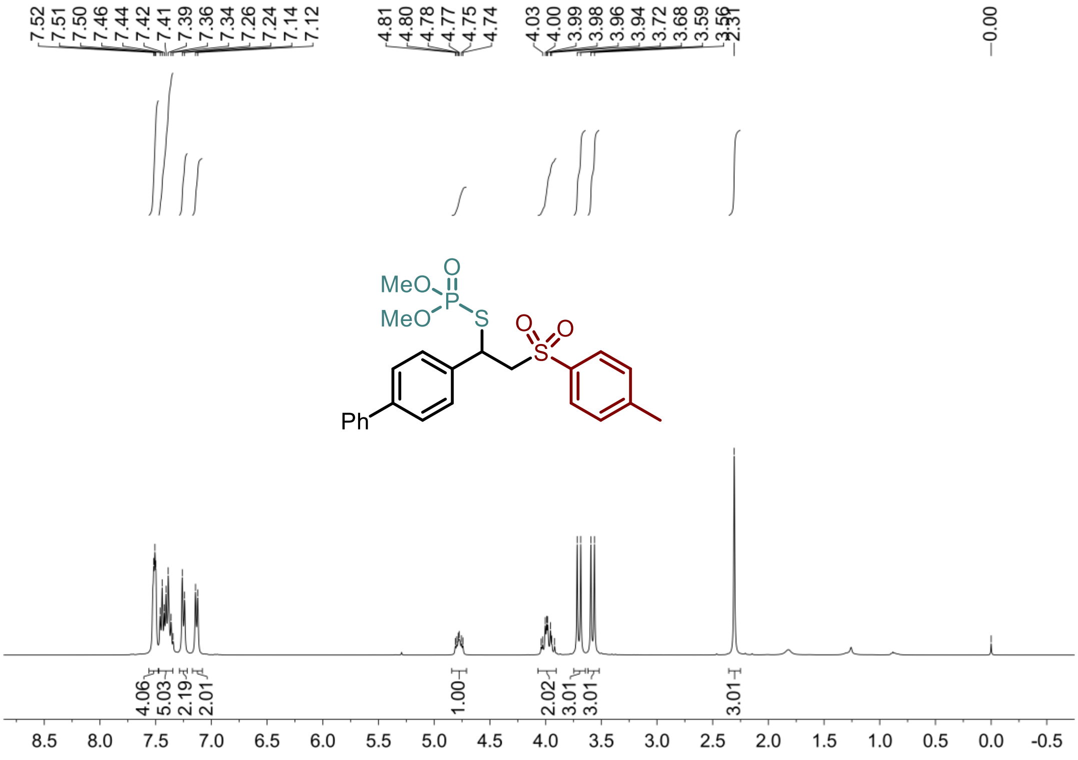

^{13}C NMR spectrum of compound **5n** (100 MHz, $CDCl_3$)

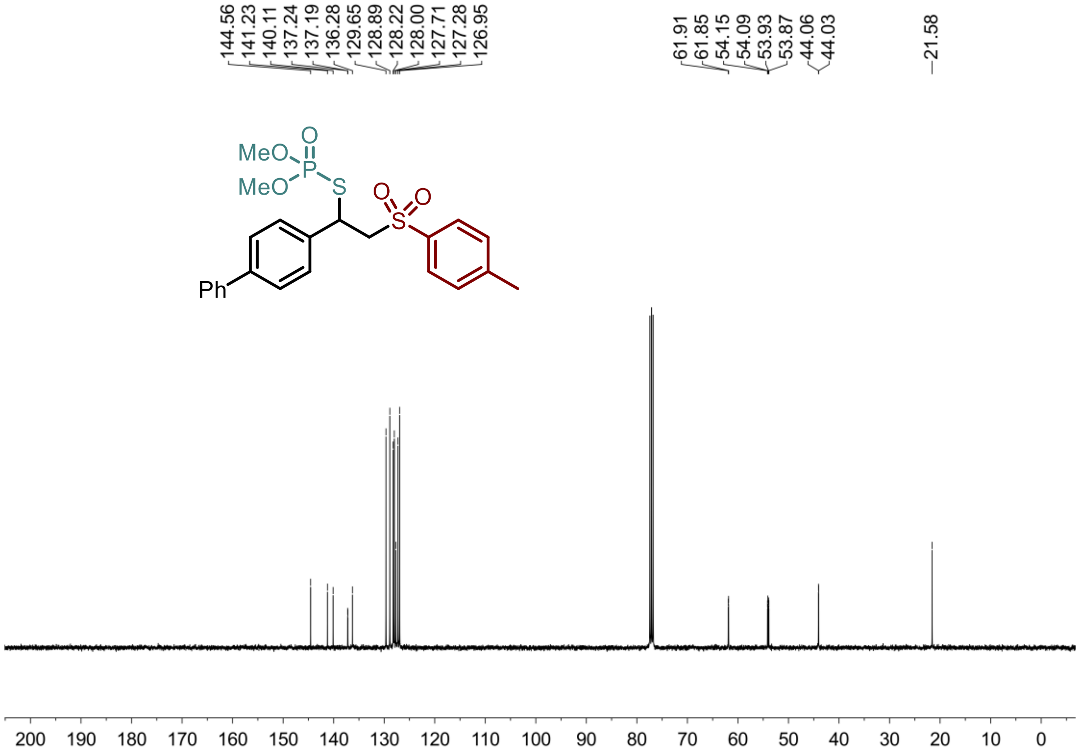

^{1}H NMR spectrum of compound **5o** (400 MHz, $CDCl_3$)

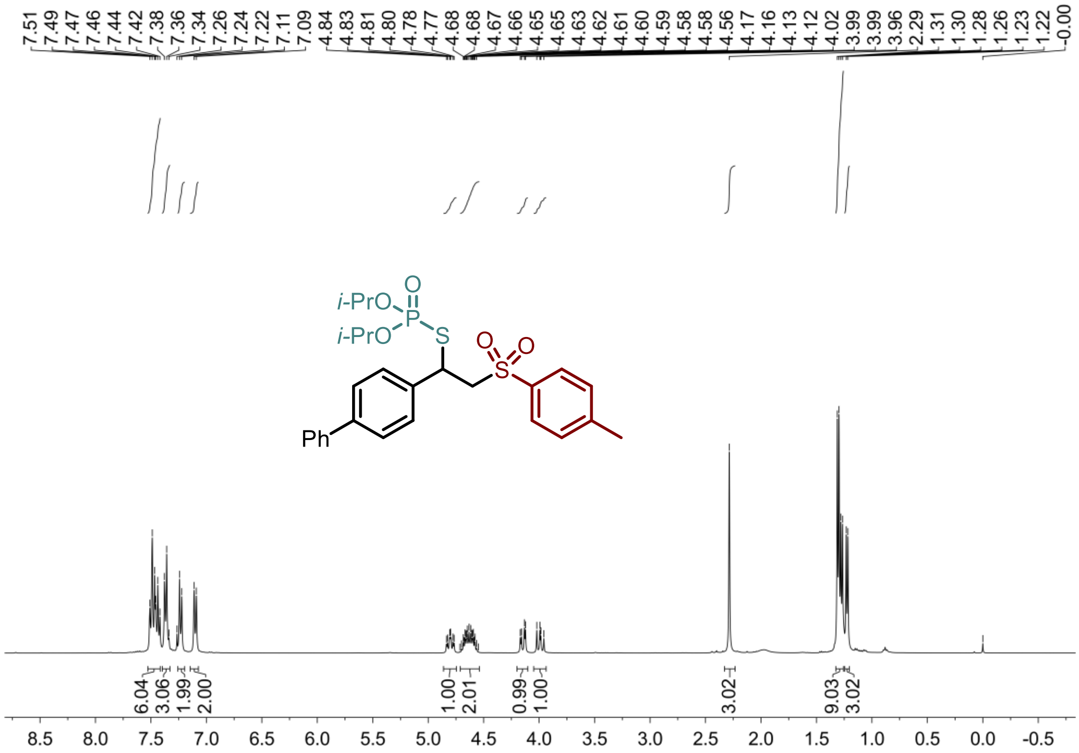

^{13}C NMR spectrum of compound **5o** (100 MHz, $CDCl_3$)

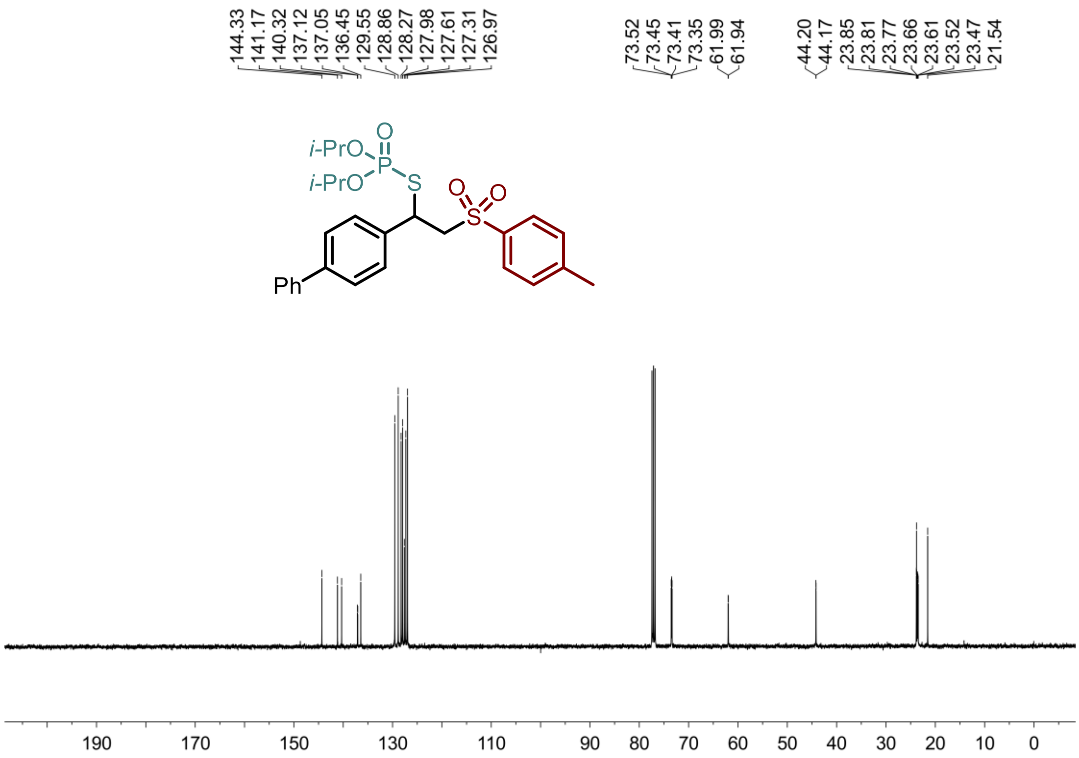

^{1}H NMR spectrum of compound **5p** (400 MHz, $CDCl_3$)

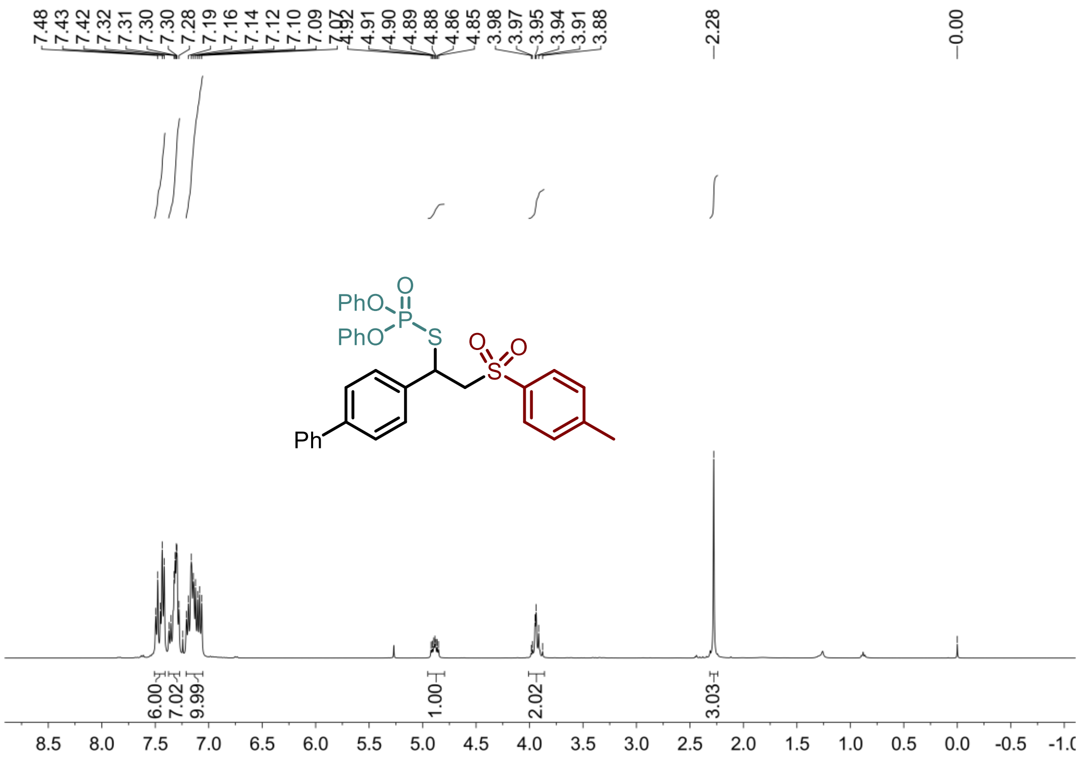

^{13}C NMR spectrum of compound **5p** (100 MHz, $CDCl_3$)

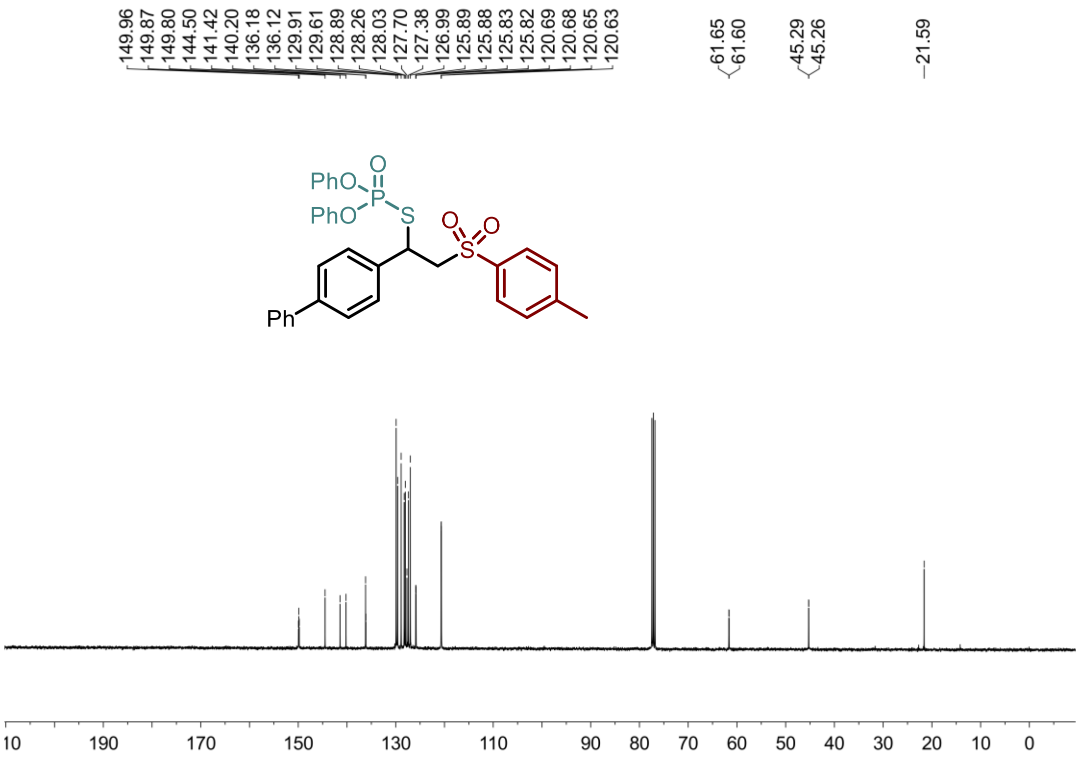

^{1}H NMR spectrum of compound **6a** (400 MHz, $CDCl_3$)

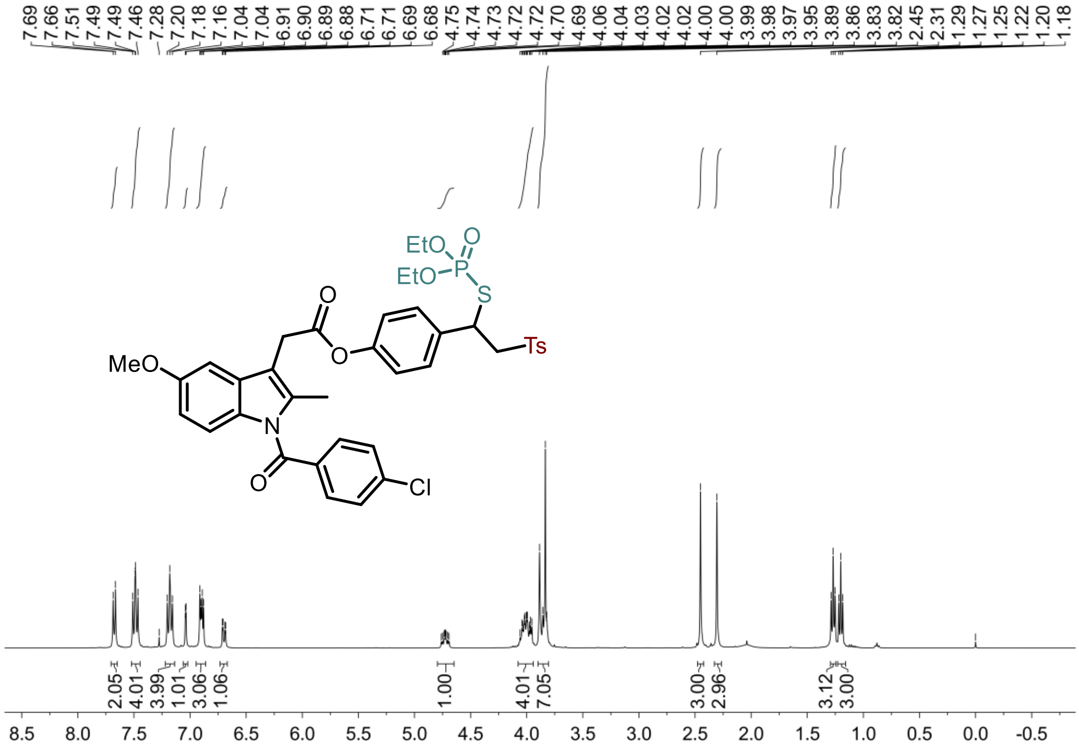

^{13}C NMR spectrum of compound **6a** (100 MHz, $CDCl_3$)

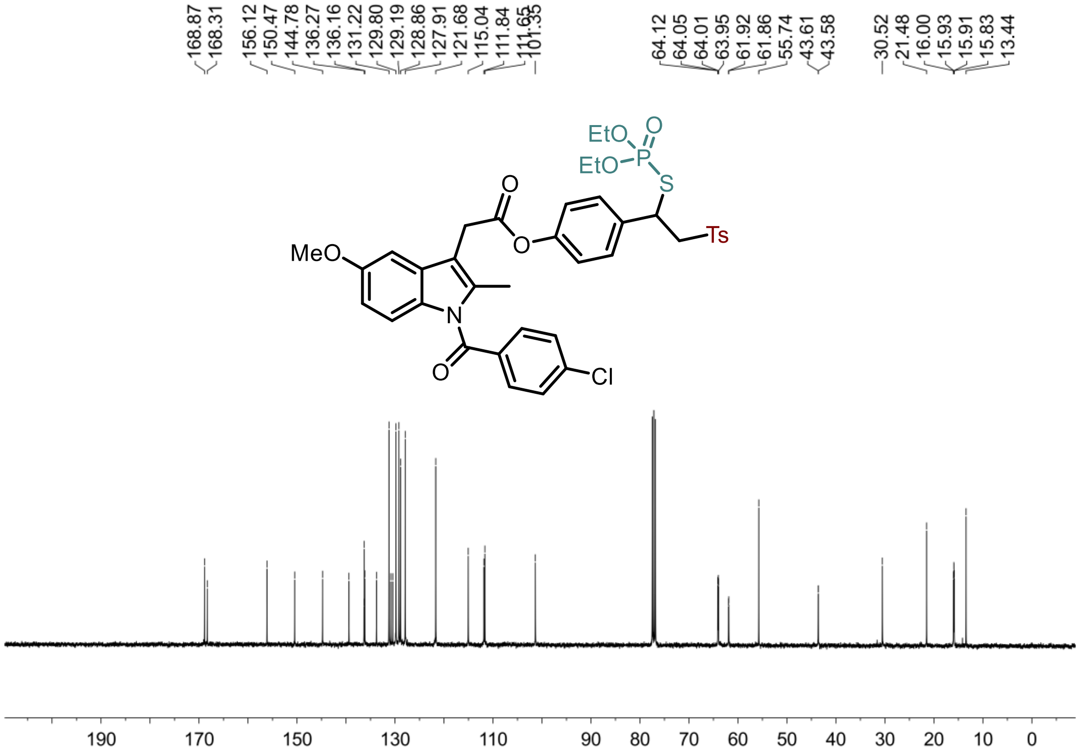

^{1}H NMR spectrum of compound **6b** (400 MHz, $CDCl_3$)

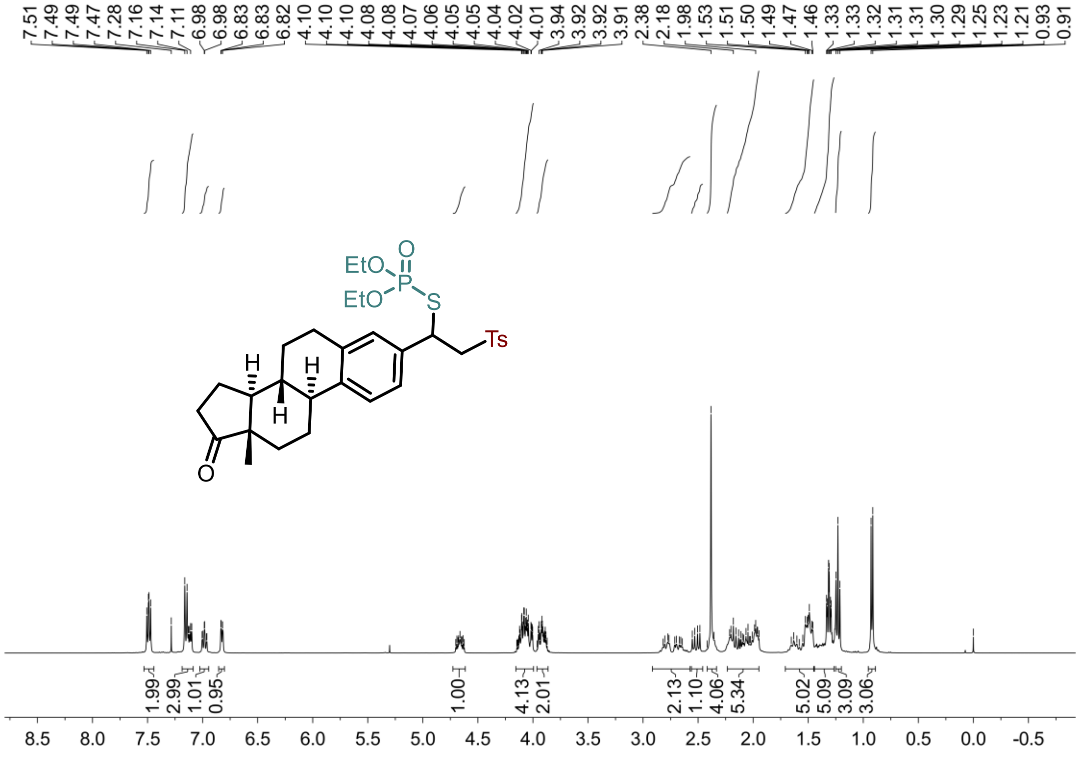

^{13}C NMR spectrum of compound **6b** (100 MHz, $CDCl_3$)

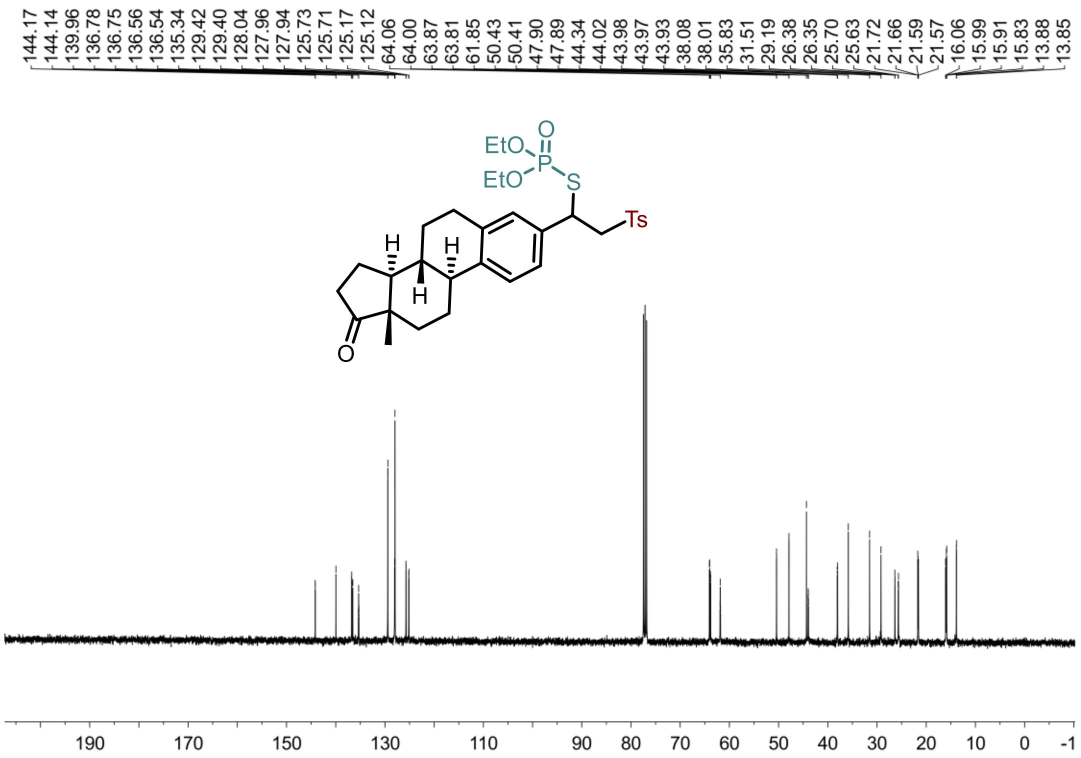

^{1}H NMR spectrum of compound **6c** (400 MHz, $CDCl_3$)

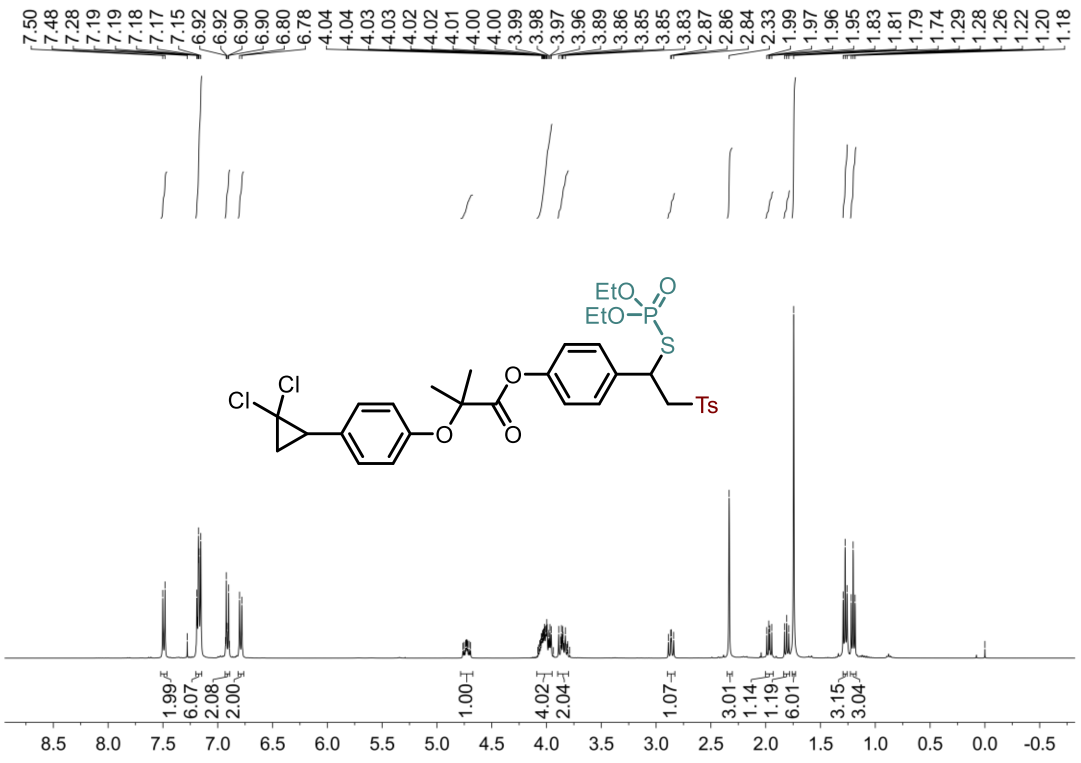

^{13}C NMR spectrum of compound **6c** (100 MHz, $CDCl_3$)

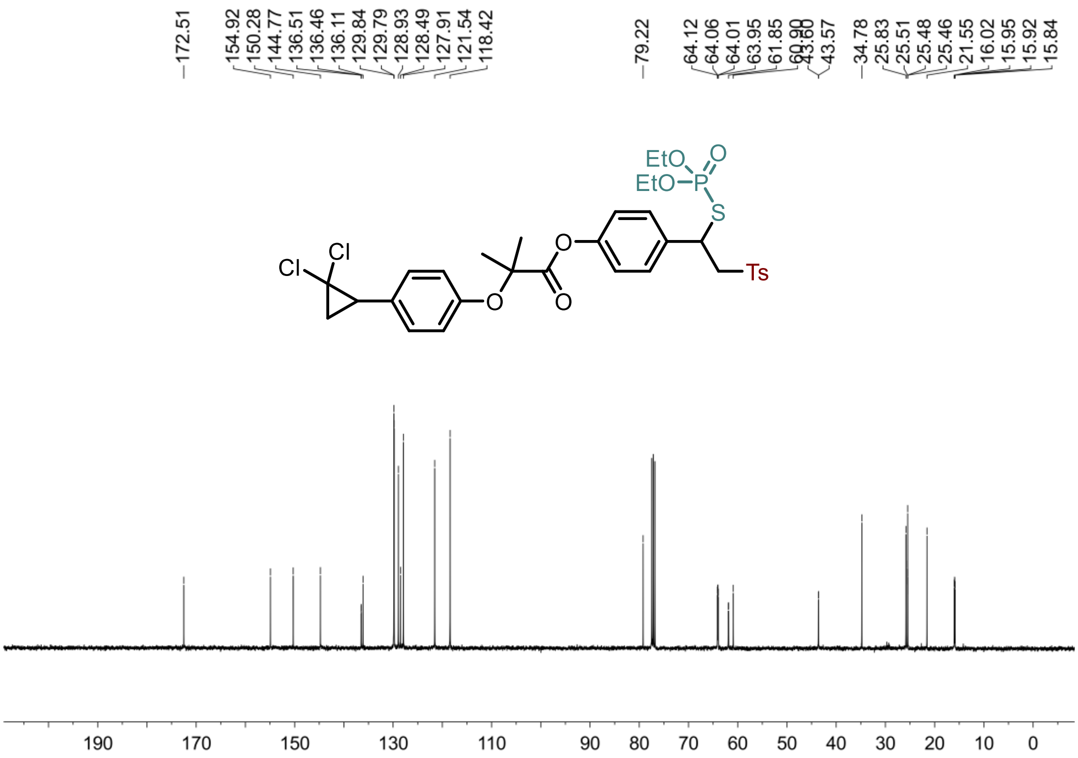

^{1}H NMR spectrum of compound **6d** (400 MHz, $CDCl_3$)

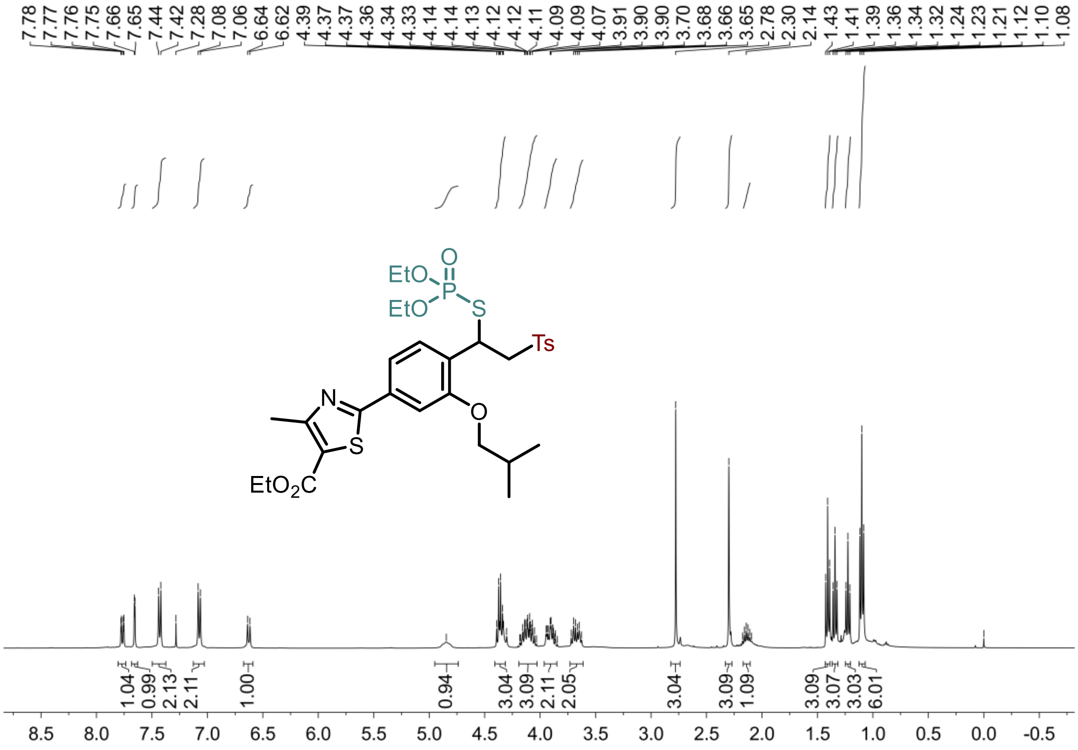

^{13}C NMR spectrum of compound **6d** (100 MHz, $CDCl_3$)

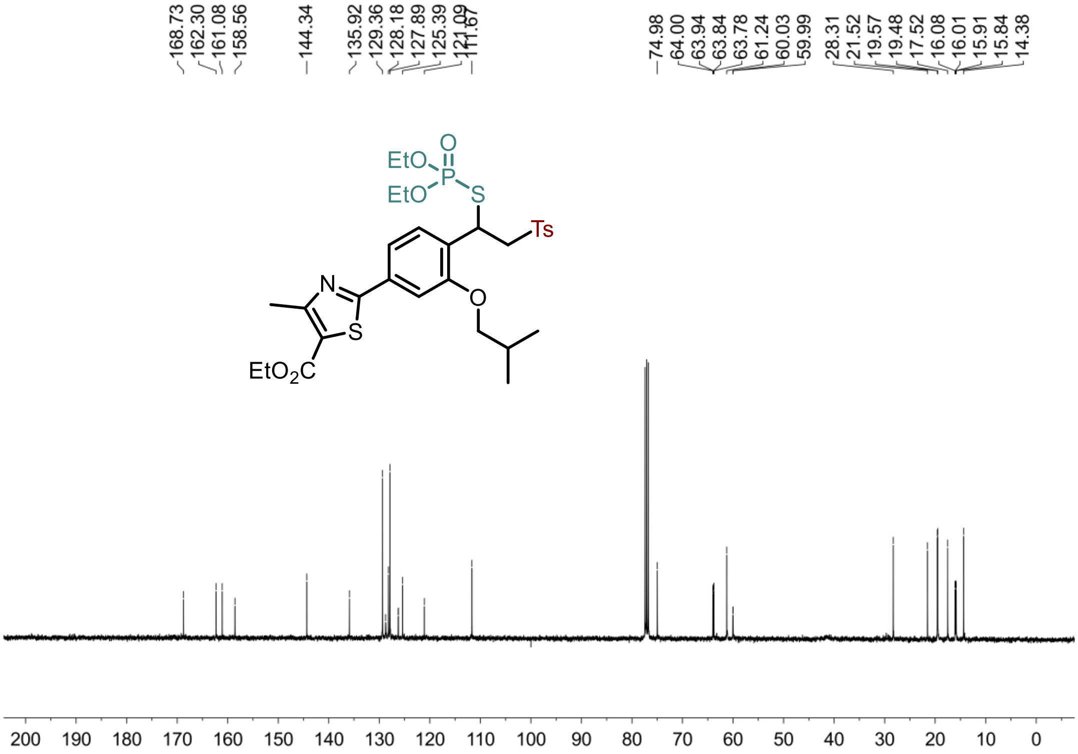

^{1}H NMR spectrum of compound **8** (400 MHz, $CDCl_3$)

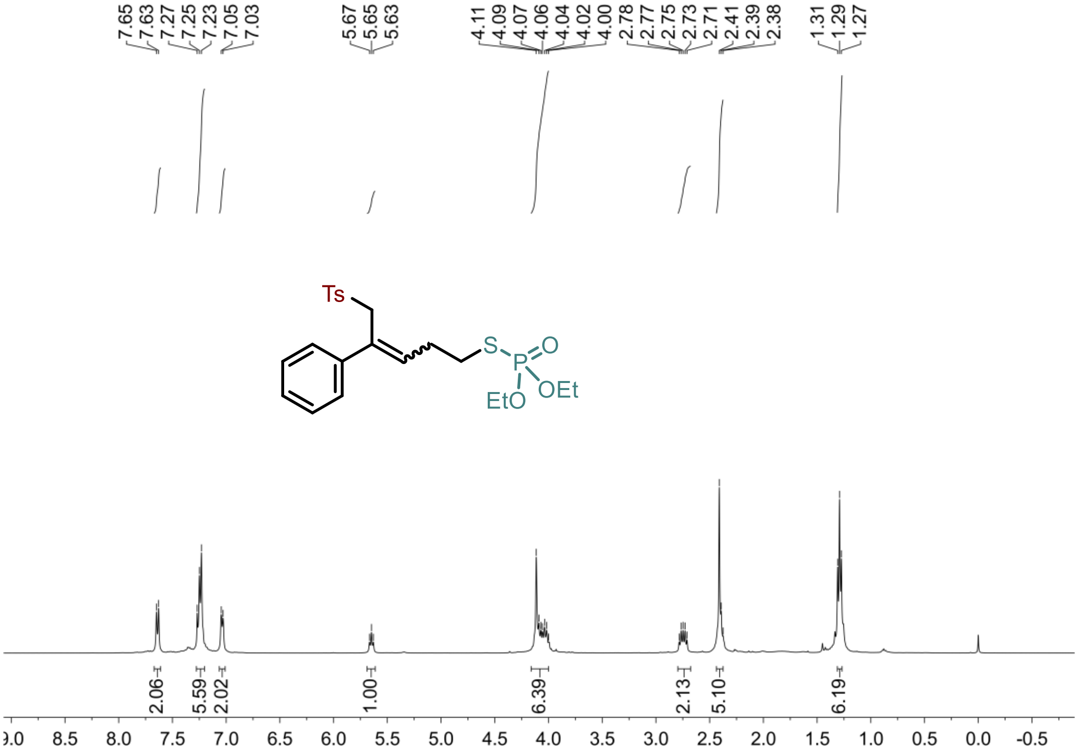

^{13}C NMR spectrum of compound **8** (100 MHz, $CDCl_3$)

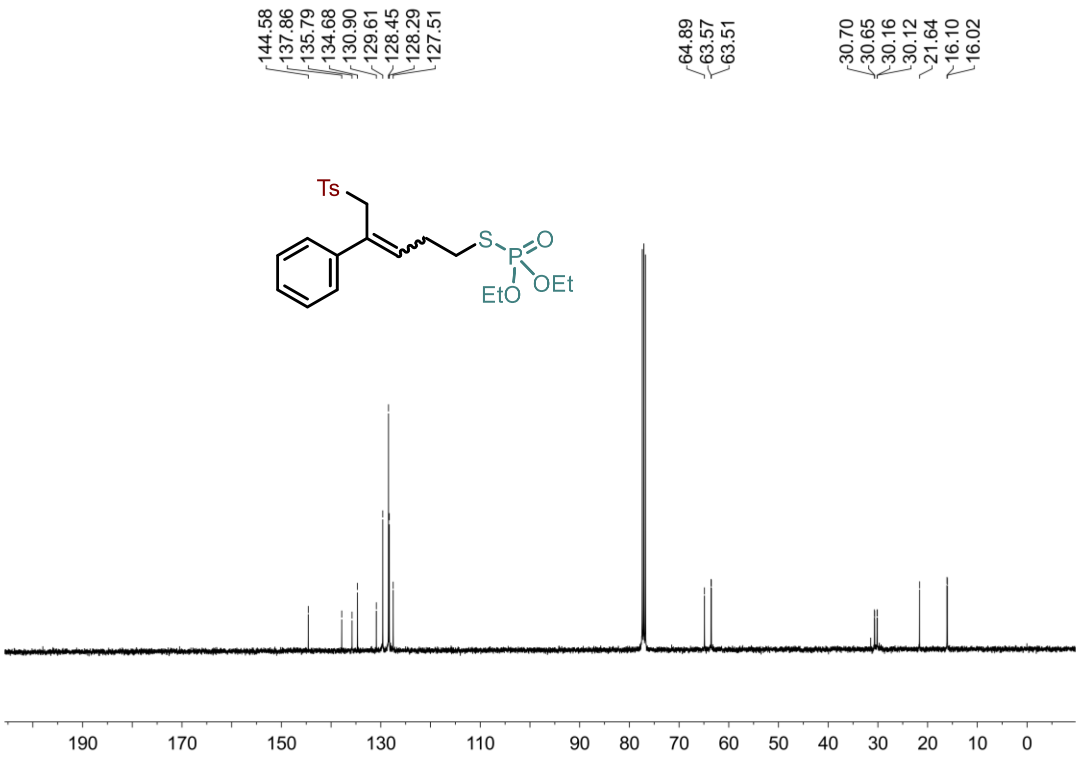

Supplement: Supplementary file 1 [file molecules-28-07869-s001.zip › molecules-2732429-supplementary.pdf]
